# Duphold: scalable, depth-based annotation and curation of high-confidence structural variant calls

**DOI:** 10.1093/gigascience/giz040

**Published:** 2019-04-24

**Authors:** Brent S Pedersen, Aaron R Quinlan

**Affiliations:** 1 Department of Human Genetics, University of Utah, Salt Lake City, UT, 84112; 2 USTAR Center for Genetic Discovery, University of Utah, Salt Lake City, UT, 84112; 3 Department of Biomedical Informatics, University of Utah, Salt Lake City, UT, 84112

**Keywords:** structural variation, genomics, algorithm

## Abstract

Most structural variant (SV) detection methods use clusters of discordant read-pair and split-read alignments to identify variants yet do not integrate depth of sequence coverage as an additional means to support or refute putative events. Here, we present "duphold," a new method to efficiently annotate SV calls with sequence depth information that can add (or remove) confidence to SVs that are predicted to affect copy number. Duphold indicates not only the change in depth across the event but also the presence of a rapid change in depth relative to the regions surrounding the break-points. It uses a unique algorithm that allows the run time to be nearly independent of the number of variants. This performance is important for large, jointly called projects with many samples, each of which must be evaluated at thousands of sites. We show that filtering on duphold annotations can greatly improve the specificity of SV calls. Duphold can annotate SV predictions made from both short-read and long-read sequencing datasets. It is available under the MIT license at https://github.com/brentp/duphold.

## Findings

### Motivation

Structural variants (SVs) are a broad class of genetic variation including duplications, deletions, inversions, insertions, and translocations. SVs are known to be more difficult to detect with high accuracy than single-nucleotide and insertion-deletion variants. As such, the false-positive rate can be high. The most commonly used SV callers [1–5] use 2 types of sequence alignments to discover structural variation: paired-end reads having an unusual orientation or insert size (so-called “discordant pairs”), and split reads, where the sequence is aligned to different parts of the genome. These methods work well, and while some make use of coverage information at the break-points, they do not directly integrate the aligned sequence depth within and around an event to detect or filter SV calls. This is an important limitation because, for example, we expect a true hemizygous deletion to exhibit 50% of the sequence coverage of flanking diploid regions. On the basis of our experience in evaluating the veracity of thousands of candidate SVs with SV-plaudit [6], we noted 2 consistent patterns that distinguished confident deletion and duplication calls from apparent false-positive results. First, events without an obvious reduction or increase in coverage are much less likely to appear as “real” events to the human eye. Second, events with a rapid change in depth at (or near) the break-points are more plausible. Obvious false-positive calls lack either or both of those signals. We therefore developed duphold to enforce the observations we made through manual inspection and rapidly annotate SV calls to prioritize high-quality variant predictions.

### Implementation

The duphold software uses hts-nim [7] to quickly extract coverage information from a BAM or CRAM file into an array using the methodology described in mosdepth [8]. Once in array format, it can be queried very rapidly. The depth profiles are used to quickly annotate a variant call format (VCF) [9] file of structural SVs with coverage calculated from a BAM or CRAM file of alignments. Briefly, duphold operates on each chromosome sequentially; it allocates an (int16) array whose size is the length of the current chromosome (this array uses ∼500 megabytes of memory for the 249 megabase human chromosome 1), iterates over each read in a BAM or CRAM for that chromosome, and increments any bases where an aligned read (or segment of a read) starts and decrements any bases where an aligned read (or part of a read) ends. A segment of a read is defined by the SAM [10] CIGAR operations. Once duphold has processed all segments for all alignments in a chromosome, it performs a cumulative sum that results in a per-base coverage value in the array. A 64-bit integer is used to track the actual depth, but the depth stored on the arrays is capped at the maximum value for a 16-bit integer (32,767) to prevent integer overflow. This algorithm is fully detailed in Pedersen and Quinlan [8]. Once the coverage array is filled, all remaining steps are independent of the number of alignments. Owing to the speed of in-memory array operations, subsequent depth calculations are nearly independent of the number of variants annotated in the VCF file.

For each SV, duphold annotates the VCF sample format field of the variant with both the change in depth relative to the surrounding 1,000 bases on either side of the event and the fold-change in coverage in the event relative to other regions in the genome with similar guanine-cytosine (GC) content. We have evaluated different flanking distances, and 1,000 is sufficient to achieve an accurate estimate of coverage but small enough to avoid commonly unsequenced regions or gaps in coverage. To compare the coverage observed for each variant with genomic bins of similar GC content, duphold calculates the GC content in each non-overlapping, 250-base window in the chromosome along with the median depth in that window. This requires 0.55 CPU-seconds for chromosome 1. These per-window depth and GC values are used as a distribution against which to compare incoming variants.

Once the depths and the GC windows are calculated, duphold uses them to annotate SV calls in VCF format. For each variant, the GC content is calculated for the genome interval defined by the variant, and the median depth inside the event is compared to the window values with a similar GC content to calculate a fold-change value (duphold bin fold-change [DHBFC]). Duphold then compares the median depth in the event to the median depth from the 1,000 bases on either side; this measure (named duphold flank fold-change [DHFFC]) captures the change in depth one would observe by eye upon visual inspection. The depth fold-change values are added to the sample's format information in the variant's VCF entry. Using the median for each metric makes the value more robust even when the reported break-points are inexact or shifted. Duphold is run on a single sample at a time, but it has options to facilitate parallelization across samples. It can run on a 25× whole-genome CRAM in <15 CPU-minutes and run-time will increase linearly with coverage.

### Evaluation

#### Deletions

We evaluated duphold by annotating the LUMPY [1] calls and svtyper [11] genotypes we produced for the HG002 sample sequenced by the Genome in a Bottle [12] (GiaB). We compared these with the GiaB truth set of deletions for the same sample. We used the duphold annotations to filter to more stringent call sets and evaluate both precision and recall. Because duphold does not add any new variants, it can only improve precision, not recall.

The duphold depth annotations enable simple filters that reduce the number of false-positive results while retaining most true-positive results (Table 1). For example, requiring that the fold-change of the deletion relative to the 1,000 bases flanking the deletion be <0.7 (DHFFC < 0.7) removes 61% [(83 – 32)/83] of the false-positive calls while retaining 99% (1,483/1,496) of the true-positive calls. The DHBFC metric measures the depth fold-change relative to bins with a similar GC content and performs similarly. Using more stringent filtering can further reduce the false-positive rate at the expense of the recall. The information used in this filtering is independent of the values reported by LUMPY and svtyper, which do not look at sequence depth metrics.

**Table 1:** Evaluating the accuracy of deletion calls filtered by duphold annotations

Method	False discovery rate	False negative	False positive	True positive	Precision	Recall	F1 score
Unfiltered	0.053	276	83	1,496	0.947	0.844	0.893
DHBFC < 0.7	0.018	298	27	1,474	0.982	0.832	0.901
DHFFC < 0.7	0.021	289	32	1,483	0.979	0.837	0.902

We evaluated deletion calls from LUMPY+svtyper using truvari.py [13] with the GiaB v0.6 truth set. DHBFC: duphold bin fold-change, which compares to regions (bins) of similar GC content. DHFFC: duphold flank fold-change (with 1,000 base flank). This shows that using either DHBFC < 0.7 or DHFFC < 0.7 as a filtering criterion for deletions increases precision, removing 61% [(83 – 32)/83] of false-positive calls while retaining >99% (1,483/1,496) of true-positive calls in the case of using DHFFC.

We examined each of the false-positive calls that remained after duphold filtering. These included a mixture of complex regions that had a loss of coverage, and some that looked as if they could be real variants, but with minimal alignment support. We also visually inspected each of the 13 (i.e., 1,496 – 1,483) true-positive results that duphold marked as low confidence owing to a flank fold-change >0.7 (DHFFC > 0.7). Most of these had a minimal change in coverage that did not meet our threshold, and many looked as if they did not have strong evidence for a call. We even noted 1 variant that looked like a duplication within a deletion, resulting in a copy-neutral event. While these highlight the limitations of a purely depth-based approach, we find the >2-fold reduction in false-positive results, in concert with a retention of 99% of true-positive results, to be a convincing demonstration of duphold’s power to remove the abundant false-positive SV predictions common to most analyses.

#### Duplications

Because LUMPY called only a single duplication in HG002 that was not found in GiaB, we were not able to evaluate the performance of duphold on duplications using that approach. Because the GiaB SV call set does not differentiate insertion events from duplications, we first classified any GiaB insertion as a duplication if the entirety of the reported insertion sequence was mapped by bwa-mem [14] with <5 mismatches to within 3 bases (start and end) of the variant. This resulted in 805 duplications for the truth set.

To evaluate the specificity and sensitivity of duphold, we had to create homozygous reference variants. Specifically, for each heterozygous (0/1) or homozygous alternate (1/1) variant, we simulated a homozygous reference variant of the same size and type (e.g., for a heterozygous duplication, we simulated a homozygous reference duplication) and inserted it into the VCF. We limited the simulated variants to the high-confidence regions provided by GiaB and then retried any variant where >10% of the reference nucleotide sequence inside the simulated event was unknown (“N”). This approach provided a reasonable set of homozygous reference variants of a similar size distribution within the high-confidence GiaB regions.

**Figure 1:**
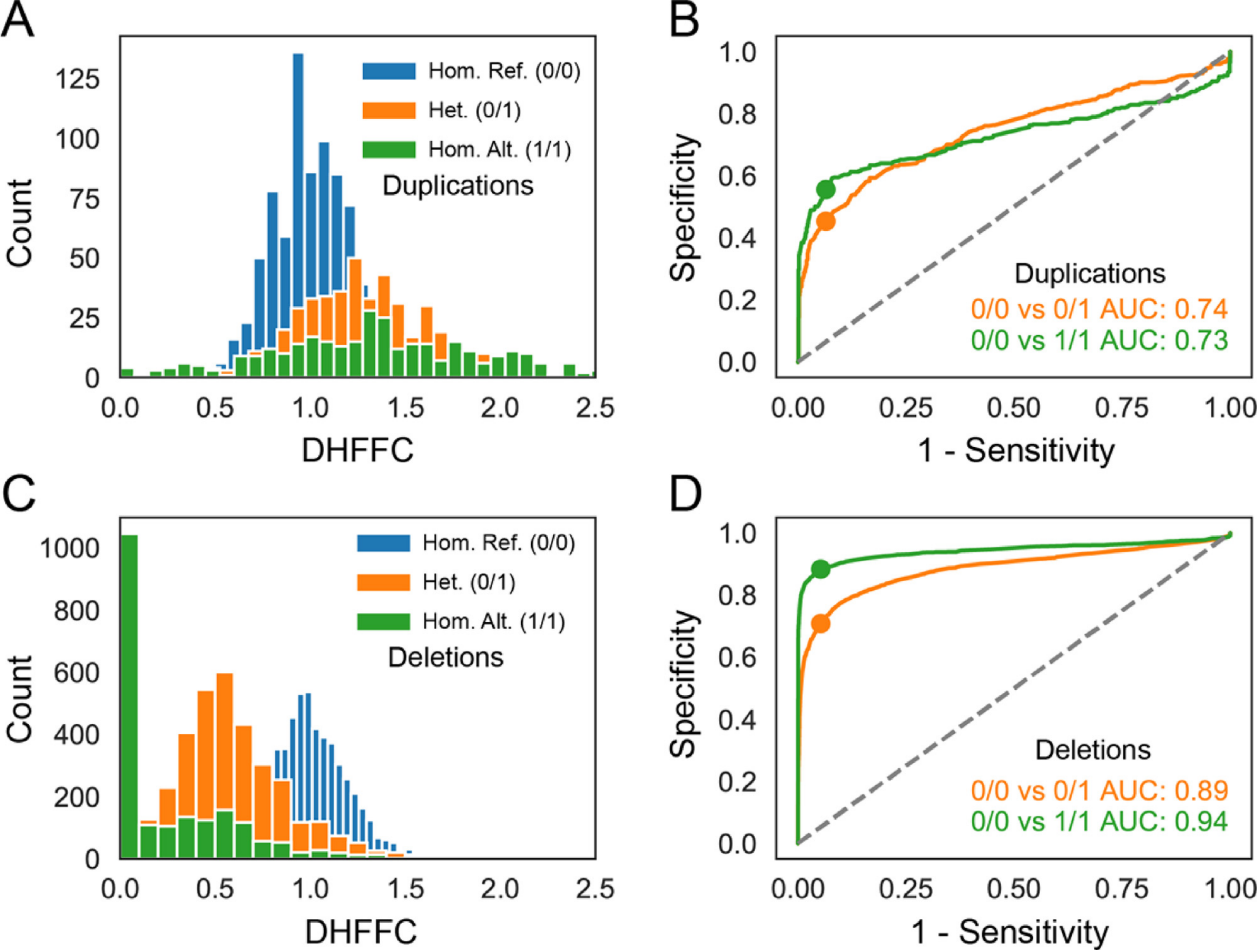
Evaluation of duphold on duplications and deletions of any size. We annotated 805 GiaB insertion calls as duplications and simulated homozygous reference (Hom. Ref.) events of similar size in order to evaluate the specificity and sensitivity of duphold. We show the distribution of DHFFC (duphold flank fold-change) for each genotype (homozygous reference [0/0] is blue, heterozygous [Het.] [0/1] is orange, and homozygous alternate [Hom. Alt.] [1/1] is green), for both duplications (A) and deletions (C). We then used those distributions to create receiver operating characteristic curves (B and D) and calculate AUCs that indicate the ability of duphold to differentiate 0/0 from 0/1 (orange) and 1/1 (green). The dots on the curves indicate a cutoff of 1.3 for duplications and 0.7 for deletions.

**Figure 2:**
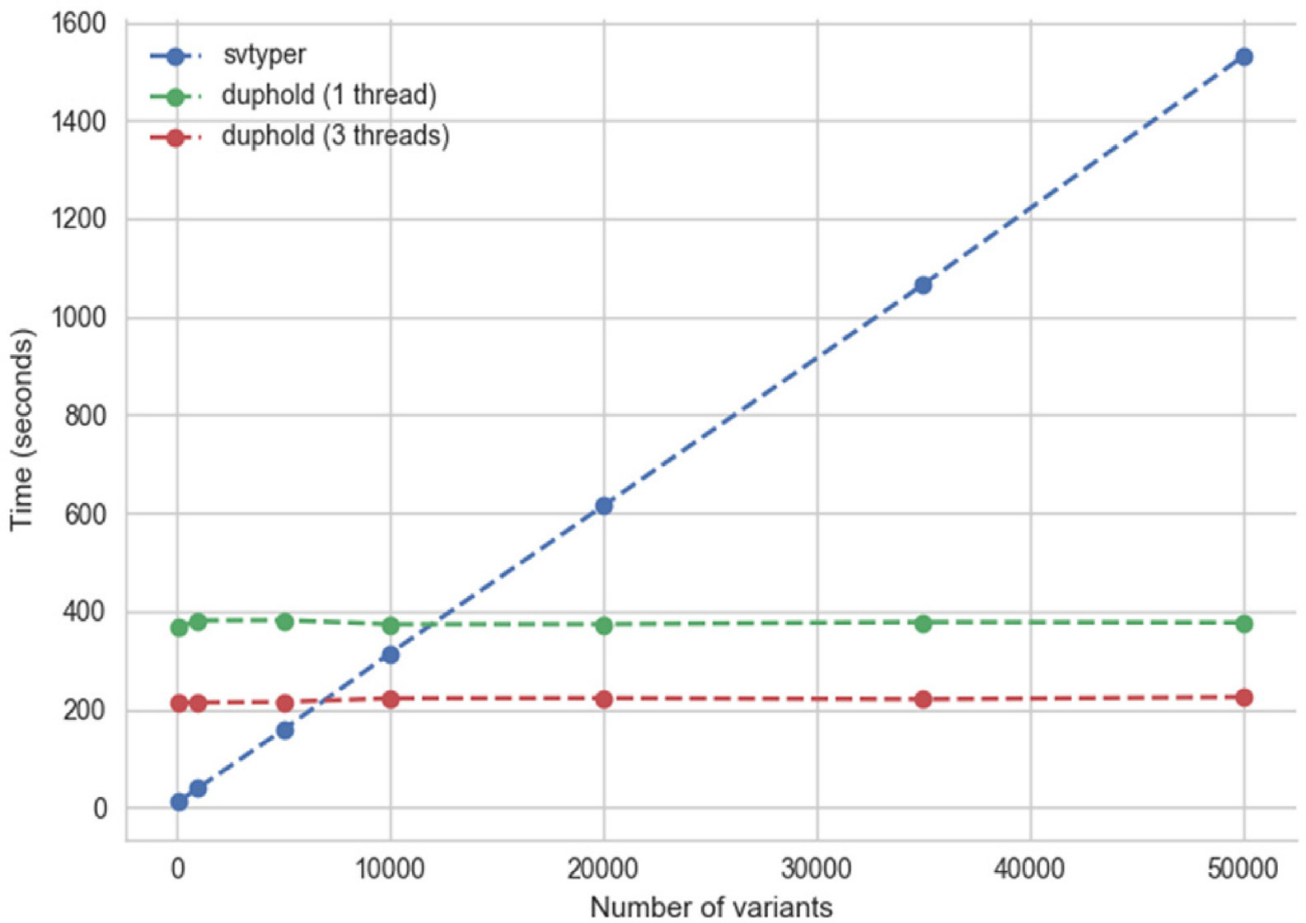
Duphold scalability. The time to annotate (or genotype) for duphold and svtyper is shown (y-axis) as a function of the number of variants tested (x-axis). While svtyper (blue) exhibits a linear increase in type with the number of variants, duphold is relatively independent of the number of variants. There is an initial cost that makes the duphold strategy less efficient for few (less than ∼10,000) variants, but it scales well to annotating thousands of variants as we expect for large cohorts.

We evaluated the sensitivity and specificity of duphold using both the real and simulated deletions and duplications in Figs 1 and 2. While duphold is better able to differentiate deletions from random, copy-neutral locations, it still has an area under the curve (AUC) of 0.74 for heterozygous duplications and 0.73 for homozygous duplications. The dots in the receiver operating characteristic curves show the sensitivity and specificity of duphold at a cutoff of 0.7 for deletions and 1.3 for duplications. The reduced performance on duplications relative to deletions is expected because a heterozygous deletion results in a 2-fold change in depth while a heterozygous duplication results in only a 1.5-fold change. In addition, it could be that a subset of duplications in GiaB, which was created with a combination of technologies, cannot be detected with short-read Illumina data. While the performance shown in Figs 1 and 2 reflects all event sizes, when deletions are restricted to those >1 kilobase, duphold achieves AUCs of 0.97 and 1.0 for heterozygous and homozygous alternate genotypes, respectively. At that size, the number of duplications is too low to properly evaluate, but we expect that larger events will enable duphold to more accurately evaluate the depth inside the event, and therefore further improve performance.

### Scaling

We designed duphold with the expectation that it would be used on large datasets where both specificity and run time are critical. For this reason, we optimized it for situations in which it would be used to evaluate many thousands of variants. In an effort to measure scaling performance, we compared the times of both svtyper and duphold on subsets of the 1,000 Genomes phase 3 SVs [15] (Figs. 1 and 2). We note that we are not interested in the direct time comparison with svtyper because svtyper does more work to genotype the variants. Instead, the relevant pattern is the trajectory in order to demonstrate how well duphold scales. Whereas svtyper follows a linear increase in run time with the number of variants, duphold's performance is nearly independent of the number of variants, using either 1 or 3 threads. This performance is driven by the fact that all of the alignment data are read into efficient data structures that can be queried thousands of times per second. This strategy incurs a large initial cost to construct the data structure and therefore makes duphold less efficient for small variant sets. We have intentionally chosen to optimize for larger variant sets because this context is where efficiency is most important.

## Methods

To evaluate the ability of duphold to prioritize SV calls, we used data from the GiaB project for sample HG002. We downloaded all fastqs from [16], aligned with bwa-mem [14], and marked duplicates with samblaster [17] to generate a CRAM file with ∼25× median sequence coverage. We used the GiaB SV calls and tier 1 regions from [18] as our truth set. We ran LUMPY (LUMPY, RRID:SCR_003253) [1] and svtyper [11] via smoove [19] to create and genotype SV calls. We evaluated the precision and recall before and after applying various filtering on the duphold-annotated variants using truvari [13]. Specifically, we used a modified version of truvari here: [20] to allow “.” filters to be considered as PASS. We used samplot [21] to look at individual variants that were called as true positive, false positive, and false negative.

The truvari command used was as follows:

truvari.py –s 300 –S 270 –b HG002_SVs_Tier1_v0.6.DEL.vcf.gz –c $lumpy_vcf –o eval-no-support –passonly –pctsim = 0 –r 20 –giabreport –f $fasta –no-ref –includebed HG002_SVs_Tier1_v0.6.bed –O 0.6

To demonstrate the utility of duphold on duplication calls, we annotated some GiaB insertion calls as duplications, using [22], and then simulated homozgyous reference calls of the same size and genomic distribution as the existing calls using [23].

To evaluate the scaling on realistic sites, we used duphold to annotate the same HG002 file, but on the 68,818 variants from the 1,000 Genomes SV calls at [24]. We limited those calls to the variants that could be genotyped by svtyper (excluding insertions). We then randomly chose 100, 1,000, 10k, 20k, 35k, and 50k variants and ran svtyper and duphold on each set. We also ran duphold with 3 threads to evaluate the benefit of parallelization.

We downloaded the HG002 single-nucleotide polymorphism/insertion and deletion calls from [25].

## Conclusions

Duphold enables rapid annotation of existing SV calls with sequence depth information that facilitates the distinction between high- and low-confidence deletions and duplications. Using the GiaB truth set, we have shown that we can exclude nearly 61% of false-positive SV predictions while retaining >99% of true-positive variants using a simple filter on a duphold-annotated VCF. Given the minimal additional run time of as few as 25 minutes for a 30× genome, this is a substantial improvement for the overall accuracy of SV call sets.

## Availability of supporting source code and requirements

Project name: duphold

Project home page: https://github.com/brentp/duphold

Operating system(s): binary available for Linux (can be built on OSX and Windows)

Programming language: nim

Other requirements: htslib.so ≥ 1.8

License: MIT

RRID:SCR_016938

## Availability of supporting data

An archival copy of the code is available in the *GigaScience* GigaDB repository [26].

## Abbreviations

AUC: area under the curve; DHBFC: duphold bin fold-change; DHFFC: duphold flank fold-change; GC: guanine-cytosine; GiaB: Genome in a Bottle; SV: structural variant; VCF: variant call format.

## Competing interests

The authors declare that they have no competing interests.

## Funding

B. Pedersen and A. Quinlan were supported by US National Institutes of Health National grants from the National Human Genome Research Institute (R01HG006693 and R01HG009141), the National Institute of General Medical Sciences (R01GM124355), and the National Cancer Institute (U24CA209999).

## Authors' contributions

B.S.P. designed and wrote the software, performed the analyses, and co-wrote the manuscript. A.R.Q. co-wrote the manuscript.

## Supplementary Material

GIGA-D-18-00471_Original_Submission.pdf

GIGA-D-18-00471_Revision_1.pdf

GIGA-D-18-00471_Revision_2.pdf

Response_to_Reviewer_Comments_Original_Submission.pdf

Response_to_Reviewer_Comments_Revision_1.pdf

Reviewer_1_Report_Original_Submission -- Fritz Sedlazeck 12/20/2018 Reviewed

Reviewer_1_Report_Revision_1 -- Fritz Sedlazeck 2/14/2019 Reviewed

Reviewer_2_Report_Original_Submission -- Xuefang Zhao 1/13/2019 Reviewed

Reviewer_2_Report_Revision_1 -- Xuefang Zhao 2/24/2019 Reviewed

Supplemental Files
