# Supplementary material for: Duphold: scalable, depth-based annotation and curation of high-confidence structural variant calls
Source: Gigascience. 2019 Apr 24;8(4):giz040. doi: 10.1093/gigascience/giz040 (PMC6479422; doi:10.1093/gigascience/giz040)
Supplement: GIGA-D-18-00471_Original_Submission.pdf [file giz040_giga-d-18-00471_original_submission.pdf]

GigaScience

duphold: scalalable, depth-based annotation and curation of high-confidence structural variant calls

--Manuscript Draft--

**Manuscript Number:**	GIGA-D-18-00471
**Full Title:**	duphold: scalalable, depth-based annotation and curation of high-confidence structural variant calls
**Article Type:**	Technical Note
**Funding Information:**	National Human Genome Research Institute (R01HG006693) — Dr Aaron R Quinlan<br>National Human Genome Research Institute (R01HG009141) — Dr Aaron R Quinlan<br>National Institute of General Medical Sciences (R01GM124355) — Dr Aaron R Quinlan<br>National Cancer Institute (U24CA209999) — Dr Aaron R Quinlan
**Abstract:**	Most structural variant detection tools use clusters of discordant read-pair and split-read alignments to identify variants, yet do not integrate depth of sequence coverage as an additional means to support or refute putative events. Here, we present duphold, as a new method to efficiently annotate structural variant calls with sequence depth information that can add (or remove) confidence to SV predicted to affect copy number. It indicates not only the change in depth across the event, but also the presence of a rapid change in depth relative to the regions surrounding the breakpoints. It uses a unique algorithm that allows the run time to be nearly independent of the number of variants. This performance is important for large, jointly-called projects with many samples, each of which must be evaluated at thousands of sites. We show that filtering on duphold annotations can greatly improve the specificity of deletion calls and that its annotations match visual inspection. Duphold can annotate structural variant predictions made from both short-read and long-read data. It is available under the MIT license at: https://github.com/brentp/duphold .
**Corresponding Author:**	Brent Pedersen<br>University of Utah Hospital<br>UNITED STATES
**Corresponding Author Secondary Information:**	
**Corresponding Author's Institution:**	University of Utah Hospital
**Corresponding Author's Secondary Institution:**	
**First Author:**	Brent Pedersen
**First Author Secondary Information:**	
**Order of Authors:**	Brent Pedersen
	Aaron R Quinlan, Phd
**Order of Authors Secondary Information:**	

Powered by Editorial Manager® and ProduXion Manager® from Aries Systems Corporation

Additional Information:	
**Question**	**Response**
Are you submitting this manuscript to a special series or article collection?	No
**Experimental design and statistics**<br><br>Full details of the experimental design and statistical methods used should be given in the Methods section, as detailed in our Minimum Standards Reporting Checklist. Information essential to interpreting the data presented should be made available in the figure legends.<br><br>Have you included all the information requested in your manuscript?	Yes
**Resources**<br><br>A description of all resources used, including antibodies, cell lines, animals and software tools, with enough information to allow them to be uniquely identified, should be included in the Methods section. Authors are strongly encouraged to cite Research Resource Identifiers (RRIDs) for antibodies, model organisms and tools, where possible.<br><br>Have you included the information requested as detailed in our Minimum Standards Reporting Checklist?	Yes
**Availability of data and materials**<br><br>All datasets and code on which the conclusions of the paper rely must be either included in your submission or deposited in publicly available repositories (where available and ethically appropriate), referencing such data using a unique identifier in the references and in the "Availability of Data and Materials" section of your manuscript.	Yes

Have you have met the above requirement as detailed in our Minimum Standards Reporting Checklist?	

Powered by Editorial Manager® and ProduXion Manager® from Aries Systems Corporation

Click here to download Manuscript duphold manuscript.pdf

duphold: scalalable, depth-based annotation and curation of high-confidence structural variant calls.

Brent S. Pedersen1,3* and Aaron R. Quinlan1,2,3*
1 Department of Human Genetics, University of Utah. Salt Lake City, UT
2 Department of Biomedical Informatics, University of Utah. Salt Lake City, UT
3 USTAR Center for Genetic Discovery, University of Utah. Salt Lake City, UT

* to whom correspondence should be addressed

Abstract

Most structural variant detection tools use clusters of discordant read-pair and split-read alignments to identify variants, yet do not integrate depth of sequence coverage as an additional means to support or refute putative events. Here, we present *duphold,* as a new method to efficiently annotate structural variant calls with sequence depth information that can add (or remove) confidence to SV predicted to affect copy number. It indicates not only the change in depth across the event, but also the presence of a rapid change in depth relative to the regions surrounding the breakpoints. It uses a unique algorithm that allows the run time to be nearly independent of the number of variants. This performance is important for large, jointly-called projects with many samples, each of which must be evaluated at thousands of sites. We show that filtering on *duphold* annotations can greatly improve the specificity of deletion calls and that its annotations match visual inspection. Duphold can annotate structural variant predictions made from both short-read and long-read data. It is available under the MIT license at: https://github.com/brentp/duphold.

Keywords

Structural-Variation
Genomics
Algorithm

Findings

Motivation

Structural variants (SV) are a broad class of genetic variation including duplications, deletions, inversions, insertions, and translocations. SVs are known to be more difficult to detect with high accuracy than single-nucleotide and insertion-deletion variants. As such, the false positive rate can be high, so it is difficult to separate true variants of interest from those that might be due to noise. The most commonly used structural variant callers[1–5] use two types of sequence alignments to discover structural variation: paired-end reads having an unusual orientation or insert size (so called "discordant pairs"), and split-reads, where the sequence is aligned to different parts of the genome. These methods work well, but do not use the aligned sequence depth to detect or filter structural variant calls. This is an important limitation, since, for example, we expect a true hemizygous deletion to exhibit 50% of the sequence coverage of flanking diploid regions. Our experience in evaluating thousands of candidate SVs with SVPlaudit[6], which allows users to quickly assess the veracity of

structural variants, we noted two consistent patterns that distinguished confident deletion and duplication calls from apparent false positives. First, events without an obvious reduction or increase in coverage are much less likely to appear as "real" events to the human eye. Second, events with a rapid change in depth at (or near) the breakpoints are more plausible. Obvious false positive calls lack either or both of those signals. Unfortunately, most SV discovery tools do not look at the coverage information required to discover these signals. We therefore developed *duphold* to enforce the observations we made through manual inspection and rapidly annotate SV calls in order to prioritize high-quality variant calls.

Implementation

duphold uses our previously described hts-nim[7] library to quickly extract coverage information from a BAM or CRAM file into an array where it can be queried directly. These depth profiles are used to quickly annotate a VCF[8] file of structural variants with coverage calculated from a BAM or CRAM file of alignments. Briefly, *duphold* allocates an (int16) array whose size is the length of the current chromosome (this array uses about 500 megabytes of memory for the 249 megabase human chromosome 1), iterates over each read in a BAM or CRAM for that chromosome, and increments any bases where a read (or segment of a read) starts and decrements any bases where a read (or part of a read) ends. A segment of a read is defined by the SAM[9] CIGAR operations. Once *duphold* has processed all segments for all alignments in a chromosome, it performs a cumulative sum which results in a per-base coverage value in the array. A 64 bit integer is used to track the actual depth but the depth stored on the arrays is capped at at the maximum value for a 16 bit integer (32767) to prevent integer overflow. This algorithm is fully detailed in the mosdepth manuscript[10]. Once the coverage array is filled, all remaining steps are independent of the number of alignments. Owing to the speed of in-memory array operations, subsequent depth calculations are nearly independent of the number of variants annotated in the VCF file.

For each structural variant, *duphold* annotates the VCF sample format field of the variant with both the change in depth relative to the surrounding 5000 bases on either side of the event, and the fold-change in coverage in the breakpoint relative to other regions in the genome with similar GC-content. In order to compare the coverage observed for each variant with genomic bins of similar GC-content, duphold calculates the GC-content in each 250 base window (the window size is configurable) in the chromosome along with the median depth in that window. This requires 0.55 CPU-seconds for chromosome 1. These per-window depth and GC values are used as a distribution against which to compare incoming variants.

Once the depths and the GC-windows are calculated, we use hts-nim to read and annotate structural variant calls in VCF format . For each variant, the GC-content from start to end is calculated, and the median depth inside the event is compared to the window values with a similar GC-content to calculate a fold-change value (DHBFC for duphold bin fold-change). *Duphold* then compares the median depth in the event to the median depth from the 5000 bases on either side; this measure (named DHFFC, for duphold flank fold-change) captures the change in depth one would observe by eye upon visual inspection. The depth fold-change values are added to the sample's format information in the variant's VCF entry. *Duphold* is run on a single-sample at a time, but it has support for facile parallelization across samples. It can run on a 25X whole genome CRAM in < 15 CPU-minutes.

Evaluation

Deletions

We evaluated *duphold* by annotating the lumpy[1] calls and svtyper[11] genotypes we produced for the HG002 sample sequenced by the Genome in a Bottle[12] (GiaB). We compared these to the GiaB truth-set of deletions for the same sample. We used the *duphold* annotations to filter to more stringent call sets and evaluate the precision and recall. Because *duphold* does not add any new variants, it can only improve precision, not recall.

Method	FDR	FN	FP	TP-call	Precision	Recall	F1-score
Unfiltered	0.067	900	133	1849	0.933	0.673	0.782
DHBFC < 0.7	0.023	944	43	1805	0.977	0.657	0.785
DHFFC < 0.7	0.026	932	48	1817	0.974	0.661	0.788

Table 1. Evaluating accuracy of calls filtered by duphold annotations. We evaluated deletion calls from lumpy+svtyper using truvari.py (https://github.com/spiralgenetics/truvari) with the GiaB v0.6 truthset. Columns are FDR: false discovery rate, FN: false negatives, FP: false positive, TP: true-positive, precision, recall, and F1 score. This shows that using either the DHBFC < 0.7 or DHFFC < 0.7 as a filtering criteria for deletions increases precision, removing 64% (1 - 48 / 133)) of false positive calls while retaining about 98% (1817 / 1849) of true positive calls in the case of using DHFFC.

The duphold depth annotations enable simple filters that reduce the number of false-positives while retaining most true positives (**Table 1**). For example, requiring that the fold-change of the deletion relative to the 5000 bases flanking the deletion must be less than 0.7 (DHFFC < 0.7) removes only 85 of the original 133 false positive calls (64%), while retaining 1817 of the original 1849 true positive calls (98%). The DHBFC metric measures the depth fold-change relative to bins with a similar GC-content, and performs similarly. Using more stringent filtering can further reduce the false positive rate at the expense of the recall. The information used in this filtering is independent of the values reported by lumpy and svtyper which do not look at sequence depth metrics.

We examined each of the 48 false positive calls that remained after *duphold* filtering. These included a mixture of complex regions that had a loss of coverage, and some that looked like they could be real variants, but with minimal alignment support. We also visually inspected each of the 32 (i.e., 1849 - 1817) true positives that *duphold* marked as low confidence owing to a flank fold-change greater than 0.7 (DHFFC > 0.7). Most of these had a minimal change in coverage that did not meet our threshold and many looked like they did not have strong evidence for a call. We even noted one variant that looked like a duplication within a deletion, resulting in a copy-neutral event. While these highlight the limitations of a purely depth-based approach, we find that the more than 2-fold reduction in false positives in concert with a retention of nearly 99% of true-positives to be a convincing demonstration of *duphold*'s power to remove the abundant false positive SV prediction common to most analyses.

Scaling

We designed *duphold* with the expectation that it would be used on large datasets where specificity and run time are both critical. For this reason, we optimized it for situations where it would be used to evaluate many thousands of variants. In an effort to measure scaling performance, compared the times of both svtyper and duphold on subsets of the thousand genomes phase 3 structural variants (**Figure 1**). We are not interested in the direct time comparison with svtyper (since svtyper does more work to genotype the variants). Instead, the relevant pattern is the trajectory in order to demonstrate how well *duphold* scales. Svtyper follows a linear increase in time with the number of variants, while *duphold's* performance is nearly independent of the number of variants, using either a single (green) or 3 (red) threads. This performance is driven by the fact that all of the alignment data is read into efficient data structures that can be queried thousands of times a second. This strategy incurs a large initial cost to construct the data structure, and therefore makes *duphold* less efficient for small variant sets. We have chosen to optimize for larger variant sets, since this context is where efficiency is most important.

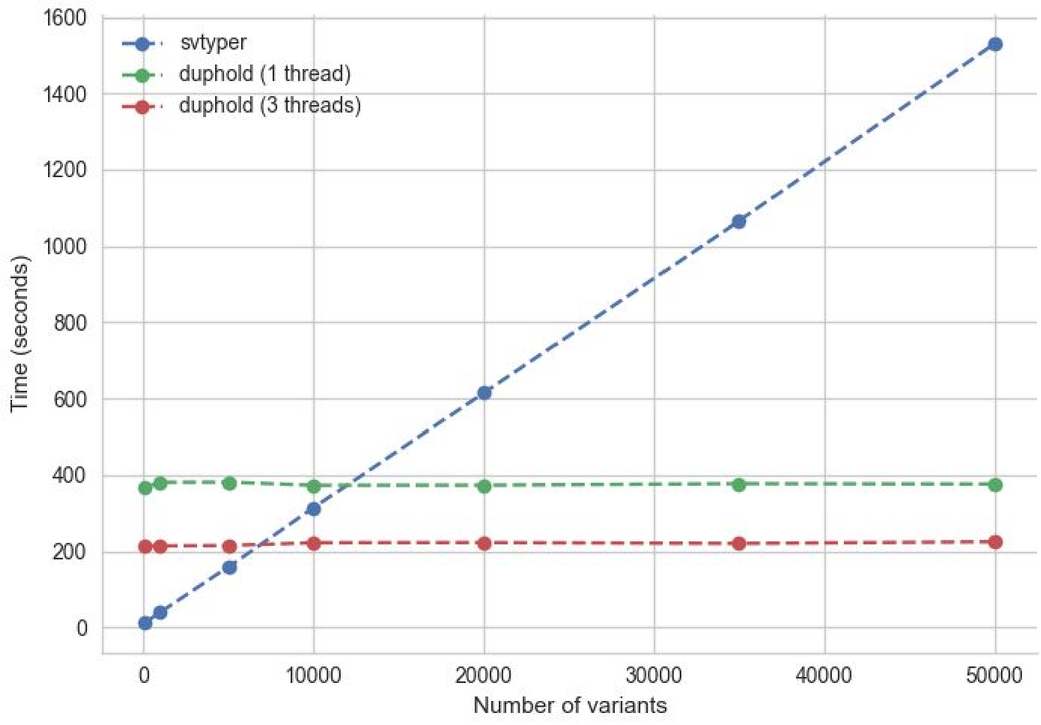

Figure 1. ***Duphold scalability.*** The time to annotate (or genotype) for duphold and svtyper is shown (y-axis) as a function of the number of variants tested (x-axis). While svtyper (blue) exhibits a linear increase in type with the number of variants, *duphold* is relatively independent of the number of variants. There is an initial cost that makes the *duphold* strategy less efficient for few (less than about 10K) variants but it scales well to annotating thousands of variants as we expect for large cohorts.

Methods

To evaluate the ability of *duphold* to prioritize structural variant calls, we used data from the Genome in a Bottle project for sample HG002. We downloaded all fastqs from: ftp://ftp-trace.ncbi.nlm.nih.gov/giab/ftp/data/AshkenazimTrio/HG002_NA24385_son/NIST_Hi

Seq_HG002_Homogeneity-10953946/HG002_HiSeq300x_fastq/140528_D00360_0018_AH8VC6ADXX/ ,aligned with bwa-mem[13], and marked duplicates with samblaster[14] to generate a CRAM file with ~25X median sequence coverage. We used the GiaB SV calls from ftp://ftptrace.ncbi.nlm.nih.gov/giab/ftp/data/AshkenazimTrio/analysis/NIST_SVs_Integration_v0.6/ as our truth-set. Because that does not explicitly differentiate insertions from duplications, we limited our evaluation to duplications. We ran lumpy[1] and svtyper[11] via smoove (https://github.com/brentp/smoove) to create and genotype structural variant calls. We evaluated the precision and recall before and after applying various filtering on the duphold annotated variants using truvari (https://github.com/spiralgenetics/truvari). We used samplot (https://github.com/ryanlayer/samplot) to look at individual variants that were called as true positives, false positives and false negatives.
The truvari command used was:

```
truvari.py -s 300 -S 270 -b HG002_SVs_Tier1_v0.6.DEL.vcf.gz -c $lumpy_vcf
-o eval-no-support --passonly --pctsim=0  -r 20 --giabreport -f  $fasta
--no-ref --includebed HG002_SVs_Tier1_v0.6.bed -O 0.6
```

To demonstrate the utility of *duphold* on duplication calls, we downloaded the sniffles[15] SV calls from http://labshare.cshl.edu/shares/schatzlab/www-data/fsedlaze/Sniffles/GiaB/all_reads.fa.giab_h002_ngmlr-0.2.3_mapped.bam.sniffles1kb_auto_noalts.vcf.gz and annotated DUP calls in that VCF with *duphold* using the same Illumina HG002 cram file as above.

To evaluate the scaling on realistic sites, we used duphold to annotate the same HG002 file, but on the 68,818 variants from the 1000 Genomes SV calls at: ftp://ftp.1000genomes.ebi.ac.uk/vol1/ftp/phase3/integrated_sv_map/ALL.wgs.mergedSV.v8.20130502.svs.genotypes.vcf.gz . We limited those calls to the variants that could be genotyped by svtyper (excluding insertions). We then randomly chose 100, 1000, 10K, 20K, 35K and 50K variants and ran svtyper and duphold on each set. We also ran duphold with 3 threads to evaluate the benefit of parallelization.

We downloaded the HG002 SNP/Indel calls from: ftp://ftp-trace.ncbi.nlm.nih.gov/giab/ftp/release/AshkenazimTrio/HG002_NA24385_son/latest/GRCh37/

Conclusions

Duphold enables rapid annotation of existing structural variant calls with sequence depth information that facilitates the distinction between high and low confidence deletions and duplications. Using the Genome in a Bottle truth set, we have shown that we can exclude nearly 64% of false positives SV predictions while retaining over 98% of true positive variants using a simple filter on a *duphold* annotated VCF. Given the minimal additional runtime of as few as 25 minutes for a 30X genome, this is a substantial improvement for the overall accuracy of SV callsets.

Availability of supporting source code and requirements

Project name: duphold
Project home page: https://github.com/brentp/duphold

Operating system(s): binary available for linux (can be built on OSX and windows)
Programming language: nim
Other requirements: htslib.so >= 1.8
License: MIT

Declarations

List of abbreviations

GiaB - genome in a bottle
SNP - single nucleotide polymorphism
SV - structural variant
VCF - variant call format

Consent for publication

Not applicable

Competing Interests

The author(s) declare that they have no competing interests

Funding

B. Pedersen and A. Quinlan were supported by the US National Institutes of Health National grants from the National Human Genome Research Institute (R01HG006693 and R01HG009141), the National Institute of General Medical Sciences (R01GM124355), and the National Cancer Institute (U24CA209999).

Author's contributions

BSP designed and wrote the software, performed the analyses and co-wrote the paper. ARQ co-wrote the paper.

References

1. Layer, R. M., Chiang, C., Quinlan, A. R. & Hall, I. M. LUMPY: a probabilistic framework for structural variant discovery. *Genome Biol.* **15,** R84 (2014).
2. Kronenberg, Z. N. *et al.* Wham: Identifying Structural Variants of Biological Consequence. *PLoS Comput. Biol.* **11,** e1004572 (2015).
3. Rausch, T. *et al.* DELLY: structural variant discovery by integrated paired-end and split-read analysis. *Bioinformatics* **28,** i333–i339 (2012).

4. Chen, K. *et al.* BreakDancer: an algorithm for high-resolution mapping of genomic structural variation. *Nat. Methods* **6,** 677–681 (2009).
5. Chen, X. *et al.* Manta: rapid detection of structural variants and indels for germline and cancer sequencing applications. *Bioinformatics* **32,** 1220–1222 (2016).
6. Belyeu, J. R. *et al.* SV-plaudit: A cloud-based framework for manually curating thousands of structural variants. *Gigascience* **7,** (2018).
7. Pedersen, B. S. & Quinlan, A. R. hts-nim: scripting high-performance genomic analyses. *Bioinformatics* **34,** 3387–3389 (2018).
8. Danecek, P. *et al.* The variant call format and VCFtools. *Bioinformatics* **27,** 2156–2158 (2011).
9. Li, H. *et al.* The Sequence Alignment/Map format and SAMtools. *Bioinformatics* **25,** 2078–2079 (2009).
10. Pedersen, B. S. & Quinlan, A. R. Mosdepth: quick coverage calculation for genomes and exomes. *Bioinformatics* **34,** 867–868 (2018).
11. Chiang, C. *et al.* SpeedSeq: ultra-fast personal genome analysis and interpretation. *Nat. Methods* **12,** 966–968 (2015).
12. Zook, J. M. *et al.* Extensive sequencing of seven human genomes to characterize benchmark reference materials. *Sci Data* **3,** 160025 (2016).
13. Li, H. Aligning sequence reads, clone sequences and assembly contigs with BWA-MEM. *arXiv [q-bio.GN]* (2013).
14. Faust, G. G. & Hall, I. M. SAMBLASTER: fast duplicate marking and structural variant read extraction. *Bioinformatics* **30,** 2503–2505 (2014).
15. Sedlazeck, F. J. *et al.* Accurate detection of complex structural variations using single-molecule sequencing. *Nat. Methods* **15,** 461–468 (2018).
