# Supplementary material for: Duphold: scalable, depth-based annotation and curation of high-confidence structural variant calls
Source: Gigascience. 2019 Apr 24;8(4):giz040. doi: 10.1093/gigascience/giz040 (PMC6479422; doi:10.1093/gigascience/giz040)
Supplement: GIGA-D-18-00471_Revision_2.pdf [file giz040_giga-d-18-00471_revision_2.pdf]

GigaScience

duphold: scalable, depth-based annotation and curation of high-confidence structural variant calls

--Manuscript Draft--

**Manuscript Number:**	GIGA-D-18-00471R2
**Full Title:**	duphold: scalable, depth-based annotation and curation of high-confidence structural variant calls
**Article Type:**	Technical Note
**Funding Information:**	National Human Genome Research Institute (R01HG006693) — Dr Aaron R Quinlan<br>National Human Genome Research Institute (R01HG009141) — Dr Aaron R Quinlan<br>National Institute of General Medical Sciences (R01GM124355) — Dr Aaron R Quinlan<br>National Cancer Institute (U24CA209999) — Dr Aaron R Quinlan
**Abstract:**	Most structural variant detection methods use clusters of discordant read-pair and split-read alignments to identify variants, yet do not integrate depth of sequence coverage as an additional means to support or refute putative events. Here, we present duphold, as a new method to efficiently annotate structural variant calls with sequence depth information that can add (or remove) confidence to SV that are predicted to affect copy number. Duphold indicates not only the change in depth across the event, but also the presence of a rapid change in depth relative to the regions surrounding the breakpoints. It uses a unique algorithm that allows the run time to be nearly independent of the number of variants. This performance is important for large, jointly-called projects with many samples, each of which must be evaluated at thousands of sites. We show that filtering on duphold annotations can greatly improve the specificity of structural variant calls. Duphold can annotate structural variant predictions made from both short-read and long-read sequencing datasets. It is available under the MIT license at: https://github.com/brentp/duphold.
**Corresponding Author:**	Brent Pedersen<br>University of Utah Hospital<br>UNITED STATES
**Corresponding Author Secondary Information:**	
**Corresponding Author's Institution:**	University of Utah Hospital
**Corresponding Author's Secondary Institution:**	
**First Author:**	Brent Pedersen
**First Author Secondary Information:**	
**Order of Authors:**	Brent Pedersen<br>Aaron R Quinlan, Phd
**Order of Authors Secondary Information:**	
**Response to Reviewers:**	> one question regarding the new results: is there an explanation why AUC of homozygous duplication (0.73) lower than it of heterozygous dup (0.74)?<br><br>This is likely due to th method we used to "create" duplications from GiaB insertion calls. when we create a DUP, we check that the purported INS maps next to the reported position. So, single-copy calls are likely to be very good and a bit more strict.

	That is, we know that if we called it a DUP, there is at least 1 copy of homology that can be found with illumina reads. It is not guaranteed for what GiaB calls as homozygous (1/1) INS since it could be that only a single copy can be found with illumina reads and not the expected 2 copies.<br>It is also likely affected by the smaller numbers of homozygous DUP (INS) calls.
Additional Information:	
Question	Response
Are you submitting this manuscript to a special series or article collection?	No
**Experimental design and statistics**<br><br>Full details of the experimental design and statistical methods used should be given in the Methods section, as detailed in our Minimum Standards Reporting Checklist. Information essential to interpreting the data presented should be made available in the figure legends.<br><br>Have you included all the information requested in your manuscript?	Yes
**Resources**<br><br>A description of all resources used, including antibodies, cell lines, animals and software tools, with enough information to allow them to be uniquely identified, should be included in the Methods section. Authors are strongly encouraged to cite Research Resource Identifiers (RRIDs) for antibodies, model organisms and tools, where possible.<br><br>Have you included the information requested as detailed in our Minimum Standards Reporting Checklist?	Yes
**Availability of data and materials**<br><br>All datasets and code on which the conclusions of the paper rely must be either included in your submission or deposited in publicly available repositories (where available and ethically appropriate), referencing such data using	Yes

a unique identifier in the references and in the “Availability of Data and Materials” section of your manuscript.<br><br>Have you have met the above requirement as detailed in our Minimum Standards Reporting Checklist?	

Powered by Editorial Manager® and ProduXion Manager® from Aries Systems Corporation

Manuscript
Click here to access/download;Manuscript;duphold manuscript.docx
Click here to view linked References

duphold: scalable, depth-based annotation and curation of high-confidence structural variant calls.

Brent S. Pedersen[1,3]* and Aaron R. Quinlan[1,2,3]*
1 Department of Human Genetics, University of Utah. Salt Lake City, UT
2 Department of Biomedical Informatics, University of Utah. Salt Lake City, UT
3 USTAR Center for Genetic Discovery, University of Utah. Salt Lake City, UT

* to whom correspondence should be addressed

ORCID IDs:
Brent S. Pedersen: 0000-0003-1786-2216; Aaron R. Quinlan: 0000-0003-1756-0859

Abstract

Most structural variant detection methods use clusters of discordant read-pair and split-read alignments to identify variants, yet do not integrate depth of sequence coverage as an additional means to support or refute putative events. Here, we present *duphold,* as a new method to efficiently annotate structural variant calls with sequence depth information that can add (or remove) confidence to SV that are predicted to affect copy number. Duphold indicates not only the change in depth across the event, but also the presence of a rapid change in depth relative to the regions surrounding the breakpoints. It uses a unique algorithm that allows the run time to be nearly independent of the number of variants. This performance is important for large, jointly-called projects with many samples, each of which must be evaluated at thousands of sites. We show that filtering on *duphold* annotations can greatly improve the specificity of structural variant calls. Duphold can annotate structural variant predictions made from both short-read and long-read sequencing datasets. It is available under the MIT license at: https://github.com/brentp/duphold.

Keywords

Structural-Variation
Genomics
Algorithm

Findings

Motivation

Structural variants (SV) are a broad class of genetic variation including duplications, deletions, inversions, insertions, and translocations. SVs are known to be more difficult to detect with high accuracy than single-nucleotide and insertion-deletion variants. As such, the false positive rate can be high. The most commonly used structural variant callers[1–5] use two types of sequence alignments to discover structural variation: paired-end reads having an unusual orientation or insert size (so called "discordant pairs"), and split-reads, where the sequence is aligned to different parts of the genome. These methods work well and while some make use of coverage information at the break-points, they do not directly integrate the aligned sequence depth within and around an event to detect or filter structural variant calls. This is an important limitation, since, for example, we expect a true hemizygous

deletion to exhibit 50% of the sequence coverage of flanking diploid regions. Based on our experience in evaluating the veracity of thousands of candidate SVs with SVPlaudit[6], we noted two consistent patterns that distinguished confident deletion and duplication calls from apparent false positives. First, events without an obvious reduction or increase in coverage are much less likely to appear as “real” events to the human eye. Second, events with a rapid change in depth at (or near) the breakpoints are more plausible. Obvious false positive calls lack either, or both, of those signals. We therefore developed *duphold* to enforce the observations we made through manual inspection and rapidly annotate SV calls in order to prioritize high-quality variant predictions.

Implementation

duphold uses hts-nim[7] to quickly extract coverage information from a BAM or CRAM file into an array using the methodology described in mosdepth[10]. Once in array format, it can be queried very rapidly. The depth profiles are used to quickly annotate a VCF[8] file of structural variants with coverage calculated from a BAM or CRAM file of alignments. Briefly, *duphold* operates on each chromosome sequentially; it allocates an (int16) array whose size is the length of the current chromosome (this array uses about 500 megabytes of memory for the 249 megabase human chromosome 1), iterates over each read in a BAM or CRAM for that chromosome, and increments any bases where an aligned read (or segment of a read) starts and decrements any bases where an aligned read (or part of a read) ends. A segment of a read is defined by the SAM[9] CIGAR operations. Once *duphold* has processed all segments for all alignments in a chromosome, it performs a cumulative sum which results in a per-base coverage value in the array. A 64 bit integer is used to track the actual depth but the depth stored on the arrays is capped at at the maximum value for a 16 bit integer (32767) to prevent integer overflow. This algorithm is fully detailed in the mosdepth manuscript.[10] Once the coverage array is filled, all remaining steps are independent of the number of alignments. Owing to the speed of in-memory array operations, subsequent depth calculations are nearly independent of the number of variants annotated in the VCF file.

For each structural variant, *duphold* annotates the VCF sample format field of the variant with both the change in depth relative to the surrounding 1,000 bases on either side of the event, and the fold-change in coverage in the event relative to other regions in the genome with similar GC-content. We have evaluated different flanking distances and 1,000 is sufficient to achieve an accurate estimate of coverage, but small enough to avoid commonly unsequenced regions or gaps in coverage. In order to compare the coverage observed for each variant with genomic bins of similar GC-content, duphold calculates the GC-content in each non-overlapping, 250-base window in the chromosome along with the median depth in that window. This requires 0.55 CPU-seconds for chromosome 1. These per-window depth and GC values are used as a distribution against which to compare incoming variants.

Once the depths and the GC-windows are calculated, *duphold*uses them to annotate structural variant calls in VCF format. For each variant, the GC-content is calculated for the genome interval defined by the variant, and the median depth inside the event is compared to the window values with a similar GC-content to calculate a fold-change value (DHBFC for Duphold Bin Fold-Change). *Duphold* then compares the median depth in the event to the median depth from the 1,000 bases on either side; this measure (named Duphold Flank Fold-Change, DHFFC) captures the change in depth one would observe by eye upon visual inspection. The depth fold-change values are added to the sample’s format information in the variant's VCF entry. Using the median for each metric makes the value more robust even

when the reported break-points are inexact, or shifted. *Duphold* is run on a single-sample at a time, but it has options to facilitate parallelization across samples. It can run on a 25X whole genome CRAM in <15 CPU-minutes and run-time will increase linearly with coverage.

Evaluation

Deletions

We evaluated *duphold* by annotating the lumpy[1] calls and svtyper[11] genotypes we produced for the HG002 sample sequenced by the Genome in a Bottle[12] (GiaB). We compared these to the GiaB truth-set of deletions for the same sample. We used the *duphold* annotations to filter to more stringent call sets and evaluate both precision and recall. Because *duphold* does not add any new variants, it can only improve precision, not recall.

Method	FDR	FN	FP	TP	Precision	Recall	F1-score
Unfiltered	0.053	276	83	1496	0.947	0.844	0.893
DHBFC < 0.7	0.018	298	27	1474	0.982	0.832	0.901
DHFFC < 0.7	0.021	289	32	1483	0.979	0.837	0.902

Table 1. Evaluating accuracy of deletion calls filtered by duphold annotations. We evaluated deletion calls from lumpy+svtyper using truvari.py (https://github.com/spiralgenetics/truvari) with the GiaB v0.6 truthset. Columns are FDR: false discovery rate, FN: false negatives, FP: false positive, TP: true-positive, precision, recall, and F1 score. DHBFC is an acronym for duphold bin fold-change which compares to regions (bins) of similar GC content. DHFFC is an acronym for duphold flank fold-change (with 1000 base flank). This shows that using either the DHBFC < 0.7 or DHFFC < 0.7 as a filtering criteria for deletions increases precision, removing 61% (1 - 32 / 83) of false positive calls while retaining more than 99% (1483 / 1496) of true positive calls in the case of using DHFFC.

The duphold depth annotations enable simple filters that reduce the number of false positives while retaining most true positives (**Table 1**). For example, requiring that the fold-change of the deletion relative to the 1000 bases flanking the deletion must be less than 0.7 (DHFFC < 0.7) removes 61% ((83 - 32 / 83)) of the false positive calls, while retaining 99% (1483 / 1496) of the true positive calls. The DHBFC metric measures the depth fold-change relative to bins with a similar GC-content, and performs similarly. Using more stringent filtering can further reduce the false positive rate at the expense of the recall. The information used in this filtering is independent of the values reported by lumpy and svtyper which do not look at sequence depth metrics.

We examined each of the false positive calls that remained after *duphold* filtering. These included a mixture of complex regions that had a loss of coverage, and some that looked like they could be real variants, but with minimal alignment support. We also visually inspected each of the 13 (i.e., 1496 - 1483) true positives that *duphold* marked as low confidence owing to a flank fold-change greater than 0.7 (DHFFC > 0.7). Most of these had a minimal change in coverage that did not meet our threshold and many looked like they did not have strong evidence for a call. We even noted one variant that looked like a duplication within a deletion, resulting in a copy-neutral event. While these highlight the limitations of a purely depth-based approach, we find that the more than 2-fold reduction in false positives in concert with a retention of 99% of true positives to be a convincing demonstration of

duphold's power to remove the abundant false positive SV prediction common to most analyses.

Duplications

Because *lumpy* called only a single duplication in HG002 that was not found in GiaB, we were not able to evaluate the performance of *duphold* on duplications using that approach. Since GiaB SV callset does not differentiate insertion events from duplications, we first classified any GiaB insertion as a duplication if the entirety of the reported insertion sequence was mapped by bwa-mem[13] with less than 5 mismatches to within 3 bases (start and end) of the variant. This resulted in 805 duplications for the truth-set.

In order to evaluate the specificity and sensitivity of duphold, we had to create homozygous reference variants. Specifically, for each heterozygous (0/1) or homozygous alternate (1/1) variant, we simulated a homozygous reference variant of the same size and type (e.g. for a heterozygous duplication, we simulated a homozygous reference duplication) and inserted it into the VCF. We limited the simulated variants to the high-confidence regions provided by GiaB and then retried any variant where more than 10% of the reference nucleotide sequence inside the simulated event was unknown ('N'). This approach provided a reasonable set of homozygous reference variants of a similar size distribution within the high-confidence GiaB regions.

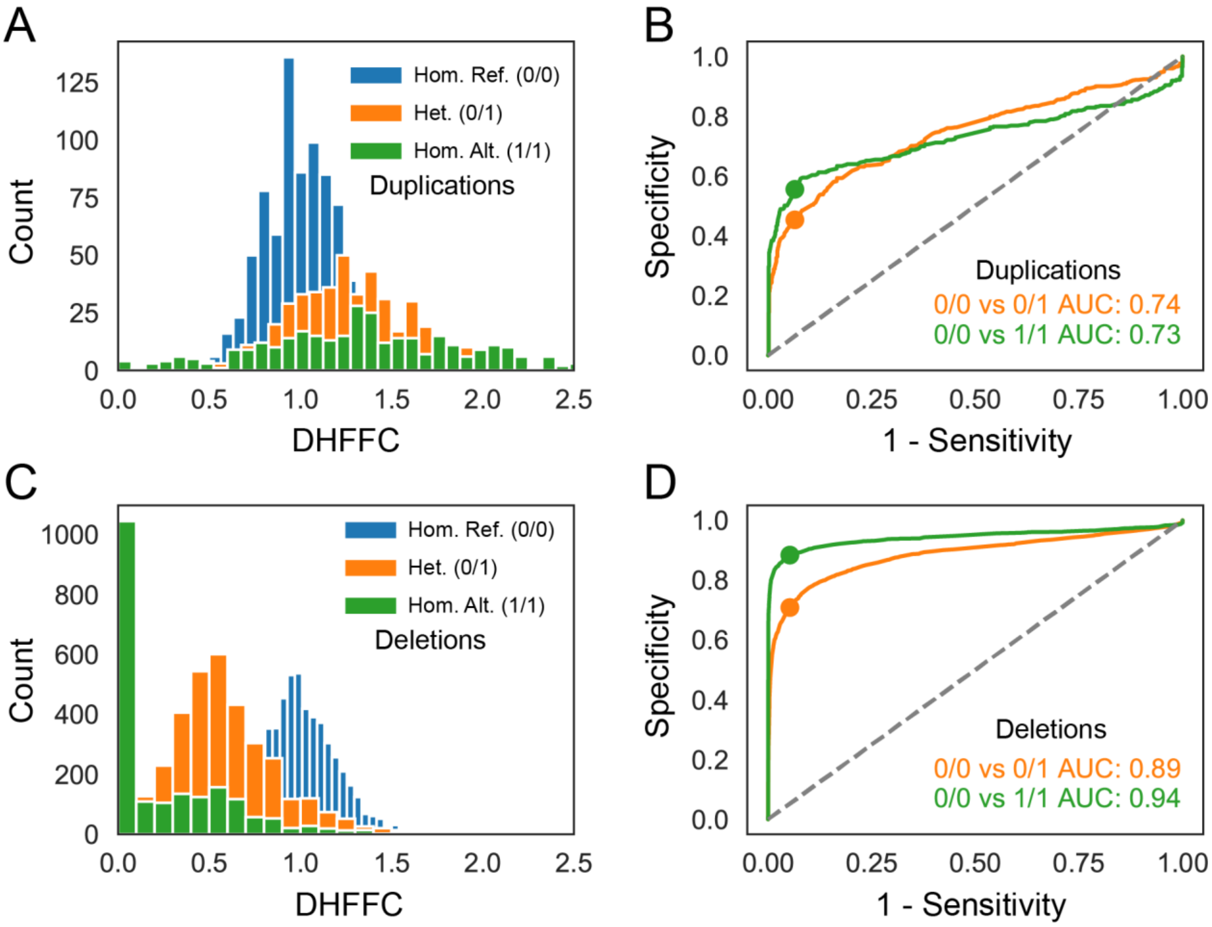

Figure 1. Evaluation of duphold on duplications and deletions of any size. We annotated 805 GiaB insertion calls as duplications and simulated homozygous-reference events of similar size in order to evaluate the specificity and sensitivity of *duphold*. We show the distribution of DHFFC (duphold flank fold-change) for each genotype (homozygous reference (0/0) is blue, heterozygous (0/1) is orange, and homozygous alternate (1/1) is

green), for both duplications (**A**) and deletions (**C**). We then used those distributions to create ROC-curves of (**B** and **D**) and calculate area-under the curves (AUC) that indicate the ability of *duphold* to differentiate 0/0 from 0/1 (orange) and 1/1 (green). The dots on the curves indicate a cutoff of 1.3 for duplications and 0.7 for deletions.

We evaluated the sensitivity and specificity of duphold using both the real and simulated deletions and duplications in Figure 1. While *duphold* is better able to differentiate deletions from random, copy-neutral locations, it still has an area under curve (AUC) of 0.74 for heterozygous duplications and 0.73 for homozygous duplications. The dots in the ROC curves show the sensitivity and specificity of *duphold* at a cutoff of 0.7 for deletions and 1.3 for duplications. The reduced performance on duplications relative to deletions is expected because a heterozygous deletion results in a 2-fold change in depth while a heterozygous duplication has only a 1.5-fold change. In addition, it could be that a subset of duplications in GiaB, which was created with a combination of technologies, cannot be detected with short-read illumina data. While the performance shown in Figure 1 reflects all event sizes, when deletions are restricted to those larger than 1kb, duphold achieves AUCs of 0.97 and 1.0 for heterozygous and homozygous alternate genotypes, respectively. At that size, the number of duplications is too low to properly evaluate, but we expect that larger events will enable *duphold* to more accurately evaluate the depth inside the event, and therefore further improve performance.

Scaling

We designed *duphold* with the expectation that it would be used on large datasets where both specificity and run time are critical. For this reason, we optimized it for situations where it would be used to evaluate many thousands of variants. In an effort to measure scaling performance, we compared the times of both svtyper and duphold on subsets of the thousand genomes phase 3 structural variants[14] (**Figure 1**). We note that we are not interested in the direct time comparison with svtyper, since svtyper does more work to genotype the variants. Instead, the relevant pattern is the trajectory in order to demonstrate how well *duphold* scales. Svtyper follows a linear increase in run time with the number of variants, while *duphold's* performance is nearly independent of the number of variants, using either 1 or 3 threads. This performance is driven by the fact that all of the alignment data is read into efficient data structures that can be queried thousands of times a second. This strategy incurs a large initial cost to construct the data structure, and therefore makes *duphold* less efficient for small variant sets. We have intentionally chosen to optimize for larger variant sets, since this context is where efficiency is most important.

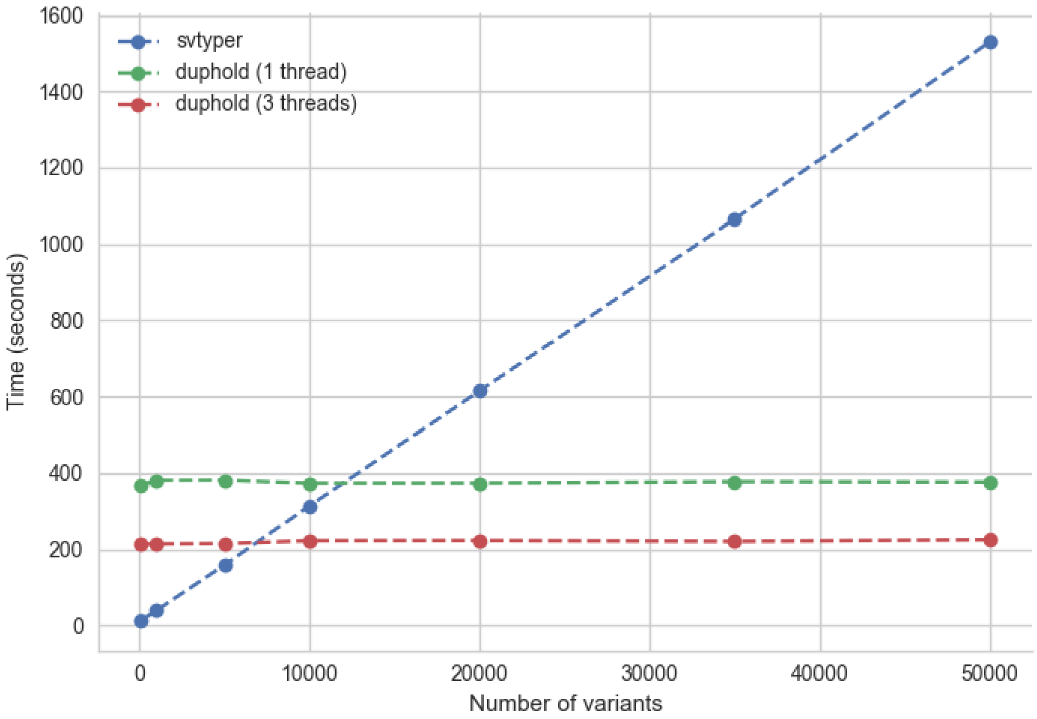

Figure 1. ***Duphold scalability.*** The time to annotate (or genotype) for duphold and svtyper is shown (y-axis) as a function of the number of variants tested (x-axis). While svtyper (blue) exhibits a linear increase in type with the number of variants, *duphold* is relatively independent of the number of variants. There is an initial cost that makes the *duphold* strategy less efficient for few (less than about 10,000) variants but it scales well to annotating thousands of variants as we expect for large cohorts.

Methods

To evaluate the ability of *duphold* to prioritize structural variant calls, we used data from the Genome in a Bottle project for sample HG002. We downloaded all fastqs from: ftp://ftp-trace.ncbi.nlm.nih.gov/giab/ftp/data/AshkenazimTrio/HG002_NA24385_son/NIST_HiSeq_HG002_Homogeneity-10953946/HG002_HiSeq300x_fastq/140528_D00360_0018_AH8VC6ADXX/ ,aligned with bwa-mem[15], and marked duplicates with samblaster[16] to generate a CRAM file with ~25X median sequence coverage. We used the GiaB SV calls and tier 1 regions from ftp://ftptrace.ncbi.nlm.nih.gov/giab/ftp/data/AshkenazimTrio/analysis/NIST_SVs_Integration_v0.6/ as our truth-set. We ran lumpy (LUMPY, RRID:SCR_003253)[1] and svtyper[11] via smoove (https://github.com/brentp/smoove) to create and genotype structural variant calls. We evaluated the precision and recall before and after applying various filtering on the duphold annotated variants using truvari (https://github.com/spiralgenetics/truvari). Specifically, we used a modified version of truvari here: https://github.com/brentp/truvari/tree/no-filter-as-pass to allow '.' filters to be consider as PASS. We used samplot (https://github.com/ryanlayer/samplot) to look at individual variants that were called as true positives, false positives and false negatives.
The truvari command used was:

```
truvari.py -s 300 -S 270 -b HG002_SVs_Tier1_v0.6.DEL.vcf.gz -c $lumpy_vcf
-o eval-no-support --passonly --pctsim=0  -r 20 --giabreport -f  $fasta --
no-ref --includebed HG002_SVs_Tier1_v0.6.bed -O 0.6
```

To demonstrate the utility of *duphold* on duplication calls, we annotated some GiaB insertion calls as duplications, using https://github.com/brentp/duphold/blob/paper/master/giab_ins_to_dup.nim and then simulated homozgyous reference calls of the same size and genomic distribution as the existing calls using https://github.com/brentp/duphold/blob/master/paper/insert_regions.nim.

To evaluate the scaling on realistic sites, we used duphold to annotate the same HG002 file, but on the 68,818 variants from the 1000 Genomes SV calls at: ftp://ftp.1000genomes.ebi.ac.uk/vol1/ftp/phase3/integrated_sv_map/ALL.wgs.mergedSV.v8.20130502.svs.genotypes.vcf.gz . We limited those calls to the variants that could be genotyped by svtyper (excluding insertions). We then randomly chose 100, 1000, 10K, 20K, 35K and 50K variants and ran svtyper and duphold on each set. We also ran duphold with 3 threads to evaluate the benefit of parallelization.

We downloaded the HG002 SNP/Indel calls from: ftp://ftp-trace.ncbi.nlm.nih.gov/giab/ftp/release/AshkenazimTrio/HG002_NA24385_son/latest/GRCh37/

Conclusions

Duphold enables rapid annotation of existing structural variant calls with sequence depth information that facilitates the distinction between high and low confidence deletions and duplications. Using the Genome in a Bottle truth set, we have shown that we can exclude nearly 61% of false positives SV predictions while retaining over 99% of true positive variants using a simple filter on a *duphold* annotated VCF. Given the minimal additional runtime of as few as 25 minutes for a 30X genome, this is a substantial improvement for the overall accuracy of SV callsets.

Availability of supporting source code and requirements

Project name: duphold
Project home page: https://github.com/brentp/duphold
Operating system(s): binary available for linux (can be built on OSX and windows)
Programming language: nim
Other requirements: htslib.so >= 1.8
License: MIT
RRID: SCR_016938

Availability of supporting data

An archival copy of the code is available in the *GigaScience* GigaDB repository[17].

Declarations

List of abbreviations

AUC – Area Under the Curves
GiaB - genome in a bottle

SNP - single nucleotide polymorphism
SV - structural variant
VCF - variant call format

Consent for publication

Not applicable

Competing Interests

The author(s) declare that they have no competing interests

Funding

B. Pedersen and A. Quinlan were supported by the US National Institutes of Health National grants from the National Human Genome Research Institute (R01HG006693 and R01HG009141), the National Institute of General Medical Sciences (R01GM124355), and the National Cancer Institute (U24CA209999).

Author's contributions

BSP designed and wrote the software, performed the analyses and co-wrote the manuscript. ARQ co-wrote the manuscript.

References

1. Layer, R. M., Chiang, C., Quinlan, A. R. & Hall, I. M. LUMPY: a probabilistic framework for structural variant discovery. *Genome Biol.* **15**, R84 (2014).
2. Kronenberg, Z. N. *et al.* Wham: Identifying Structural Variants of Biological Consequence. *PLoS Comput. Biol.* **11**, e1004572 (2015).
3. Rausch, T. *et al.* DELLY: structural variant discovery by integrated paired-end and split-read analysis. *Bioinformatics* **28**, i333–i339 (2012).
4. Chen, K. *et al.* BreakDancer: an algorithm for high-resolution mapping of genomic structural variation. *Nat. Methods* **6**, 677–681 (2009).
5. Chen, X. *et al.* Manta: rapid detection of structural variants and indels for germline and cancer sequencing applications. *Bioinformatics* **32**, 1220–1222 (2016).
6. Belyeu, J. R. *et al.* SV-plaudit: A cloud-based framework for manually curating thousands of structural variants. *Gigascience* Jul 1;7(**7**). doi: 10.1093/gigascience/giy064. (2018)

7. Pedersen, B. S. & Quinlan, A. R. hts-nim: scripting high-performance genomic analyses. *Bioinformatics* **34**, 3387–3389 (2018).

8. Danecek, P. *et al.* The variant call format and VCFtools. *Bioinformatics* **27**, 2156–2158 (2011).

9. Li, H. *et al.* The Sequence Alignment/Map format and SAMtools. *Bioinformatics* **25**, 2078–2079 (2009).

10. Pedersen, B. S. & Quinlan, A. R. Mosdepth: quick coverage calculation for genomes and exomes. *Bioinformatics* **34**, 867–868 (2018).

11. Chiang, C. *et al.* SpeedSeq: ultra-fast personal genome analysis and interpretation. *Nat. Methods* **12**, 966–968 (2015).

12. Zook, J. M. *et al.* Extensive sequencing of seven human genomes to characterize benchmark reference materials. *Sci Data* **3**, 160025 (2016).

13. Website.

14. Sudmant, P. H. *et al.* An integrated map of structural variation in 2,504 human genomes. *Nature* **526**, 75–81 (2015).

15. Li, H. Aligning sequence reads, clone sequences and assembly contigs with BWA-MEM. *arXiv [q-bio.GN]* (2013).

16. Faust, G. G. & Hall, I. M. SAMBLASTER: fast duplicate marking and structural variant read extraction. *Bioinformatics* **30**, 2503–2505 (2014).

17. Pedersen BS; Quinlan AR (2019): Supporting data for "duphold: scalable, depth-based annotation and curation of high-confidence structural variant calls" GigaScience Database. http://dx.doi.org/10.5524/100579
