# Supplementary material for: Duphold: scalable, depth-based annotation and curation of high-confidence structural variant calls
Source: Gigascience. 2019 Apr 24;8(4):giz040. doi: 10.1093/gigascience/giz040 (PMC6479422; doi:10.1093/gigascience/giz040)
Supplement: Supplemental Files [file giz040_supplemental_files.zip › Additional file 1.docx]

**Table S1 Statistics of sequencing data on Chinese hickory and pecan using Illumina HiSeq X-ten and PacBio RSII**

| Illumina HiSeq X-ten | |  |  |  |  |  |  |
| --- | --- | --- | --- | --- | --- | --- | --- |
| *Cca* |  |  |  |  |  |  |  |
| Insert size(bp) | Raw Reads | Raw bases(bp) | Clean_base(bp) | Error_rate(%) | Q20(%) | Q30(%) | GC_content(%) |
| 250 | 159695988 | 47908796400 | 41831101800 | 0.015 | 98.03 | 95.58 | 38.39 |
| 500 | 170894990 | 51268497000 | 47924349000 | 0.025 | 95.36 | 90.85 | 37.23 |
| 2000 | 63832258 | 19149677400 | 14873292600 | 0.015 | 97.58 | 94.81 | 36.54 |
| 5000 | 56167313 | 16850193900 | 10754447700 | 0.025 | 96.3 | 91.95 | 36.49 |
| 10000 | 38202833 | 11460849900 | 4541998200 | 0.025 | 97.34 | 93.6 | 36.31 |
| 20000 | 35029842 | 10508952600 | 3397876500 | 0.015 | 98.26 | 96.31 | 36.96 |
|  |  |  |  |  |  |  |  |
| *Cil* |  |  |  |  |  |  |  |
| Insert size(bp) | Raw Reads | Raw bases(bp) | Clean_base(bp) | Error_rate(%) | Q20(%) | Q30(%) | GC_content(%) |
| 250 | 192681852 | 57804555600 | 50874063900 | 0.02 | 97.11 | 93.38 | 40.19 |
| 500 | 184055349 | 48281125350 | 45653165150 | 0.025 | 95.04 | 90.19 | 38.84 |
| 2000 | 62472514 | 18741754200 | 16608308700 | 0.0425 | 93.59 | 86.72 | 37.3 |
| 5000 | 63572394 | 19071718200 | 14270402100 | 0.0375 | 94.31 | 88.1 | 37.09 |
| 10000 | 26924674 | 8077402200 | 4508661600 | 0.0325 | 94.74 | 89.16 | 36.8 |
| 20000 | 31495740 | 9448722000 | 3375238500 | 0.0275 | 95.54 | 90.55 | 36.71 |
|  |  |  |  |  |  |  |  |
| PacBio RSII | |  |  |  |  |  |  |
| *Cca* |  |  |  |  |  |  |  |
| Insert size(bp) | Raw Reads | Raw bases(bp) | Clean_base(bp) | reads quality | |  |  |
| 20000 | 1963854 | 21719667950 | 21682000290 | 0.84 |  |  |  |
|  |  |  |  |  |  |  |  |
| *Cil* |  |  |  |  |  |  |  |
| Insert size(bp) | Raw Reads | Raw bases(bp) | Clean_base(bp) | reads quality | |  |  |
| 20000 | 1869620 | 25802999003 | 25752717123 | 0.84 |  |  |  |

**Table S2 Material informations of *Carya* and *Juglans* used for whole genome sequencing and re-sequencing.**

| Samples collected from China (* materials for whole genome sequencing) | | | | | | | | | | | | | | | | |  |
| --- | --- | --- | --- | --- | --- | --- | --- | --- | --- | --- | --- | --- | --- | --- | --- | --- | --- |
| Species | Plant ID | | Collection location | | Latitude | | Longitude | | | Altitude | | Sample collector | | Sampling date | Sequencing_depth | |  |
| ***C. cathayensis*** | **ZAFU-1** | | **Lin'an, Zhejiang** | | **N30°15'18.1"** | | **E119°43'44.3"** | | | **40** | | **Youjun Huang** | | **11-Apr-2016*** | **248** | |  |
| ***C. illinoinensis*** | **‘Pawnee’** | | **Lin'an, Zhejiang** | | **N30°15'18.2"** | | **E119°43'46.2"** | | | **40** | | **Zhengjia Wang** | | **15-Aug-2015*** | **288** | |  |
| ***C. cathayensis*** | **ZAFU-1** | | **Lin'an, Zhejiang** | | **N30°15'18.1"** | | **E119°43'44.3"** | | | **40** | | **Youjun Huang** | | **28-Jul-2013** | **26.93** | |  |
| ***C. illinoinensis*** | **‘Pawnee’** | | **Lin'an, Zhejiang** | | **N30°15'18.2"** | | **E119°43'46.2"** | | | **40** | | **Zhengjia Wang** | | **28-Jul-2013** | **14.94** | |  |
| *C. hunanensis* | H2-1 | | Longtan, Jingzhou, Hunan | | N30°15'41.3" | | E119°43'30.8" | | | 153 | | Chuanmei Xu | | 28-Jul-13 | 12.14 | |  |
| *C. tonkinensis* | Y3 | | Songyun, Jiasha, Gejiu, Honghe, Yunnan | | N23°22'63.2" | | E102°57'58.2" | | | 1125 | | Chuanmei Xu | | 28-Jul-13 | 7.15 | |  |
| *C. kweichowensis* | Gz1 | | Muzai, Anlong, Guizhou | | N25°03'46.9" | | E105°21'42.1" | | | 1208 | | Zhengjia Wang | | 01-Aug-13 | 7.88 | |  |
| *C. dabieshanensis* | DBS5 | | Xiantao, Guanmiao, Jinzhai, Anhui | | N31°29'21" | | E115°23'31" | | | 496 | | Chuanmei Xu | | 28-Jul-13 | 6.24 | |  |
| *J. regia* | Jr | | Luoning, Henan | | N34°22'25" | | E111°23'6" | | | 1437 | | Dong Pei | | 01-May-14 | 8.34 | |  |
| *J. sigillata* | Js | | Yangbi, Dali, Yunnan | | N29°39'23" | | E100°1'25" | | | 1850 | | Dong Pei | | 01-May-14 | 14.57 | |  |
| Samples sent by USDA-ARS National Clonal Germplasm Repository (NCGR) | | | | | | | | | | | | | | | | | |
| Species | Plant ID | Sample ID | | Plant ID | | Orchard | | Row | Tree | | Date | | Sequencing_depth | | | originated at Latitude | |
| *C. floridana* | Cf607 | LJ14-165 | | 88-FLA-FL-1.1 | | CSS | | 22 | 5 | | 25-Sep-14 | | 6.13 | | | 29.183600 | |
| *C. aquatica* | Ca02 | LJ14-150 | | 02-AQU-LA-CL2 | | CSPA | | 2 | 1 | | 2-Oct-14 | | 9.42 | | | 32.533337 | |
| *C. cordiformis* | Cc02 | LJ14-151 | | 02-COR-LA-BF1 | | CSPA | | 4 | 1 | | 2-Oct-14 | | 11.19 | | | 32.182696 | |
| *C. palmeri* | Cp10 | LJ14-153 | | Ring | | Scrnhse | | 10 |  | | 25-Sep-14 | | 7.39 | | | 25.345716 | |
| *C. myristiciformis* | Cm92 | LJ14-154 | | 92-MYR-TX-1G | | CSV | | 23 | 31 | | 2-Oct-14 | | 8.12 | | | 31.627559 | |
| *C. laciniosa* | Cl07 | LJ14-156 | | 07-LAC-KS-1 | | CSS | | 23 | 1 | | 25-Sep-14 | | 7.45 | | | 37.032557 | |
| *C. ovata* | Co01 | LJ14-158 | | Yoder #1 | | CSS | | 20 | 1 | | 2-Oct-14 | | 8.41 | | | 40.270903 | |
| *C. tomentosa* | Ct92 | LJ14-160 | | 92-TOM-LA-2G | | CSV | | 24 | 31 | | 2-Oct-14 | | 8.75 | | | 32.337925 | |
| *C. texana* | Ct10 | LJ14-162 | | 10-TEX-WC-3 | | CSS | | 20.5 | 2 | | 2-Oct-14 | | 10.44 | | | 30.48116667 | |
| *C. glabra* | Cg01 | LJ14-166 | | 01-GLA-LA-3.26 | | CSS | | 18 | 2 | | 2-Oct-14 | | 13.12 | | | 32.183960 | |

**Table S3 Statistics of input data for genome assemblies of pecan and Chinese hickory**

| Platform | Library insert size | Total raw data (Gb) | | Total clean data (Gb) | | Reads length (bp) | Sequence coverage of raw data(X) | | Sequence coverage of clean data(X) | |
| --- | --- | --- | --- | --- | --- | --- | --- | --- | --- | --- |
|  |  | *Cca* | *Cil* | *Cca* | *Cil* |  | *Cca* | *Cil* | *Cca* | *Cil* |
| Illumina Hiseq X-Ten | 250 bp | 47.91 | 57.80 | 41.83 | 50.87 | 150 (PE) | 66.42 | 88.96 | 57.99 | 78.29 |
|  | 500 bp | 51.27 | 48.28 | 47.92 | 45.65 |  | 71.08 | 74.31 | 66.43 | 70.26 |
|  | 2 kb | 19.15 | 18.74 | 14.87 | 16.61 | 150 (MP) | 26.55 | 28.84 | 20.61 | 25.56 |
|  | 5 kb | 16.85 | 19.07 | 10.75 | 14.27 |  | 23.36 | 29.35 | 14.90 | 21.96 |
|  | 10 kb | 11.46 | 8.08 | 4.54 | 4.51 |  | 15.89 | 12.44 | 6.29 | 6.94 |
|  | 20 kb | 10.51 | 9.45 | 3.40 | 3.38 |  | 14.57 | 14.54 | 4.71 | 5.20 |
| Pacbio RSII | 20 kb | 21.72 | 25.80 | 21.68 | 25.75 | - | 30.11 | 39.71 | 30.06 | 39.63 |
| Total | - | 178.87 | 187.22 | 144.99 | 161.04 | - | 247.97 | 288.20 | 200.99 | 247.84 |

**Table S4 Summary of the final genome assemblies of pecan and Chinese hickory*.***

| Sample ID | length | | | | number | | | |
| --- | --- | --- | --- | --- | --- | --- | --- | --- |
|  | Contig**(bp) | | Scaffold(bp) | | Contig** | | Scaffold | |
|  | *Cca* | *Cil* | *Cca* | *Cil* | *Cca* | *Cil* | *Cca* | *Cil* |
| Total | 698,719,178 | 636,748,445 | 706,434,463 | 651,309,696 | 53,110 | 61,935 | 40,425 | 43,503 |
| Max | 1,107,680 | 500,198 | 4,951,520 | 4,915,448 | - | - | - | - |
| Number>=2kb | - | - | - | - | 15,789 | 17,542 | 5,449 | 3,860 |
| N50 | 101,582 | 77,227 | 1,223,633 | 1,067,362 | 1,879 | 2,388 | 174 | 188 |
| N60 | 76,484 | 58,993 | 966,109 | 806,381 | 2,668 | 3,336 | 239 | 259 |
| N70 | 53,918 | 42,766 | 712,681 | 623,320 | 3,752 | 4,606 | 324 | 352 |
| N80 | 34,410 | 27,753 | 420,315 | 434,909 | 5,366 | 6,432 | 451 | 474 |
| N90 | 14,970 | 11,671 | 137,394 | 210,676 | 8,355 | 9,823 | 732 | 682 |

**Table S5 Genome survey of pecan (*Cil*) and Chinese hickory (*Cca*).**

| Species | Total base (Gb) | K-mer | K-mer number | K-mer depth | Genome size (Mb) | Revised genome size (Mb) | Heterozygous ratio (%) | Repeat ratio (%) |
| --- | --- | --- | --- | --- | --- | --- | --- | --- |
| *Cca* | 47.91 | 17 | 37,369,117,608 | 51 | 732.73 | 721.33 | 0.77 | 57.30 |
| *Cil* | 76.85 | 17 | 633,320,055,362 | 95 | 666.53 | 649.75 | 1.46 | 48.20 |

**Table S6 Coverage statistics of pecan (*Cil*) and Chinese hickory (*Cca*) genomes.**

|  |  | Percentage | |
| --- | --- | --- | --- |
|  |  | *Cca* | *Cil* |
| Reads | Mapping rate (%) | 99.09% | 96.81% |
| Genome | Coverage (%) | 99.14% | 95.28% |
|  | Coverage at least 4X (%) | 98.41% | 93.55% |
|  | Coverage at least 10X (%) | 96.80% | 91.58% |
|  | Coverage at least 20X (%) | 91.59% | 88.15% |
|  | Average sequencing depth | 56.05X | 100.06X |

Average sequence depth: The average depth of each base on the genome that is covered by reads;

Coverage**：**The proportion of genomes that were covered by reads.

**Table S7 Assessment of the gene coverage rate using CEGMA.**

| species | complete | | complete + partial | |
| --- | --- | --- | --- | --- |
|  | # Prots | % completeness | # Prots | % completeness |
| *Cca* | 239 | 96.37 | 234 | 94.35 |
| *Cil* | 235 | 94.76 | 231 | 93.15 |

**Table S8 Assessment of the gene coverage rate using BUSCO.**

| Species | Size (Mbp) | BUSCO notation assessment results |
| --- | --- | --- |
| *Cca* | 702.02 | C:95%[D:23%], F:1.4%, M:3.1%, n:956 |
| *Cil* | 648.71 | C:94%[D:23%], F:1.4%, M:3.6%, n:956 |

Size: genome size;

BUSCO notation: C: Complete Single-Copy BUSCOs; D: Complete Duplicated BUSCOs; F: Fragmented BUSCOs; M: Missing BUSCOs; n: Total BUSCO groups searched.

**Table S9 Summary of repeat sequences in pecan and Chinese hickory genomes.**

| Type | *Cca* | | *Cil* | |
| --- | --- | --- | --- | --- |
|  | Length (bp) | Percent (%) | Length (bp) | Percent (%) |
| Tandem repeats | 30,315,866 | 4.27 | 21,156,972 | 3.19 |
| TE repeats |  |  |  |  |
| DNA | 50,304,746 | 7.09 | 40,788,579 | 6.15 |
| LINE | 60,136,636 | 8.47 | 62,546,992 | 9.43 |
| SINE | 140,833 | 0.02 | 300,735 | 0.05 |
| LTR | 264,441,668 | 37.25 | 221,356,553 | 33.37 |
| Satellite | 939,414 | 0.13 | 388,276 | 0.06 |
| Simple repeat | 17,810,179 | 2.51 | 6,699,248 | 1.01 |
| Unknown* | 17,241,962 | 2.43 | 28,264,088 | 4.26 |
| Other** | 0 | 0 | 6,538 | 9.85E-04 |
| Total | 381,006,671 | 53.67 | 334,549,042 | 50.43 |

**Table S10 Summary of repetitive sequence in the assembled pecan and Chinese hickory genomes.**

| Class | Superfamily | *Cil* | | *Cca* | |
| --- | --- | --- | --- | --- | --- |
|  |  | Length (kb) | Percent (%) | Length (kb) | Percent (%) |
| Class I (Retro TE) | LTR/Copia | 102207.7 | 15.406 | 131646.9 | 18.544 |
|  | LTR/Gypsy | 99933.9 | 15.063 | 112965.1 | 15.912 |
|  | LTR/Caulimovirus | 4530.5 | 0.683 | 8255.9 | 1.163 |
|  | LTR/ERV1 | 301.5 | 0.045 | 266.4 | 0.038 |
|  | Other LTR | 30279.4 | 4.564 | 26465.9 | 3.728 |
|  | LINE/L1 | 62185.1 | 9.373 | 59679.0 | 8.406 |
|  | Other LINE | 888.4 | 0.134 | 1013.3 | 0.143 |
|  | SINE | 296.2 | 0.045 | 130.1 | 0.018 |
|  | Other SINE | 4.5 | 0.001 | 10.7 | 0.002 |
| Class II(DNA TE) | DNA/CMC | 12790.2 | 1.928 | 14495.9 | 2.042 |
|  | DNA/hAT | 11574.1 | 1.745 | 12611.3 | 1.776 |
|  | DNA/PIF | 3533.6 | 0.533 | 5486.3 | 0.773 |
|  | DNA/Helitron | 2991.4 | 0.451 | 4096.6 | 0.577 |
|  | DNA/MULE | 4715.9 | 0.711 | 3761.6 | 0.530 |
|  | Other DNA TE | 6492.7 | 0.979 | 10496.9 | 1.479 |
| No category (TE) |  | 28264.1 | 4.260 | 17242.0 | 2.429 |
| Tandem repeat | Satellite | 388.3 | 0.059 | 939.4 | 0.132 |
|  | Simple repeat | 6699.2 | 1.010 | 17810.2 | 2.509 |
| Total |  | 378076.9 | 50.4 | 427373.4 | 53.7 |

**Table S11 Summary of gene structure prediction in pecan and Chinese hickory genomes.**

| Species | Gene set | | Number | Average  CDS+intron length (bp) | Average CDS length (bp) | Average exons per gene | Average exon length (bp) | Average intron length (bp) |
| --- | --- | --- | --- | --- | --- | --- | --- | --- |
| Pecan | *De novo* | Augustus | 36885 | 3326.45 | 1017.71 | 4.28 | 237.79 | 703.90 |
|  |  | GlimmerHMM | 68000 | 7802.36 | 609.33 | 3.12 | 195.04 | 3386.34 |
|  |  | SNAP | 74731 | 5128.94 | 650.86 | 3.80 | 171.35 | 1600.21 |
|  |  | Geneid | 74870 | 4012.18 | 602.14 | 3.38 | 178.16 | 1432.90 |
|  |  | Genscan | 44228 | 8898.55 | 978.59 | 5.21 | 187.80 | 1880.89 |
|  | Homolog | *Cucumis sativus* | 39422 | 2882.10 | 1019.51 | 3.65 | 279.32 | 702.87 |
|  |  | *Citrullus lanatus* | 33677 | 2989.05 | 974.94 | 3.82 | 255.15 | 713.98 |
|  |  | *Prunus persica* | 35958 | 2714.46 | 1098.69 | 3.52 | 311.78 | 640.17 |
|  |  | *Malus domestica* | 30919 | 3002.40 | 1184.33 | 3.64 | 325.60 | 689.34 |
|  |  | *Vitis vinifera* | 34630 | 3136.08 | 1016.52 | 3.89 | 261.60 | 734.50 |
|  |  | *Glycine max* | 40287 | 2953.32 | 973.68 | 3.60 | 270.15 | 760.17 |
|  |  | *Eucalyptus grandis* | 28247 | 3419.87 | 1212.15 | 3.93 | 308.35 | 753.21 |
|  |  | *Arabidopsis thaliana* | 42980 | 2509.57 | 922.59 | 3.25 | 283.75 | 704.87 |
|  |  | *Populus trichocarpa* | 37848 | 2523.52 | 1004.51 | 3.38 | 297.57 | 639.39 |
|  |  | *Oryza sativa* | 40859 | 2201.72 | 981.38 | 2.93 | 334.56 | 631.21 |
|  | RNA-seq | Cufflinks* | 57126 | 6749.01 | 1977.25 | 5.99 | 330.20 | 956.64 |
|  |  | PASA | 62573 | 3623.67 | 996.77 | 4.61 | 216.15 | 727.39 |
|  | EVM | | 40744 | 3635.33 | 970.39 | 4.30 | 225.79 | 808.13 |
|  | PASA-update | | 40530 | 3585.01 | 979.89 | 4.30 | 227.93 | 789.65 |
|  | Final set | | 31075 | 4223.11 | 1142.70 | 4.96 | 230.28 | 777.45 |
| Chinese hickory | *De novo* | Augustus | 40049 | 3160.15 | 1010.6 | 4.15 | 243.43 | 682.06 |
|  |  | GlimmerHMM | 73920 | 7628.5 | 604.53 | 3.02 | 200.3 | 3480.51 |
|  |  | SNAP | 77556 | 5095.88 | 660.19 | 3.74 | 176.33 | 1616.45 |
|  |  | Geneid | 74689 | 4083.79 | 645.63 | 3.51 | 184.12 | 1371.65 |
|  |  | Genscan | 45739 | 9135.86 | 1023.01 | 5.32 | 192.33 | 1878.33 |
|  | Homolog | *Cucumis sativus* | 43523 | 2923.20 | 1034.77 | 3.46 | 299.42 | 768.93 |
|  |  | *Citrullus lanatus* | 36379 | 2957.74 | 950.91 | 3.68 | 258.60 | 749.62 |
|  |  | *Prunus persica* | 40973 | 2668.67 | 1119.52 | 3.33 | 336.24 | 665.02 |
|  |  | *Malus domestica* | 35810 | 2940.41 | 1219.89 | 3.32 | 366.93 | 740.13 |
|  |  | *Vitis vinifera* | 36910 | 3237.55 | 1019.31 | 3.76 | 270.86 | 802.76 |
|  |  | *Glycine max* | 44699 | 2990.41 | 976.09 | 3.44 | 283.96 | 826.39 |
|  |  | *Eucalyptus grandis* | 31043 | 3468.46 | 1231.75 | 3.72 | 331.09 | 822.24 |
|  |  | *Arabidopsis thaliana* | 46534 | 2509.35 | 926.37 | 3.14 | 295.05 | 739.80 |
|  |  | *Populus trichocarpa* | 42909 | 2400.48 | 996.76 | 3.18 | 313.90 | 645.25 |
|  |  | *Oryza sativa* | 45163 | 2181.63 | 1027.28 | 2.81 | 366.12 | 639.22 |
|  | RNA-seq | Cufflinks | 68784 | 7609.68 | 2348.63 | 6.50 | 361.07 | 955.74 |
|  |  | PASA | 91513 | 3667.97 | 977.51 | 4.56 | 214.49 | 756.29 |
|  | EVM | | 43597 | 3659.08 | 971.69 | 4.22 | 230.34 | 835.00 |
|  | PASA-update | | 43312 | 3618.53 | 980.60 | 4.21 | 232.73 | 820.92 |
|  | Final set | | 32907 | 4312.79 | 1146.21 | 4.89 | 234.17 | 813.02 |
|  |  |  |  |  |  |  |  |  |

**Table S12 Statistics of *de novo* assemblies using RNA-Seq data from leaf tissue of Chinese hickory by Trinity.**

| Total length (bp) | Total number | Number  (>=2000) | Average length (bp) | N50 length (bp) | N50 number | N90 length | N90 number |
| --- | --- | --- | --- | --- | --- | --- | --- |
| 141,177,704 | 159000 | 17,833 | 887 | 1559 | 27,705 | 343 | 105,823 |

**Table S13 Summary of gene structure prediction of pecan, Chinese hickory and reference genomes.**

| Species | Number | | Average CDS+intron length (bp) | | Average CDS length (bp) | Average exons per gene | Average exon length (bp) | Average intron length (bp) |
| --- | --- | --- | --- | --- | --- | --- | --- | --- |
| *C. illinoinensis* | | 31075 | | 4223.11 | 1142.70 | 4.96 | 230.28 | 777.45 |
| *C. cathayensis* | 32907 | | 4312.79 | | 1146.21 | 4.89 | 234.17 | 813.02 |
| *Cucumis sativus* | 18738 | | 3814.02 | | 1362.45 | 5.84 | 233.21 | 506.3 |
| *Citrullus lanatus* | 23440 | | 2770.04 | | 1109.68 | 4.58 | 242.51 | 464.34 |
| *Prunus persica* | 28087 | | 2417.28 | | 1203.99 | 4.86 | 247.55 | 314.03 |
| *Malus domestica* | 47821 | | 4775.74 | | 1209.53 | 4.88 | 248.05 | 920.02 |
| *Vitis vinifera* | 29927 | | 4728.63 | | 1095.81 | 4.75 | 230.72 | 968.88 |
| *Glycine max* | 54174 | | 3194.35 | | 1185.97 | 5.17 | 229.49 | 481.89 |
| *Eucalyptus grandis* | 35774 | | 3741.97 | | 1322.46 | 4.94 | 267.95 | 614.8 |
| *Arabidopsis thaliana* | 27416 | | 1870.34 | | 1218.4 | 5.13 | 237.58 | 157.91 |
| *Populus trichocarpa* | 41377 | | 2317.62 | | 1115.94 | 4.68 | 238.39 | 326.44 |
| *Oryza sativa* | 35679 | | 2165.58 | | 991.55 | 3.78 | 262.57 | 422.87 |

**Table S14 Statistics of gene function annotation in pecan and Chinese hickory genomes.**

| Database | | Annotated genes in *Cca* | | Annotated gnes in *Cil* | |
| --- | --- | --- | --- | --- | --- |
|  |  | Annotated Number | Annotated Percent (%) | Annotated Number | Annotated Percent (%) |
| NR | | 31100 | 94.5 | 29692 | 95.5 |
| Swiss-Prot | | 25547 | 77.6 | 24287 | 78.2 |
| KEGG | | 23933 | 72.7 | 22748 | 73.2 |
| InterPro | All | 26852 | 81.6 | 25576 | 82.3 |
|  | Pfam | 24936 | 75.8 | 23806 | 76.6 |
|  | GO | 18365 | 55.8 | 17437 | 56.1 |
| Annotated | | 31159 | 94.7 | 29737 | 95.7 |

**Table S15 Statistics of non-coding RNA in pecan and Chinese hickory genomes.**

| Type | | Copy (w*) | | | Average length (bp) | | Total length (bp) | | % of genome | |
| --- | --- | --- | --- | --- | --- | --- | --- | --- | --- | --- |
|  |  | *Cil* | | *Cca* | *Cil* | *Cca* | *Cil* | *Cca* | *Cil* | *Cca* |
| miRNA | | 378 | 373 | | 120.95 | 128.28 | 45719 | 47847 | 0.006891 | 0.006740 |
| tRNA | | 571 | 558 | | 74.80 | 74.92 | 42709 | 41804 | 0.006438 | 0.005888 |
| rRNA | **rRNA** | 198 | 362 | | 192.94 | 145.23 | 38202 | 52575 | 0.005758 | 0.007406 |
|  | 18S | 61 | 46 | | 374.61 | 366.37 | 22851 | 16853 | 0.003444 | 0.002374 |
|  | 28S | 29 | 34 | | 141.90 | 125.62 | 4115 | 4271 | 0.00062 | 0.000602 |
|  | 5.8S | 11 | 12 | | 151 | 141.58 | 1661 | 1699 | 0.00025 | 0.000239 |
|  | 5S | 97 | 270 | | 98.71 | 110.19 | 9575 | 29752 | 0.001443 | 0.004191 |
| snRNA | **snRNA** | 886 | 1191 | | 111.54 | 109.13 | 98826 | 129973 | 0.014896 | 0.018308 |
|  | CD-box | 633 | 961 | | 104.01 | 103.61 | 65840 | 99572 | 0.009924 | 0.014026 |
|  | HACA-box | 83 | 75 | | 125.71 | 126.12 | 10434 | 9459 | 0.001573 | 0.001332 |
|  | splicing | 169 | 154 | | 132.67 | 135.14 | 22422 | 20812 | 0.00338 | 0.002932 |

*w indicates annotation at genome-wide level.

**Table S16 The mapping rate of re-sequencing data from 16 species in *Carya* and two species in *Juglans* (out-group).**

|  | Species | Mapping rate (%) | Average depth |
| --- | --- | --- | --- |
| East Asian | *C. cathayensis* | 99.15 | 26.93 |
|  | *C. dabieshanensis* | 96.95 | 6.24 |
|  | *C. kweichowensis* | 87.50 | 7.88 |
|  | *C. hunanensis* | 83.80 | 12.14 |
|  | *C. tonkinensis* | 85.27 | 7.15 |
| East North America | *C. illinoinensis* | 72.32 | 14.94 |
|  | *C. palmeri* | 73.94 | 7.39 |
|  | *C. floridana* | 75.39 | 6.13 |
|  | *C. aquatica* | 77.21 | 9.42 |
|  | *C. cordiformis* | 77.69 | 11.19 |
|  | *C. myristiciformis* | 77.81 | 8.12 |
|  | *C. laciniosa* | 78.07 | 7.45 |
|  | *C. tomentosa* | 78.69 | 8.75 |
|  | *C. ovata* | 79.10 | 10.51 |
|  | *C. glabra* | 80.67 | 13.12 |
|  | *C. texana* | 80.74 | 10.44 |
| Out-group | *J. regia* | 34.90 | 8.34 |
|  | *J. sigillata* | 40.58 | 14.57 |

**Table 17 Statistics of synteny blocks within pecan and Chinese hikcory, and among pecan, Chinese hickory and walnut.**

| Species | Number of synteny blocks | Average collinear genes per block | Number of collinear genes in all blocks | Mean block length in currect species |
| --- | --- | --- | --- | --- |
| *C. cathayensis/C. cathayensis* | 343 | 31 | 10530 | 508,640 |
| *C. illinoinensis/C. illinoinensis* | 342 | 22 | 7682 | 274,742 |
| *C. cathayensis/C. illinoinensis* | 1252 | 30 | 37038 | 268,683 |
| *C. cathayensis/J. regia* | 1062 | 28 | 29980 | 257,521 |
| *C. illinoinensis/J. regia* | 1086 | 24 | 25848 | 191,808 |

**Table S18 Significantly enriched KEGG pathway of Chinese hickory-specific gene families.**

|  | Pathway | Adjusted P-value |
| --- | --- | --- |
| 1 | Ribosome | 0.000011 |
| 2 | Glycerophospholipid metabolism | 0.012859 |
| 3 | **Linoleic acid metabolism** | 0.031671 |

**Table S19 Significantly enriched KEGG pathways involved in the expanded gene families in pecan and Chinese hickory.**

|  | Pathway | AdjustedP-value |
| --- | --- | --- |
| **Pecan** |  |  |
| 1 | Citrate cycle (TCA cycle) | 0.002430 |
| 2 | 2-Oxocarboxylic acid metabolism | 0.002430 |
| 3 | **Peroxisome** | 0.002430 |
| 4 | **Glutathione metabolism** | 0.002998 |
| 5 | Phenylpropanoid biosynthesis | 0.014164 |
| 6 | **Plant-pathogen interaction** | 0.014636 |
| 7 | Ribosome | 0.040122 |
| 8 | Carbon metabolism | 0.040122 |
| 9 | Biosynthesis of amino acids | 0.041518 |
| **Chinese hickory** |  |  |
| 1 | Sesquiterpenoid and triterpenoid biosynthesis | 0.000000 |
| 2 | Limonene and pinene degradation | 0.000000 |
| 3 | Stilbenoid, diarylheptanoid and gingerol biosynthesis | 0.000000 |
| 4 | **Biosynthesis of secondary metabolites** | 0.000000 |
| 5 | **Flavonoid biosynthesis** | 0.000004 |
| 6 | Monoterpenoid biosynthesis | 0.000008 |
| 7 | Galactose metabolism | 0.000015 |

**Table S20 The statistical result of genes related to oil accumulation in pecan, Chinese hickory and other oil plants.**

|  | *Cca* | *Cil* | *Rco* | *Sin* | *Egu* | *Adu* | *Gma* | *Ath* |
| --- | --- | --- | --- | --- | --- | --- | --- | --- |
| alpha-CT | 3 | 3 | 2 | 3 | 3 | 2 | 4 | 1 |
| BC | 2 | 1 | 1 | 1 | 3 | 2 | 2 | 1 |
| BCCP | 4 | 4 | 2 | 2 | 2 | 4 | 5 | 2 |
| beta-CT | 1 | 1 | 0 | 0 | 0 | 0 | 0 | 1 |
| Heteromeric ACCase | 10 | 9 | 5 | 6 | 8 | 8 | 11 | 5 |
| Homomeric ACCase | 2 | 2 | 1 | 0 | 1 | 1 | 2 | 2 |
| FATA | 4 | 2 | 1 | 1 | 2 | 1 | 2 | 2 |
| FATB | 2 | 2 | 1 | 1 | 3 | 2 | 4 | 1 |
| SAD/DES1 | 0 | 0 | 0 | 0 | 0 | 0 | 0 | 1 |
| SAD/DES2 | 0 | 0 | 0 | 0 | 0 | 0 | 0 | 1 |
| SAD/DES3 | 0 | 0 | 0 | 0 | 0 | 0 | 0 | 1 |
| SAD/DES4 | 0 | 0 | 0 | 0 | 0 | 0 | 0 | 1 |
| SAD/DES5 | 1 | 1 | 3 | 2 | 2 | 0 | 0 | 1 |
| SAD/DES6 | 1 | 2 | 1 | 3 | 1 | 5 | 3 | 1 |
| SAD/FAB2 | 8 | 4 | 1 | 2 | 4 | 1 | 2 | 1 |
| SAD sum | 10 | 7 | 5 | 7 | 7 | 6 | 5 | 7 |
| AAS | 0 | 0 | 0 | 0 | 0 | 0 | 0 | 1 |
| ABCAT | 1 | 2 | 1 | 1 | 1 | 1 | 1 | 1 |
| ACC2 | 0 | 0 | 0 | 0 | 0 | 0 | 0 | 1 |
| ACP1 | 0 | 0 | 0 | 0 | 1 | 0 | 1 | 1 |
| ACP2 | 1 | 0 | 3 | 0 | 0 | 0 | 0 | 1 |
| ACP3 | 1 | 0 | 0 | 0 | 1 | 3 | 3 | 1 |
| ACP4 | 1 | 2 | 1 | 4 | 3 | 0 | 2 | 1 |
| alpha-PDH | 1 | 1 | 1 | 1 | 1 | 2 | 4 | 1 |
| beta-PDH | 2 | 2 | 1 | 4 | 2 | 2 | 4 | 2 |
| DHLAT/EMB3003(E2) | 1 | 1 | 1 | 1 | 2 | 1 | 2 | 1 |
| DHLAT/LTA2(E2) | 2 | 2 | 1 | 1 | 1 | 1 | 2 | 1 |
| ER/ENR1(MOD1) | 2 | 2 | 2 | 3 | 2 | 1 | 4 | 1 |
| HACPS | 1 | 1 | 1 | 1 | 1 | 1 | 1 | 1 |
| HAD | 2 | 2 | 1 | 1 | 2 | 2 | 4 | 2 |
| KAR | 6 | 3 | 3 | 5 | 5 | 6 | 7 | 5 |
| KASI | 3 | 3 | 3 | 3 | 4 | 1 | 5 | 1 |
| KASII | 2 | 3 | 1 | 3 | 3 | 2 | 3 | 1 |
| KASIII | 2 | 2 | 1 | 2 | 2 | 2 | 3 | 1 |
| LACS8 | 2 | 1 | 1 | 1 | 0 | 1 | 3 | 1 |
| LACS9 | 2 | 2 | 1 | 1 | 2 | 2 | 3 | 1 |
| LPD1(E3) | 0 | 0 | 1 | 1 | 0 | 1 | 2 | 1 |
| LPD2(E3) | 1 | 1 | 0 | 1 | 1 | 0 | 1 | 1 |
| LS | 4 | 2 | 1 | 1 | 1 | 1 | 2 | 1 |
| LT | 0 | 1 | 1 | 1 | 0 | 2 | 3 | 1 |
| MCMT | 2 | 2 | 1 | 1 | 1 | 2 | 3 | 1 |
| PII | 4 | 2 | 2 | 1 | 1 | 1 | 3 | 1 |
| TGD1 | 1 | 1 | 1 | 2 | 1 | 1 | 2 | 1 |
| WRI1 | 4 | 5 | 3 | 5 | 6 | 1 | 6 | 1 |
| WRI3 | 4 | 4 | 2 | 2 | 0 | 2 | 6 | 1 |
| WRI4 | 0 | 0 | 0 | 0 | 1 | 0 | 0 | 1 |
| Sum | 100 | 85 | 58 | 75 | 81 | 71 | 120 | 65 |

Note: *Cca, Carya cathayensis; Cil, Carya illinoinensis; Rco, Ricinus communis; Sin, Sesamum indicum; Egu, Elaeis guineensis; Adu, Arachis duranensis; Gma, Glycine max; Ath, Arabidopsis thaliana.*

**Table S21 The transcriptional level of genes related to oil accumulation in pecan.**

| Gene ID | Gene name | PEY1 | | PEY2 | | PEY3 |
| --- | --- | --- | --- | --- | --- | --- |
| CIL0304S0005 | ABCAT | 0.226793 | 0.039199 | | 0.014151 | |
| CIL0914S0003 | ABCAT | 3.845013 | 0.709296 | | 1.918755 | |
| CIL1495S0014 | ABI3 | 112.8813 | 97.01738 | | 80.7977 | |
| CIL0134S0018 | ABI4 | 34.25574 | 9.95286 | | 11.35525 | |
| CIL1082S0124 | ACP4 | 9.57938 | 7.939115 | | 3.308222 | |
| CIL1216S0091 | ACP4 | 8.391239 | 3.103671 | | 2.520044 | |
| CIL0368S0003 | AH/DAD1 | 0 | 0 | | 0 | |
| CIL0897S0094 | AH/DAD1 | 0 | 0 | | 0 | |
| CIL1066S0018 | AP1AGL7 | 0.099646 | 0.314783 | | 0.652869 | |
| CIL1182S0010 | AP1AGL7 | 7.258126 | 1.944449 | | 0.981259 | |
| CIL1071S0039 | ARF | 8.977312 | 14.84221 | | 3.711986 | |
| CIL1087S0019 | ARF | 11.42619 | 6.039619 | | 6.472726 | |
| CIL1354S0026 | ARF | 29.92783 | 7.232502 | | 10.81671 | |
| CIL1615S0023 | ARF | 12.00893 | 3.905515 | | 2.792219 | |
| CIL0992S0011 | AtCLO1 | 160.5405 | 1408.437 | | 1187.387 | |
| CIL1615S0020 | BC | 186.7802 | 259.2225 | | 44.94706 | |
| CIL0013S0013 | BCCP1 | 25.33409 | 69.20277 | | 37.06538 | |
| CIL1461S0002 | BCCP1 | 20.45078 | 24.11541 | | 2.492616 | |
| CIL0094S0006 | BCCP2 | 85.46591 | 233.2366 | | 63.81549 | |
| CIL1518S0020 | BCCP2 | 34.97992 | 19.76537 | | 2.069687 | |
| CIL0001S0015 | CALO | 10.12972 | 3.764875 | | 26.37129 | |
| CIL0951S0139 | CALO | 18.57183 | 11.24554 | | 12.83244 | |
| CIL0893S0256 | CCT1 | 29.64499 | 22.10531 | | 29.98885 | |
| CIL0921S0045 | CCT1 | 103.5996 | 73.84651 | | 83.62896 | |
| CIL1420S0022 | CCT1 | 24.55274 | 18.22155 | | 23.55489 | |
| CIL1353S0007 | CDP-DAGS/CDS4 | 21.39686 | 14.18397 | | 3.587368 | |
| CIL0974S0085 | CJD1 | 29.57077 | 35.38109 | | 20.89466 | |
| CIL0937S0060 | CK | 16.20335 | 33.51267 | | 60.46702 | |
| CIL0987S0097 | CK | 0.383384 | 0.102743 | | 0.819709 | |
| CIL1066S0024 | CRC | 0 | 0 | | 0 | |
| CIL0982S0092 | CTS/pxa1 | 14.75573 | 7.263645 | | 14.19722 | |
| CIL0982S0093 | CTS/pxa1 | 20.31744 | 10.69998 | | 20.87524 | |
| CIL1253S0039 | CTS/pxa1 | 13.69177 | 10.68565 | | 14.96493 | |
| CIL0899S0176 | DGAT1 | 2.511857 | 0.616291 | | 1.585382 | |
| CIL1211S0036 | DGAT1 | 14.23264 | 22.62073 | | 23.78332 | |
| CIL1268S0007 | DGAT2 | 12.67463 | 8.294283 | | 11.63537 | |
| CIL1063S0015 | DGAT3 | 25.78069 | 18.99566 | | 40.45559 | |
| CIL1006S0096 | DGDGS/DGD1 | 16.77168 | 25.08785 | | 65.9611 | |
| CIL1142S0011 | DGDGS/DGD2 | 28.85124 | 26.70957 | | 28.22185 | |
| CIL0482S0001 | DHLAT/EMB3003(E2) | 83.72783 | 158.9674 | | 2.987572 | |
| CIL1321S0009 | DHLAT/LTA2(E2) | 63.53936 | 55.16699 | | 0.811696 | |
| CIL1524S0008 | DHLAT/LTA2(E2) | 53.59684 | 98.82551 | | 3.51743 | |
| CIL1113S0002 | ER/ENR1(MOD1) | 108.7793 | 119.9199 | | 8.831766 | |
| CIL1221S0019 | ER/ENR1(MOD1) | 139.0906 | 383.2714 | | 10.02683 | |
| CIL0987S0038 | FAD2 | 165.5989 | 13.35837 | | 12.99791 | |
| CIL1047S0089 | FAD2 | 0.168505 | 0 | | 0.006631 | |
| CIL1507S0011 | FAD2 | 217.0412 | 570.9813 | | 299.6433 | |
| CIL1417S0095 | FAD4 | 0.477222 | 1.352849 | | 0.055804 | |
| CIL1119S0061 | FAD6 | 10.53277 | 18.71704 | | 4.627752 | |
| CIL0953S0038 | FAD7 | 29.29956 | 82.71801 | | 8.479377 | |
| CIL1021S0079 | FAD8 | 3.100424 | 10.79244 | | 1.162841 | |
| CIL1264S0069 | FAD8 | 14.72632 | 2.926187 | | 2.414824 | |
| CIL1604S0003 | FAD8 | 2.497686 | 4.716203 | | 0.438259 | |
| CIL0992S0068 | FATA | 39.66035 | 74.1581 | | 3.44647 | |
| CIL1445S0018 | FATA | 28.61154 | 102.0638 | | 32.49146 | |
| CIL0895S0249 | FATB | 31.09252 | 16.08411 | | 4.719604 | |
| CIL1173S0086 | FATB | 67.87758 | 37.4614 | | 33.82252 | |
| CIL0948S0064 | FUS3 | 21.69372 | 28.4623 | | 1.467357 | |
| CIL1051S0072 | GGGT/SFR2 | 6.918385 | 9.537449 | | 31.70588 | |
| CIL0173S0002 | GLK1 | 0 | 0 | | 0.109315 | |
| CIL0993S0078 | GLK1 | 5.557915 | 1.703347 | | 1.973296 | |
| CIL1090S0079 | GPAT/ATS1/ACT1 | 12.76029 | 6.380265 | | 5.488751 | |
| CIL1157S0009 | GPAT/ATS1/ACT1 | 4.986763 | 1.971307 | | 1.49298 | |
| CIL0156S0025 | GPAT9 | 9.834648 | 5.702736 | | 1.610997 | |
| CIL0240S0006 | GPAT9 | 17.98083 | 17.91082 | | 17.04957 | |
| CIL0922S0157 | GPDHP | 4.476581 | 120.3142 | | 2.761142 | |
| CIL1145S0139 | GPDHP | 13.96543 | 35.41974 | | 2.611042 | |
| CIL0919S0138 | GPDHc1 | 7.49925 | 24.14687 | | 8.530298 | |
| CIL1596S0004 | GPDHc1 | 22.25841 | 18.59001 | | 1.666813 | |
| CIL1093S0066 | HACPS | 7.936436 | 5.314111 | | 3.49517 | |
| CIL0424S0003 | HAD | 29.32687 | 37.18913 | | 0.748662 | |
| CIL0982S0066 | HAD | 96.09875 | 128.995 | | 11.44012 | |
| CIL1209S0007 | HSD1 | 2.13984 | 13.06338 | | 173.3221 | |
| CIL0320S0012 | HSI2/VAL1 | 21.91184 | 33.42821 | | 35.20491 | |
| CIL1226S0065 | HSI2/VAL1 | 24.26952 | 22.99666 | | 14.0973 | |
| CIL1319S0023 | HSI2/VAL1 | 15.48739 | 9.589898 | | 12.01927 | |
| CIL1324S0052 | HSL1/VAL2 | 18.4842 | 14.61386 | | 41.12297 | |
| CIL1484S0020 | HSL1/VAL2 | 23.54311 | 10.14974 | | 15.49415 | |
| CIL0156S0004 | KAR | 0.958451 | 1.483251 | | 0.054077 | |
| CIL1029S0041 | KAR | 18.37206 | 14.38147 | | 8.128111 | |
| CIL1476S0021 | KAR | 84.34881 | 167.3054 | | 15.42387 | |
| CIL1002S0061 | KASI | 7.136686 | 3.418349 | | 0.670737 | |
| CIL1113S0057 | KASI | 83.87567 | 127.8232 | | 11.15487 | |
| CIL1393S0019 | KASI | 78.90127 | 187.8805 | | 8.83023 | |
| CIL1087S0023 | KASII | 45.91621 | 160.5395 | | 52.58094 | |
| CIL1295S0065 | KASII | 0.242076 | 0.464931 | | 0.158781 | |
| CIL1354S0030 | KASII | 21.54978 | 25.3212 | | 2.234754 | |
| CIL0098S0006 | KASIII | 31.13063 | 46.08325 | | 30.51936 | |
| CIL0923S0064 | KASIII | 20.78272 | 33.24333 | | 5.531348 | |
| CIL1155S0013 | LACS8 | 31.81923 | 26.48404 | | 90.44292 | |
| CIL1112S0009 | LACS9 | 53.859 | 208.721 | | 30.50192 | |
| CIL1587S0008 | LACS9 | 52.61703 | 63.22974 | | 7.78325 | |
| CIL1099S0061 | LCAT-PLA | 19.50708 | 13.2543 | | 13.38054 | |
| CIL1500S0014 | LEC1 | 28.17227 | 4.53256 | | 0 | |
| CIL0893S0390 | LEC2 | 3.593436 | 0.318817 | | 0 | |
| CIL0011S0014 | LPAAT1 | 18.07822 | 11.11318 | | 10.68876 | |
| CIL1420S0007 | LPAAT1 | 4.954655 | 3.702068 | | 3.624975 | |
| CIL0957S0120 | LPAAT2 | 0 | 0 | | 0.037503 | |
| CIL1047S0130 | LPAAT2 | 75.63707 | 37.51282 | | 30.89116 | |
| CIL1262S0035 | LPAAT2 | 0 | 0 | | 0 | |
| CIL0102S0019 | LPAAT3 | 0 | 0 | | 0 | |
| CIL1193S0064 | LPAAT4 | 1.389558 | 0.825348 | | 5.823597 | |
| CIL1334S0001 | LPAAT4 | 0.120423 | 0 | | 0.498571 | |
| CIL1078S0039 | LPAAT5 | 14.87604 | 11.78013 | | 6.371712 | |
| CIL0235S0020 | LPCAT1 | 87.03491 | 146.6727 | | 27.94394 | |
| CIL0923S0139 | LPCAT1 | 10.21792 | 2.638876 | | 2.666879 | |
| CIL0914S0027 | LPD2(E3) | 119.0002 | 207.9605 | | 13.19119 | |
| CIL1066S0025 | LS | 0 | 0 | | 0.038701 | |
| CIL1182S0016 | LS | 13.57134 | 16.34103 | | 3.499681 | |
| CIL1053S0041 | LT | 6.838044 | 3.51874 | | 2.536406 | |
| CIL0972S0111 | MCMT | 64.18015 | 80.78952 | | 4.481262 | |
| CIL1114S0058 | MCMT | 4.187054 | 0.387001 | | 0.010407 | |
| CIL1031S0128 | MGDGS/MGD1 | 7.123769 | 9.450983 | | 8.492254 | |
| CIL1300S0072 | MGDGS/MGD1 | 13.436 | 13.07642 | | 6.366253 | |
| CIL1003S0138 | MGDGS/MGD2 | 2.285821 | 1.136 | | 0.568643 | |
| CIL1614S0011 | MGDGS/MGD2 | 5.203302 | 3.589833 | | 2.810064 | |
| CIL0212S0015 | OBO | 0 | 0 | | 0 | |
| CIL1212S0020 | OBO | 407.4145 | 1153.495 | | 669.752 | |
| CIL0009S0029 | OLE1 | 1820.767 | 7510.745 | | 3478.503 | |
| CIL0940S0119 | OLE1 | 1332.164 | 5864.392 | | 7014.403 | |
| CIL0252S0009 | OLE4 | 49.55938 | 583.2436 | | 183.6854 | |
| CIL0922S0190 | OLE4 | 728.4224 | 7753.279 | | 1957.221 | |
| CIL0938S0150 | OLE4 | 151.9076 | 3555.508 | | 2473.62 | |
| CIL1494S0045 | OLE4 | 949.881 | 4561.382 | | 822.6033 | |
| CIL0945S0104 | PDAT1 | 15.25196 | 15.24717 | | 5.461675 | |
| CIL1131S0029 | PDAT1 | 32.79469 | 4.959693 | | 7.540049 | |
| CIL1167S0044 | PDAT1 | 10.79261 | 9.121216 | | 6.242172 | |
| CIL0277S0001 | PDAT2 | 19.18608 | 114.3252 | | 37.00165 | |
| CIL0957S0163 | PDCT/ROD1 | 39.48449 | 119.2657 | | 5.32819 | |
| CIL0923S0269 | PGPS/PGP1 | 0.077027 | 0 | | 0 | |
| CIL0993S0027 | PGPS/PGP1 | 0.031687 | 0 | | 0 | |
| CIL1003S0055 | PGPS/PGP1 | 10.6049 | 6.173643 | | 4.181469 | |
| CIL1546S0045 | PGPS/PGP1 | 10.16807 | 9.785024 | | 4.943351 | |
| CIL0962S0015 | PII | 190.8316 | 194.105 | | 26.90947 | |
| CIL1075S0076 | PII | 178.342 | 352.9206 | | 24.89944 | |
| CIL1138S0077 | PKL | 54.15763 | 30.39769 | | 25.32198 | |
| CIL1154S0071 | PP/AtLPP3 | 20.77063 | 20.61226 | | 28.32807 | |
| CIL0923S0055 | PP/LPP-beta | 12.21875 | 3.829637 | | 5.250319 | |
| CIL0987S0005 | PP/LPP-delta | 17.52843 | 37.44584 | | 1.514546 | |
| CIL1057S0065 | PP/LPP-delta | 28.65717 | 24.04384 | | 10.48409 | |
| CIL0919S0008 | PP/LPP-epsilon2 | 2.093223 | 0.528086 | | 0.701257 | |
| CIL0929S0023 | PP/LPP-epsilon2 | 3.200717 | 2.090255 | | 0.370593 | |
| CIL0940S0009 | PP/LPP-gamma | 28.0889 | 13.1207 | | 5.412716 | |
| CIL0989S0054 | PP/LPP-gamma | 0 | 0.018618 | | 0.058175 | |
| CIL1396S0025 | PP/LPP1 | 0.371773 | 0.448504 | | 0.330017 | |
| CIL1619S0015 | PP/LPP1 | 9.417373 | 5.232113 | | 4.862513 | |
| CIL1118S0001 | PP/LPP2 | 0 | 0 | | 0 | |
| CIL1156S0031 | PP/LPP2 | 0.247742 | 0 | | 0.368153 | |
| CIL1396S0024 | PP/LPP2 | 6.212698 | 1.763328 | | 0.102385 | |
| CIL1619S0016 | PP/LPP2 | 13.68806 | 1.561538 | | 0.06116 | |
| CIL1012S0126 | PP/PAH2 | 15.73734 | 18.73527 | | 9.69356 | |
| CIL0001S0039 | PPIase | 52.16091 | 36.51284 | | 32.57075 | |
| CIL1216S0010 | PPIase | 69.80768 | 45.82526 | | 39.30164 | |
| CIL1286S0004 | PPIase | 32.29441 | 15.62721 | | 4.243561 | |
| CIL1409S0005 | PPIase | 0.521689 | 0.266657 | | 0.38961 | |
| CIL0373S0007 | SAD/DES5 | 11.41612 | 8.424834 | | 1.46546 | |
| CIL1230S0065 | SAD/DES6 | 54.35279 | 137.9002 | | 1.747415 | |
| CIL1297S0040 | SAD/DES6 | 407.3488 | 1230.485 | | 11.11538 | |
| CIL0126S0001 | SAD/FAB2 | 0.238052 | 0.076302 | | 0.168631 | |
| CIL0436S0014 | SAD/FAB2 | 72.87955 | 243.6039 | | 13.7981 | |
| CIL0962S0137 | SAD/FAB2 | 135.1489 | 195.1153 | | 80.34135 | |
| CIL1153S0024 | SAD/FAB2 | 0.735966 | 0.084689 | | 0.172967 | |
| CIL0996S0008 | SLS/SQD2 | 19.72292 | 3.253912 | | 1.991791 | |
| CIL1056S0024 | SLS/SQD2 | 6.950023 | 2.732109 | | 5.216879 | |
| CIL1506S0008 | SQS/SQD1 | 21.89302 | 46.67088 | | 22.01246 | |
| CIL1305S0048 | STERO | 25.75367 | 373.0985 | | 650.2684 | |
| CIL0345S0007 | TAGL-Like | 11.63203 | 4.940574 | | 1.695874 | |
| CIL0899S0100 | TAGL-Like | 0 | 0 | | 0 | |
| CIL0899S0097 | TAGL-Like | 0 | 0 | | 0 | |
| CIL0899S0098 | TAGL-Like | 0 | 0 | | 0 | |
| CIL0899S0099 | TAGL-Like | 0 | 0 | | 0 | |
| CIL0902S0140 | TAGL-Like | 19.11194 | 61.64548 | | 4.220168 | |
| CIL0917S0160 | TAGL-Like | 0.515018 | 0 | | 0.055228 | |
| CIL1025S0126 | TAGL-Like | 12.21617 | 9.358806 | | 17.83546 | |
| CIL1037S0058 | TAGL-Like | 2.732621 | 0.180241 | | 0.249911 | |
| CIL1037S0059 | TAGL-Like | 1.721487 | 0.244709 | | 0.162595 | |
| CIL1219S0035 | TAGL-Like | 23.30481 | 16.59351 | | 12.51932 | |
| CIL1300S0006 | TAGL-Like | 16.71442 | 25.00041 | | 12.61657 | |
| CIL1463S0025 | TAGL-Like/LIP1 | 25.08116 | 13.45188 | | 23.19723 | |
| CIL1122S0072 | TAGL-Like/MPL1 | 0 | 0.021644 | | 0.082659 | |
| CIL1495S0004 | TAGL-Like/MPL1 | 0 | 0 | | 0 | |
| CIL1325S0088 | TAGL-Like/SAG101 | 3.888136 | 0.989609 | | 0.508139 | |
| CIL0128S0007 | TAGL-Like/SDP1 | 8.337812 | 6.206533 | | 40.64693 | |
| CIL1009S0026 | TAGL-Like/SDP1 | 6.284613 | 6.560713 | | 13.71378 | |
| CIL0984S0017 | TGD1 | 38.40541 | 22.99229 | | 21.36929 | |
| CIL0151S0005 | TOC159 | 5.696373 | 3.171762 | | 2.613097 | |
| CIL1141S0015 | TOC159 | 19.10076 | 16.1109 | | 43.29604 | |
| CIL1127S0080 | TT2 | 1.344862 | 0.493839 | | 0.542016 | |
| CIL1333S0011 | TT2 | 0 | 0 | | 0 | |
| CIL0184S0008 | TT8/BHLH42 | 1.526721 | 1.957976 | | 1.001935 | |
| CIL0945S0059 | TT8/BHLH42 | 3.278071 | 0.660527 | | 0.346454 | |
| CIL1047S0041 | WRI1 | 47.91822 | 104.9576 | | 0.351185 | |
| CIL1076S0026 | WRI1 | 0.511114 | 0.146969 | | 0.043703 | |
| CIL1187S0078 | WRI1 | 66.11222 | 105.3369 | | 4.674679 | |
| CIL1277S0042 | WRI1 | 0 | 0.140539 | | 0.022186 | |
| CIL1325S0035 | WRI1 | 0.71943 | 1.710463 | | 0.198873 | |
| CIL0929S0104 | WRI3 | 0.082146 | 0.011357 | | 0.035488 | |
| CIL0979S0110 | WRI3 | 0 | 0 | | 0.04769 | |
| CIL1282S0027 | WRI3 | 0.127233 | 0.127056 | | 0 | |
| CIL1503S0031 | WRI3 | 0.032613 | 0 | | 0.080858 | |
| CIL0978S0098 | alpha-CT | 2.061057 | 0.744985 | | 8.707791 | |
| CIL1020S0079 | alpha-CT | 79.12843 | 177.7769 | | 18.01151 | |
| CIL1056S0033 | alpha-CT | 93.01131 | 161.158 | | 22.14449 | |
| CIL0389S0012 | alpha-PDH | 215.1332 | 387.5307 | | 37.81635 | |
| CIL1284S0002 | beta-CT | 8.666621 | 49.91766 | | 5.61281 | |
| CIL1229S0009 | beta-PDH | 147.2901 | 406.2199 | | 9.852519 | |
| CIL1280S0020 | beta-PDH | 0 | 0.08368 | | 0 | |

**Table S22 The transcriptional level of genes related to oil accumulation in Chinese hickory.**

| Gene ID | Gene name | HEY1 | HEY2 | HEY3 |
| --- | --- | --- | --- | --- |
| CCA1552S0016 | ABCAT | 2.593319937 | 0.092991871 | 0.528816375 |
| CCA0686S0034 | ABI3 | 108.2924773 | 83.90177765 | 92.74184818 |
| CCA1066S0037 | ABI3 | 81.66988122 | 53.40796412 | 50.73938425 |
| CCA0676S0065 | ABI4 | 58.98031439 | 21.86437993 | 39.56228916 |
| CCA0754S0127 | ACP2 | 0 | 0 | 0 |
| CCA0505S0339 | ACP3 | 7.614470511 | 4.073557897 | 2.397887637 |
| CCA0526S0093 | ACP4 | 56.54883871 | 65.05427729 | 50.62903937 |
| CCA0869S0033 | AH/DAD1 | 0 | 0 | 0 |
| CCA1741S0002 | AH/DAD1 | 0 | 0 | 0 |
| CCA0513S0137 | AP1AGL7 | 0.178117868 | 0.362316691 | 0.127432128 |
| CCA0852S0025 | AP1AGL7 | 0.061733221 | 0.092258462 | 0.007361039 |
| CCA0924S0033 | AP1AGL7 | 0.015992064 | 0 | 0 |
| CCA0691S0116 | ARF | 3.155732535 | 1.903160096 | 1.940605755 |
| CCA0977S0049 | ARF | 23.02707629 | 16.49853851 | 16.94597825 |
| CCA1225S0022 | ARF | 15.00400347 | 11.96698262 | 12.63582928 |
| CCA1353S0021 | ARF | 19.72795688 | 1.285519481 | 2.515171264 |
| CCA0691S0118 | BC | 1.627008275 | 1.335760943 | 0.458455529 |
| CCA1353S0018 | BC | 150.6891042 | 94.80500291 | 59.75834234 |
| CCA0529S0104 | BCCP1 | 30.30130929 | 38.43425296 | 18.68339337 |
| CCA0595S0033 | BCCP1 | 63.70560498 | 40.7966269 | 14.88320356 |
| CCA0649S0065 | BCCP2 | 312.0373137 | 260.6139221 | 99.38680488 |
| CCA0906S0099 | BCCP2 | 71.7311585 | 64.39441335 | 33.24806143 |
| CCA0601S0075 | CALO | 15.48441813 | 10.31891197 | 9.148965705 |
| CCA0899S0091 | CALO | 10.51501215 | 4.086280186 | 15.52087167 |
| CCA0849S0013 | CALO/AtCLO1 | 436.3510079 | 1059.241323 | 1728.751731 |
| CCA0539S0050 | CCT1 | 6.281763449 | 5.621409425 | 7.329566588 |
| CCA0789S0063 | CCT1 | 2.959933696 | 3.612409963 | 7.957648918 |
| CCA1700S0006 | CCT1 | 79.27196765 | 64.56651184 | 107.3028503 |
| CCA0376S0001 | CCT2 | 2.201190491 | 1.633138392 | 3.970756512 |
| CCA0836S0060 | CDP-DAGS/CDS4 | 18.53383805 | 10.84182648 | 6.38745166 |
| CCA0786S0021 | CJD1 | 20.09727423 | 9.471212837 | 3.319168033 |
| CCA0678S0115 | CK | 0.024055668 | 0.023966987 | 0.141811945 |
| CCA0678S0116 | CK | 0 | 0 | 0 |
| CCA0859S0182 | CK | 17.43810475 | 10.08895879 | 14.03646321 |
| CCA0852S0031 | CRC | 0 | 0 | 0 |
| CCA0001S0027 | CTS/pxa1 | 9.671155732 | 10.70470142 | 11.0038908 |
| CCA1217S0030 | CTS/pxa1 | 13.18188132 | 13.10561894 | 15.8346251 |
| CCA0953S0053 | DGAT1 | 18.86416048 | 8.092158144 | 11.52054424 |
| CCA1033S0056 | DGAT1 | 0.291924452 | 0.095788587 | 0.100775464 |
| CCA0733S0001 | DGAT2 | 42.63745194 | 48.4417464 | 34.13640512 |
| CCA0877S0027 | DGAT3 | 0 | 0 | 0 |
| CCA0897S0075 | DGDGS/DGD1 | 30.17565201 | 106.9700772 | 143.097889 |
| CCA0932S0050 | DGDGS/DGD2 | 21.95964359 | 18.10443716 | 12.31513189 |
| CCA1681S0005 | DHLAT/EMB3003(E2) | 140.3028697 | 57.55956508 | 15.5077631 |
| CCA0642S0172 | DHLAT/LTA2(E2) | 101.4311875 | 47.33004134 | 10.02789295 |
| CCA0914S0017 | DHLAT/LTA2(E2) | 64.9734494 | 38.02778375 | 7.658108814 |
| CCA0632S0014 | ER/ENR1(MOD1) | 280.1244797 | 184.5199244 | 37.31483276 |
| CCA0688S0093 | ER/ENR1(MOD1) | 143.8523617 | 50.47654169 | 9.798388294 |
| CCA0632S0019 | FAD2 | 716.6539289 | 895.9414493 | 882.5450913 |
| CCA0652S0044 | FAD2 | 292.023871 | 15.89841197 | 11.84728352 |
| CCA0862S0043 | FAD2 | 0.044133138 | 0.054963052 | 0.648529357 |
| CCA0552S0222 | FAD4 | 0.055373098 | 0 | 0 |
| CCA1046S0100 | FAD6 | 5.72280665 | 2.115703825 | 3.291809021 |
| CCA0582S0062 | FAD7 | 119.0404702 | 206.5005445 | 38.97416066 |
| CCA0524S0256 | FAD8 | 0.694636894 | 0.0290385 | 0.206850474 |
| CCA0680S0061 | FAD8 | 4.291711631 | 5.261572494 | 3.564760589 |
| CCA0700S0002 | FAD8 | 5.447691733 | 1.279364075 | 4.0434794 |
| CCA0662S0012 | FATA | 0.132742418 | 0.15429524 | 0.126625131 |
| CCA0662S0016 | FATA | 56.11395274 | 55.91372982 | 27.07647161 |
| CCA1410S0013 | FATA | 0 | 0 | 0 |
| CCA1526S0028 | FATA | 79.93731233 | 44.29816806 | 21.68780032 |
| CCA0522S0144 | FATB | 43.35203707 | 17.18955467 | 22.5102756 |
| CCA1144S0043 | FATB | 28.71880858 | 13.00750077 | 9.335671966 |
| CCA1539S0037 | FUS3 | 54.64325532 | 33.25180205 | 7.418638007 |
| CCA0737S0071 | GGGT/SFR2 | 8.956045551 | 10.40526308 | 20.98184867 |
| CCA0587S0214 | GLK1 | 5.346604534 | 3.377515392 | 4.466947635 |
| CCA1017S0007 | GLK1 | 0.029096294 | 0 | 0.013877712 |
| CCA0507S0021 | GPAT/ATS1/ACT1 | 2.502344445 | 2.92855654 | 3.678290463 |
| CCA1046S0006 | GPAT/ATS1/ACT1 | 4.51550616 | 4.131791058 | 3.376622913 |
| CCA0108S0003 | GPAT9 | 7.597602646 | 3.648420242 | 1.716600277 |
| CCA0526S0215 | GPAT9 | 26.23953998 | 24.24510108 | 22.25659834 |
| CCA1498S0029 | GPDH | 12.65584831 | 9.897860316 | 13.63296728 |
| CCA0520S0081 | GPDHP | 6.825272374 | 8.071596456 | 3.115925562 |
| CCA0779S0217 | GPDHP | 56.1796795 | 245.3160074 | 158.4403095 |
| CCA0862S0133 | GPDHc1 | 20.28611264 | 16.18009351 | 6.793558384 |
| CCA0884S0081 | GPDHc1 | 1.986250827 | 0.993901953 | 1.66329778 |
| CCA0555S0082 | HACPS | 10.76920047 | 8.640652258 | 8.244463323 |
| CCA0063S0002 | HAD | 3.962449695 | 2.208721501 | 0.649933629 |
| CCA1422S0013 | HAD | 197.1173116 | 64.39382612 | 24.85927783 |
| CCA0524S0169 | HSI2/VAL1 | 39.77240362 | 24.60480271 | 13.74624353 |
| CCA0628S0063 | HSI2/VAL1 | 10.93522959 | 9.552494392 | 8.988904388 |
| CCA0905S0016 | HSI2/VAL1 | 26.8629441 | 29.81128253 | 23.74418385 |
| CCA0729S0022 | HSL1/VAL2 | 18.84409498 | 18.86387584 | 17.61919539 |
| CCA0799S0147 | HSL1/VAL2 | 26.45393727 | 26.32981937 | 22.39874512 |
| CCA0299S0001 | KAR | 0.735040931 | 0.254656944 | 0.461843444 |
| CCA0513S0068 | KAR | 184.9639458 | 189.9783529 | 85.86317661 |
| CCA0791S0077 | KAR | 32.16292436 | 28.69163109 | 23.01482581 |
| CCA0828S0028 | KAR | 12.94757213 | 6.213441188 | 3.872360062 |
| CCA0828S0029 | KAR | 9.893796354 | 28.29695814 | 64.25439667 |
| CCA1453S0052 | KAR | 0 | 0 | 0 |
| CCA0549S0009 | KASI | 5.959334044 | 1.16943104 | 1.112805433 |
| CCA0616S0150 | KASI | 69.240278 | 36.7382273 | 6.727318949 |
| CCA0662S0123 | KASI | 151.3990919 | 83.63651816 | 26.43749475 |
| CCA0977S0052 | KASII | 42.97416594 | 41.08107009 | 20.2425197 |
| CCA1225S0016 | KASII | 97.41679852 | 156.5424176 | 107.1087132 |
| CCA0533S0062 | KASIII | 25.52219908 | 30.03071018 | 19.94648213 |
| CCA0674S0169 | KASIII | 22.2063757 | 16.36902922 | 10.67168109 |
| CCA0646S0081 | LACS8 | 74.15487829 | 79.04845842 | 80.90875057 |
| CCA1255S0007 | LACS8 | 2.69522614 | 3.408905627 | 2.830683268 |
| CCA0803S0023 | LACS9 | 49.24859378 | 48.61085748 | 15.04677248 |
| CCA1377S0027 | LACS9 | 0.019929433 | 0.059567891 | 0.369193547 |
| CCA0223S0002 | LCAT-PLA | 0.078426388 | 0.026045756 | 0.08329033 |
| CCA0627S0072 | LCAT-PLA | 0.28501841 | 0.112219525 | 0.221919098 |
| CCA0822S0036 | LCAT-PLA | 10.17442959 | 8.710461081 | 5.453523297 |
| CCA0806S0066 | LEC1 | 6.689850957 | 0.071799834 | 0 |
| CCA0973S0020 | LPAAT1 | 5.781449949 | 5.614824157 | 7.402511622 |
| CCA1348S0036 | LPAAT1 | 4.005758199 | 3.097230572 | 3.782524755 |
| CCA0642S0092 | LPAAT2 | 0 | 0.037814579 | 0 |
| CCA0862S0089 | LPAAT2 | 27.4289458 | 28.33164556 | 16.66707087 |
| CCA1288S0029 | LPAAT2 | 0 | 0 | 0 |
| CCA1288S0032 | LPAAT3 | 0 | 0.044662889 | 0 |
| CCA1528S0008 | LPAAT4 | 0.780031539 | 0.723703568 | 0.730716211 |
| CCA0659S0074 | LPAAT5 | 30.81747814 | 17.4222675 | 17.53114281 |
| CCA0782S0028 | LPCAT1 | 112.5198372 | 92.4488753 | 77.51769426 |
| CCA1223S0023 | LPCAT1 | 0.388997028 | 0.070581511 | 0.110582128 |
| CCA1742S0016 | LPD2(E3) | 283.5090181 | 215.4175499 | 72.70137122 |
| CCA0513S0143 | LS | 9.283976689 | 5.416676992 | 3.271969094 |
| CCA0851S0001 | LS | 0 | 0 | 0 |
| CCA0852S0033 | LS | 20.73851587 | 14.6693724 | 8.557230661 |
| CCA1687S0001 | LS | 0.021674523 | 0 | 0 |
| CCA0524S0209 | MCMT | 4.940982177 | 2.528130006 | 0.781871622 |
| CCA0905S0078 | MCMT | 82.86349174 | 55.20087405 | 18.56617193 |
| CCA0698S0157 | MGDGS/MGD1 | 12.7023707 | 7.110149372 | 9.04891709 |
| CCA0904S0057 | MGDGS/MGD1 | 4.676299037 | 6.306982572 | 5.082585694 |
| CCA0638S0156 | MGDGS/MGD2 | 0.726565771 | 0.422626796 | 1.120447605 |
| CCA1172S0021 | MGDGS/MGD2 | 0.112601981 | 0.092977497 | 0.493202299 |
| CCA1766S0007 | MGDGS/MGD2 | 0.143592452 | 0.240523447 | 0.133321951 |
| CCA1766S0009 | MGDGS/MGD2 | 0.077779245 | 0.178828873 | 0.159804346 |
| CCA0674S0096 | OBO | 0 | 0 | 0 |
| CCA0514S0208 | OLE1 | 4166.902546 | 6221.348283 | 3474.265551 |
| CCA0656S0072 | OLE1 | 593.0195825 | 253.6197667 | 413.5056485 |
| CCA0656S0074 | OLE1 | 1006.691272 | 915.2177902 | 1375.192802 |
| CCA0520S0025 | OLE4 | 0 | 0 | 0 |
| CCA0779S0183 | OLE4 | 1397.132383 | 4237.367641 | 2222.292087 |
| CCA0791S0002 | OLE4 | 472.6707597 | 1488.658676 | 1341.424575 |
| CCA0754S0077 | PDAT1 | 8.635328374 | 2.244390234 | 3.864043645 |
| CCA0953S0023 | PDAT1 | 11.38448562 | 5.951533545 | 6.856146727 |
| CCA1591S0012 | PDAT1 | 32.67314002 | 36.04103978 | 13.03786323 |
| CCA0665S0050 | PDAT2 | 62.8776459 | 184.5118066 | 85.71331902 |
| CCA0526S0017 | PDCT/ROD1 | 81.8532483 | 83.91588469 | 63.76126684 |
| CCA0526S0249 | PII | 413.4792719 | 402.9971405 | 284.2571468 |
| CCA0886S0038 | PII | 0.087140431 | 0.043409594 | 0 |
| CCA1057S0037 | PII | 181.618775 | 148.2304268 | 55.26508642 |
| CCA1057S0041 | PII | 0.334801002 | 0.103584153 | 0.131881483 |
| CCA0904S0081 | PKL | 42.20429012 | 28.97251393 | 22.75050324 |
| CCA0982S0040 | PP/AtLPP3 | 0.775381254 | 0.254589365 | 0.884977404 |
| CCA1663S0012 | PP/AtLPP3 | 4.187951792 | 4.217588641 | 3.900384866 |
| CCA1705S0022 | PP/AtLPP3 | 33.94095611 | 50.5830702 | 52.744727 |
| CCA0674S0176 | PP/LPP-beta | 13.86050483 | 3.675863892 | 3.364605813 |
| CCA0555S0221 | PP/LPP-delta | 12.96873028 | 14.98669006 | 7.103449367 |
| CCA0862S0017 | PP/LPP-delta | 22.20669189 | 19.73789985 | 12.45248025 |
| CCA0935S0046 | PP/LPP-gamma | 7.182343862 | 4.720706475 | 5.899258355 |
| CCA1370S0043 | PP/LPP-gamma | 0.013469657 | 0.026840001 | 0 |
| CCA0982S0041 | PP/LPP2 | 0.790235812 | 0.615479102 | 0.607760558 |
| CCA1425S0006 | PP/LPP2 | 0.090848534 | 0 | 0.057774587 |
| CCA1663S0013 | PP/LPP2 | 6.404028341 | 0.950982894 | 0.573964088 |
| CCA0810S0117 | PP/PAH2 | 11.25590608 | 10.23800926 | 8.043584003 |
| CCA0607S0021 | PPIase | 26.0131046 | 17.548676 | 18.12729092 |
| CCA1154S0006 | PPIase | 45.7456587 | 42.11650296 | 45.90217202 |
| CCA0763S0006 | SAD/DES5 | 0 | 0 | 0 |
| CCA1391S0034 | SAD/DES6 | 2203.92652 | 1658.435204 | 318.1194194 |
| CCA0500S0109 | SAD/FAB2 | 417.7923401 | 379.4942654 | 334.505828 |
| CCA0500S0077 | SAD/FAB2 | 0.148374247 | 0.147827265 | 0.015726287 |
| CCA0500S0078 | SAD/FAB2 | 0.113359676 | 0.263530808 | 0.144899214 |
| CCA0500S0081 | SAD/FAB2 | 0.158563798 | 0.217373011 | 0.395972268 |
| CCA0500S0082 | SAD/FAB2 | 0 | 0 | 0.017356996 |
| CCA0500S0083 | SAD/FAB2 | 0 | 0 | 0 |
| CCA0763S0005 | SAD/FAB2 | 0 | 0 | 0 |
| CCA0789S0100 | SAD/FAB2 | 301.291133 | 289.5428876 | 103.6671113 |
| CCA0587S0126 | SLS/SQD2 | 4.7959467 | 2.877755258 | 8.543929683 |
| CCA1559S0025 | SLS/SQD2 | 3.981557599 | 0.741004873 | 1.478423251 |
| CCA1228S0022 | SQS/SQD1 | 38.50637573 | 33.44162548 | 14.9422079 |
| CCA0642S0015 | STERO | 86.61663603 | 209.0380981 | 302.2594112 |
| CCA1330S0018 | STERO | 1.49136566 | 4.150845985 | 34.05102231 |
| CCA1524S0080 | STERO | 0 | 0 | 0.077116887 |
| CCA0411S0001 | TAGL-Like | 3.883372296 | 5.835221323 | 2.553686044 |
| CCA0500S0230 | TAGL-Like | 7.583060082 | 15.13105165 | 10.36817157 |
| CCA0583S0079 | TAGL-Like | 0.015707717 | 0.015649811 | 0.240936033 |
| CCA0678S0189 | TAGL-Like | 14.53809468 | 11.69934717 | 16.39322429 |
| CCA0774S0025 | TAGL-Like | 46.55077289 | 8.220107304 | 0.437323245 |
| CCA0975S0068 | TAGL-Like | 9.281990114 | 10.40128167 | 9.690733026 |
| CCA1071S0009 | TAGL-Like | 18.14047385 | 12.30305245 | 4.235504239 |
| CCA1085S0034 | TAGL-Like | 41.2733079 | 61.3570784 | 42.86669982 |
| CCA1326S0025 | TAGL-Like | 0 | 0 | 0 |
| CCA0670S0126 | TAGL-Like/LIP1 | 30.60817482 | 29.38671116 | 42.79485282 |
| CCA0520S0150 | TAGL-Like/MPL1 | 0.846238316 | 0.877038381 | 2.30571612 |
| CCA0520S0151 | TAGL-Like/MPL1 | 0.338277575 | 0 | 0 |
| CCA0686S0009 | TAGL-Like/MPL1 | 0.214488047 | 0.06783367 | 0.043421316 |
| CCA0779S0268 | TAGL-Like/MPL1 | 16.57928731 | 25.09449635 | 82.96677702 |
| CCA0779S0269 | TAGL-Like/MPL1 | 0 | 0.010057069 | 0.30851495 |
| CCA1126S0018 | TAGL-Like/MPL1 | 0 | 0.017153791 | 0 |
| CCA1144S0026 | TAGL-Like/MPL1 | 0 | 0 | 0 |
| CCA1094S0080 | TAGL-Like/SAG101 | 3.07790466 | 0.712155421 | 3.325612467 |
| CCA1094S0081 | TAGL-Like/SAG101 | 1.322721903 | 0.325110586 | 3.169827858 |
| CCA1368S0013 | TAGL-Like/SAG101 | 0 | 0.025172427 | 0.157378527 |
| CCA0681S0054 | TAGL-Like/SDP1 | 0 | 0.05171809 | 0 |
| CCA0704S0159 | TAGL-Like/SDP1 | 2.301998049 | 1.888559691 | 2.700096736 |
| CCA1566S0007 | TAGL-Like/SDP1 | 0 | 0.012475484 | 0.011944599 |
| CCA1673S0032 | TAGL-Like/SDP1 | 0.538610347 | 0.42527162 | 0.450875141 |
| CCA1673S0033 | TAGL-Like/SDP1 | 0.367154932 | 0.616456289 | 0.177625859 |
| CCA1332S0002 | TGD1 | 18.72867616 | 14.52819526 | 18.13351683 |
| CCA0535S0023 | TOC159 | 25.90154451 | 18.38089497 | 19.38017647 |
| CCA0701S0073 | TOC159 | 1.27450953 | 1.008921601 | 0.844180263 |
| CCA0597S0117 | TT2 | 0 | 0 | 0 |
| CCA0754S0090 | TT2 | 0.021190477 | 0 | 0 |
| CCA0754S0092 | TT2 | 0.094515598 | 0.132941881 | 0.031821159 |
| CCA0848S0108 | TT2 | 0.419809098 | 0.321457053 | 1.387863819 |
| CCA0881S0005 | TT2 | 0 | 0 | 0 |
| CCA0895S0001 | TT2 | 0 | 0 | 0 |
| CCA0953S0011 | TT2 | 0.084391302 | 0.030728139 | 0.1314094 |
| CCA1382S0024 | TT2 | 21.22201512 | 1.778331709 | 3.267726555 |
| CCA0535S0121 | TT8/BHLH42 | 2.621986505 | 0.487024741 | 2.864041923 |
| CCA1400S0020 | TT8/BHLH42 | 5.932471126 | 1.065049446 | 4.935664961 |
| CCA0520S0225 | WRI1 | 0 | 0 | 0 |
| CCA0578S0089 | WRI1 | 137.7944363 | 75.91760681 | 23.76633147 |
| CCA1541S0001 | WRI1 | 11.96780896 | 9.538264764 | 4.645944356 |
| CCA1573S0032 | WRI1 | 79.78608428 | 63.87672038 | 26.01791425 |
| CCA0546S0058 | WRI3 | 0.051000571 | 0.016937519 | 0.021730075 |
| CCA0589S0078 | WRI3 | 0.246920421 | 0.035158183 | 0.022441369 |
| CCA0973S0046 | WRI3 | 0 | 0 | 0 |
| CCA1074S0044 | WRI3 | 0.014877635 | 0.014822788 | 0 |
| CCA0632S0168 | alpha-CT | 4.168095025 | 2.307536818 | 1.685646887 |
| CCA1277S0024 | alpha-CT | 196.896131 | 209.7019363 | 115.2526103 |
| CCA1559S0001 | alpha-CT | 120.2753232 | 164.0186685 | 78.18326383 |
| CCA0607S0051 | alpha-PDH | 456.4547328 | 489.6314625 | 202.4502899 |
| CCA1615S0018 | beta-CT | 2.611460016 | 1.870001798 | 0.947399311 |
| CCA0616S0115 | beta-PDH | 34.87793097 | 63.27792111 | 13.51458214 |
| CCA1016S0003 | beta-PDH | 132.8255309 | 81.71076816 | 27.57180741 |

**Table S23 The list of genes related to non-structure polyphenol metabolism in 11 species including pecan and Chinese hickory.**

|  |  | *Cca* | *Cil* | *Jre* | *Fve* | *Ppe* | *Tca* | *Vvi* | *Zju* | *Mtr* | *Ath* | *Sly* |
| --- | --- | --- | --- | --- | --- | --- | --- | --- | --- | --- | --- | --- |
| Synthesis | **CHS** | **4** | **4** | **4** | **2** | **3** | **2** | **3** | **3** | **1** | **1** | **2** |
|  | CHI | 2 | 2 | 3 | 2 | 2 | 3 | 2 | 3 | 8 | 2 | 3 |
|  | F3'5'H | 2 | 2 | 2 | 2 | 2 | 2 | 3 | 4 | 4 | 2 | 2 |
|  | F3H | 1 | 1 | 1 | 1 | 1 | 1 | 2 | 1 | 1 | 1 | 1 |
|  | F3'H | 4 | 5 | 3 | 3 | 6 | 3 | 6 | 3 | 6 | 1 | 5 |
|  | DFR | 2 | 3 | 2 | 3 | 2 | 4 | 2 | 3 | 3 | 1 | 2 |
|  | **LAR** | **3** | **3** | **5** | **1** | **2** | **2** | **3** | **2** | **1** | **0** | **0** |
|  | LDOX | 1 | 1 | 1 | 1 | 1 | 1 | 1 | 5 | 1 | 1 | 1 |
|  | ANR | 3 | 3 | 4 | 3 | 5 | 4 | 5 | 2 | 5 | 1 | 4 |
| Transfer | GSTF | 1 | 1 | 1 | 1 | 1 | 1 | 2 | 1 | 1 | 1 | 1 |
|  | TT12 | 2 | 2 | 2 | 5 | 3 | 3 | 2 | 1 | 4 | 1 | 1 |
|  | AHA | 1 | 2 | 2 | 1 | 1 | 1 | 1 | 2 | 1 | 1 | 0 |
| Transcription factor | TT2-MYB123 | 6 | 4 | 7 | 5 | 5 | 0 | 3 | 6 | 2 | 2 | 3 |
|  | MYB5 | 11 | 10 | 18 | 7 | 6 | 7 | 13 | 6 | 9 | 10 | 5 |
|  | bHLH | 4 | 4 | 4 | 2 | 1 | 2 | 2 | 3 | 1 | 3 | 1 |
|  | WDR | 2 | 2 | 2 | 2 | 2 | 2 | 2 | 7 | 2 | 3 | 3 |
|  | **WRKY** | **2** | **2** | **2** | **1** | **1** | **1** | **1** | **1** | **1** | **1** | **1** |

Note: *Cca, Carya cathayensis; Cil, Carya illinoinensis; Jre, Juglans regia; Fve, Fragaria vesca; Ppe, Prunus persica; Tca, Theobroma cacao; Vvi, Vitis vinifera; Zju, Ziziphus jujube; Mtr, Medicago truncatula; Ath, Arabidopsis thaliana; Sly, Solanum lycopersicum.*

**Table S24 Gene copy number of enzymes involved in (semi-) essential amino acid biosynthesis.**

| Item | Enzyme ID | *Ath* | *Cca* | *Cil* | *Jre* | *Bna* | *Tca* | *Ghi* | *Gma* | *Sin* | *Lus* | *Hvu* | *Osa* | *Oeu* | *Stu* | *Sbi* | *Tae* | *Rco* | *Egu* | *Zju* | *Adu* | *Han* | *Zma* |
| --- | --- | --- | --- | --- | --- | --- | --- | --- | --- | --- | --- | --- | --- | --- | --- | --- | --- | --- | --- | --- | --- | --- | --- |
| Arginine | 1.2.1.38 | 2 | 52 | 42 | 48 | 106 | 39 | 118 | 74 | 25 | 57 | 34 | 40 | 47 | 54 | 51 | 134 | 31 | 44 | 46 | 35 | 59 | 57 |
|  | 2.1.3.3 | 1 | 2 | 2 | 2 | 7 | 2 | 2 | 6 | 3 | 3 | 2 | 2 | 3 | 3 | 2 | 6 | 2 | 3 | 3 | 3 | 3 | 9 |
|  | 2.3.1.1 | 57 | 174 | 192 | 206 | 435 | 159 | 373 | 288 | 148 | 291 | 142 | 186 | 178 | 221 | 178 | 770 | 125 | 135 | 250 | 177 | 307 | 216 |
|  | 2.6.1.11 | 1 | 11 | 12 | 13 | 34 | 6 | 20 | 19 | 7 | 14 | 7 | 10 | 13 | 9 | 8 | 25 | 8 | 9 | 9 | 17 | 12 | 21 |
|  | 2.7.2.8 | 1 | 5 | 7 | 5 | 16 | 4 | 12 | 9 | 6 | 9 | 5 | 6 | 5 | 3 | 6 | 20 | 4 | 7 | 3 | 5 | 5 | 12 |
|  | 3.5.1.16 | 1 | 14 | 13 | 13 | 19 | 13 | 28 | 19 | 6 | 16 | 8 | 9 | 5 | 8 | 11 | 40 | 7 | 9 | 17 | 11 | 9 | 11 |
|  | 4.3.2.1 | 1 | 4 | 3 | 4 | 13 | 3 | 7 | 8 | 3 | 7 | 3 | 3 | 3 | 2 | 3 | 8 | 3 | 3 | 2 | 4 | 4 | 6 |
|  | 6.3.4.5 | 1 | 3 | 2 | 3 | 7 | 1 | 2 | 4 | 1 | 4 | 1 | 2 | 3 | 1 | 2 | 3 | 2 | 2 | 8 | 1 | 2 | 1 |
|  | 6.3.5.5 | 2 | 10 | 9 | 7 | 27 | 6 | 19 | 16 | 3 | 8 | 6 | 7 | 5 | 6 | 8 | 20 | 6 | 6 | 7 | 9 | 10 | 12 |
|  |  |  |  |  |  |  |  |  |  |  |  |  |  |  |  |  |  |  |  |  |  |  |  |
| Leucine/ L-Isoleucine | 1.1.1.85 | 3 | 8 | 11 | 10 | 44 | 7 | 18 | 15 | 5 | 14 | 8 | 8 | 8 | 7 | 7 | 25 | 6 | 11 | 9 | 14 | 14 | 14 |
|  | 2.3.3.13 | 2 | 1 | 1 | 0 | 4 | 1 | 4 | 5 | 2 | 0 | 0 | 2 | 2 | 3 | 2 | 3 | 1 | 1 | 1 | 3 | 2 | 3 |
|  | 2.6.1.6 | 1 | 2 | 1 | 2 | 7 | 1 | 3 | 3 | 1 | 2 | 0 | 1 | 1 | 0 | 2 | 1 | 1 | 1 | 1 | 2 | 1 | 2 |
|  | 2.6.1.42 | 7 | 8 | 7 | 8 | 32 | 10 | 23 | 17 | 5 | 12 | 8 | 8 | 10 | 10 | 9 | 24 | 8 | 9 | 9 | 8 | 10 | 8 |
|  | 4.2.1.33 | 4 | 5 | 7 | 6 | 21 | 6 | 24 | 12 | 6 | 14 | 4 | 4 | 3 | 5 | 6 | 15 | 5 | 6 | 9 | 6 | 10 | 13 |
|  |  |  |  |  |  |  |  |  |  |  |  |  |  |  |  |  |  |  |  |  |  |  |  |
| L-Threonine | 2.7.1.39 | 1 | 5 | 6 | 4 | 15 | 5 | 10 | 10 | 7 | 9 | 3 | 4 | 6 | 4 | 5 | 14 | 5 | 4 | 6 | 4 | 6 | 6 |
|  | 4.2.3.1 | 10 | 67 | 39 | 62 | 122 | 45 | 116 | 65 | 49 | 62 | 30 | 49 | 78 | 65 | 50 | 179 | 44 | 25 | 55 | 51 | 95 | 57 |
|  |  |  |  |  |  |  |  |  |  |  |  |  |  |  |  |  |  |  |  |  |  |  |  |
| Lysine | 1.2.1.11 | 1 | 3 | 2 | 2 | 8 | 2 | 6 | 4 | 2 | 4 | 2 | 3 | 2 | 3 | 2 | 5 | 2 | 3 | 2 | 2 | 3 | 1 |
|  | 1.17.1.8 | 2 | 2 | 1 | 5 | 6 | 1 | 6 | 4 | 3 | 4 | 0 | 3 | 2 | 2 | 3 | 7 | 2 | 2 | 2 | 2 | 3 | 3 |
|  | 2.6.1.83 | 2 | 47 | 42 | 44 | 140 | 34 | 94 | 64 | 34 | 59 | 28 | 37 | 37 | 40 | 33 | 99 | 29 | 30 | 31 | 33 | 54 | 44 |
|  | 2.7.2.4 | 5 | 27 | 27 | 26 | 55 | 15 | 41 | 38 | 14 | 26 | 9 | 20 | 23 | 16 | 20 | 48 | 16 | 21 | 16 | 18 | 29 | 25 |
|  | 4.1.1.20 | 2 | 4 | 5 | 5 | 11 | 6 | 14 | 7 | 4 | 6 | 4 | 4 | 5 | 6 | 7 | 18 | 4 | 5 | 2 | 6 | 3 | 7 |
|  | 4.3.3.7 | 2 | 2 | 2 | 2 | 8 | 1 | 4 | 3 | 1 | 2 | 1 | 2 | 3 | 2 | 2 | 6 | 1 | 2 | 2 | 2 | 2 | 2 |
|  | 5.1.1.7 | 1 | 1 | 1 | 1 | 7 | 1 | 2 | 2 | 1 | 1 | 1 | 0 | 1 | 0 | 2 | 2 | 2 | 1 | 1 | 1 | 1 | 2 |
|  |  |  |  |  |  |  |  |  |  |  |  |  |  |  |  |  |  |  |  |  |  |  |  |
| Histidine | 1.1.1.23 | 2 | 54 | 45 | 41 | 140 | 43 | 112 | 92 | 54 | 87 | 40 | 66 | 59 | 53 | 78 | 232 | 35 | 60 | 88 | 61 | 80 | 73 |
|  | 2.4.2.17 | 3 | 1 | 1 | 1 | 8 | 1 | 3 | 2 | 2 | 2 | 1 | 1 | 1 | 1 | 1 | 3 | 1 | 2 | 1 | 1 | 2 | 1 |
|  | 2.6.1.9 | 6 | 62 | 57 | 60 | 184 | 42 | 123 | 90 | 44 | 78 | 37 | 49 | 55 | 61 | 44 | 138 | 39 | 41 | 43 | 56 | 72 | 70 |
|  | 3.1.3.15 | 1 | 14 | 10 | 11 | 27 | 7 | 26 | 15 | 8 | 13 | 6 | 9 | 11 | 7 | 7 | 23 | 7 | 7 | 10 | 10 | 9 | 7 |
|  | 3.5.4.19 | 1 | 1 | 1 | 1 | 4 | 1 | 1 | 1 | 1 | 1 | 0 | 1 | 1 | 1 | 2 | 3 | 0 | 1 | 2 | 1 | 2 | 1 |
|  | 3.6.1.31 | 1 | 1 | 1 | 1 | 4 | 1 | 1 | 1 | 1 | 1 | 0 | 1 | 1 | 1 | 2 | 3 | 0 | 1 | 2 | 1 | 2 | 1 |
|  | 4.2.1.19 | 2 | 2 | 2 | 5 | 4 | 1 | 1 | 2 | 4 | 2 | 1 | 1 | 2 | 1 | 1 | 4 | 1 | 1 | 1 | 2 | 2 | 0 |
|  | 4.3.2.-. | 3 | 4 | 3 | 4 | 13 | 3 | 7 | 8 | 3 | 7 | 3 | 3 | 3 | 2 | 3 | 8 | 3 | 3 | 2 | 4 | 4 | 6 |
|  | 5.3.1.16 | 1 | 2 | 1 | 2 | 4 | 1 | 2 | 6 | 1 | 2 | 2 | 1 | 1 | 1 | 2 | 3 | 1 | 1 | 2 | 2 | 1 | 2 |
|  |  |  |  |  |  |  |  |  |  |  |  |  |  |  |  |  |  |  |  |  |  |  |  |
| Methionine | 2.1.1.10 | 13 | 24 | 30 | 29 | 50 | 27 | 58 | 43 | 24 | 34 | 18 | 18 | 25 | 25 | 25 | 64 | 21 | 25 | 30 | 22 | 45 | 33 |
|  | 2.1.1.14 | 6 | 44 | 42 | 47 | 102 | 42 | 59 | 50 | 36 | 33 | 15 | 34 | 29 | 35 | 33 | 95 | 22 | 22 | 59 | 25 | 53 | 32 |
|  | 2.5.1.- | 141 | 181 | 177 | 177 | 492 | 170 | 347 | 277 | 155 | 230 | 139 | 163 | 179 | 220 | 189 | 614 | 136 | 146 | 163 | 187 | 231 | 198 |
|  | 2.7.1.39 | 1 | 5 | 6 | 4 | 15 | 5 | 10 | 10 | 7 | 9 | 3 | 4 | 6 | 4 | 5 | 14 | 5 | 4 | 6 | 4 | 6 | 6 |
|  |  |  |  |  |  |  |  |  |  |  |  |  |  |  |  |  |  |  |  |  |  |  |  |
| Phenylalanine | 1.13.11.27 | 1 | 5 | 4 | 7 | 10 | 3 | 13 | 8 | 1 | 7 | 3 | 3 | 7 | 3 | 3 | 10 | 3 | 4 | 3 | 4 | 5 | 6 |
|  | 2.6.1.- | 43 | 91 | 80 | 82 | 252 | 65 | 179 | 141 | 61 | 112 | 54 | 68 | 78 | 82 | 68 | 193 | 59 | 69 | 71 | 82 | 101 | 91 |
|  | 2.6.1.79 | 1 | 47 | 42 | 44 | 140 | 34 | 94 | 64 | 34 | 59 | 28 | 37 | 37 | 40 | 33 | 99 | 29 | 30 | 31 | 33 | 54 | 44 |
|  | 4.2.1.51 | 6 | 14 | 13 | 12 | 23 | 7 | 16 | 12 | 5 | 14 | 7 | 13 | 11 | 4 | 9 | 25 | 4 | 11 | 7 | 7 | 19 | 14 |
|  | 4.2.1.91 | 6 | 14 | 13 | 12 | 23 | 7 | 16 | 12 | 5 | 14 | 7 | 13 | 11 | 4 | 9 | 25 | 4 | 11 | 7 | 7 | 19 | 14 |
|  | 5.4.99.5 | 7 | 8 | 12 | 5 | 15 | 10 | 19 | 12 | 5 | 7 | 2 | 6 | 10 | 6 | 9 | 25 | 4 | 2 | 7 | 10 | 11 | 9 |
|  |  |  |  |  |  |  |  |  |  |  |  |  |  |  |  |  |  |  |  |  |  |  |  |
| Tryptophan | 2.4.2.18 | 1 | 2 | 2 | 2 | 4 | 1 | 2 | 1 | 1 | 2 | 0 | 1 | 1 | 2 | 1 | 3 | 0 | 1 | 1 | 1 | 2 | 1 |
|  | 4.1.1.48 | 2 | 2 | 1 | 1 | 8 | 3 | 4 | 2 | 0 | 3 | 3 | 3 | 4 | 1 | 4 | 10 | 1 | 2 | 3 | 2 | 2 | 3 |
|  | 4.1.3.27 | 9 | 6 | 9 | 11 | 26 | 4 | 19 | 12 | 4 | 9 | 7 | 7 | 14 | 5 | 9 | 21 | 5 | 7 | 6 | 6 | 8 | 14 |
|  | 4.2.1.20 | 6 | 19 | 22 | 22 | 66 | 16 | 42 | 34 | 13 | 23 | 17 | 22 | 26 | 17 | 21 | 74 | 19 | 18 | 25 | 17 | 23 | 29 |
|  | 5.3.1.24 | 3 | 3 | 2 | 3 | 2 | 1 | 3 | 2 | 1 | 2 | 1 | 1 | 1 | 2 | 2 | 3 | 1 | 1 | 1 | 1 | 1 | 2 |
|  |  |  |  |  |  |  |  |  |  |  |  |  |  |  |  |  |  |  |  |  |  |  |  |
| Valine | 1.1.1.86 | 1 | 2 | 2 | 3 | 4 | 3 | 4 | 4 | 1 | 2 | 1 | 2 | 1 | 2 | 2 | 2 | 2 | 2 | 2 | 2 | 2 | 3 |
|  | 2.2.1.6 | 3 | 10 | 8 | 4 | 20 | 7 | 19 | 14 | 8 | 14 | 4 | 7 | 8 | 8 | 6 | 23 | 8 | 7 | 5 | 7 | 13 | 10 |
|  | 2.6.1.42 | 7 | 8 | 7 | 8 | 32 | 10 | 23 | 17 | 5 | 12 | 8 | 8 | 10 | 10 | 9 | 24 | 8 | 9 | 9 | 8 | 10 | 8 |
|  | 4.2.1.9 | 9 | 305 | 275 | 310 | 691 | 258 | 630 | 427 | 279 | 416 | 253 | 339 | 304 | 444 | 349 | 1249 | 214 | 216 | 355 | 282 | 537 | 292 |

Note: *Ath*, *Arabidopsis thaliana*; *Cca*, *Carya cathayensis*; *Cil*, *Carya illinoinensis*; *Jre*, *Juglans regia*; *Bna*, *Brassica napus*; *Tca*, *Theobroma cacao*; *Ghi*, *Gossypium hirsutum*; *Gma*, *Glycine max*; *Sin*, *Sesamum indium*; *Lus*, *Linum usitatissimum*; *Hvu*, *Hordeum vulgare*; *Osa*, *Oryza sativa*; *Oeu*, *Olea europaea*; *Stu*, *Solanum tuberosum*; *Sbi*, *Sorghum bicolor*; *Tae*, *Triticum aestivum*; *Rco*, *Ricinus communis*; *Egu*, *Elaeis guineensis*; *Zju*, *Ziziphus jujube*; *Adu*, *Arachis duranensis*; *Han*, *Helianthus annuus*; *Zma*, *Zea mays*.

**Table S25 Gene copy number of enzymes involved in B vitamin biosynthesis.**

| Item | Enzyme ID | *Ath* | *Cca* | *Cil* | *Jre* | *Bna* | *Tca* | *Ghi* | *Gma* | *Sin* | *Lus* | *Hvu* | *Osa* | *Oeu* | *Stu* | *Sbi* | *Tae* | *Rco* | *Egu* | *Zju* | *Adu* | *Han* | *Zma* |
| --- | --- | --- | --- | --- | --- | --- | --- | --- | --- | --- | --- | --- | --- | --- | --- | --- | --- | --- | --- | --- | --- | --- | --- |
| Thiamine (B1) | 2.5.1.3 | 3 | 15 | 16 | 19 | 44 | 13 | 29 | 39 | 12 | 23 | 13 | 15 | 18 | 14 | 13 | 43 | 11 | 11 | 12 | 25 | 20 | 21 |
|  | 3.1.3.100 | 1 | 1 | 1 | 1 | 2 | 1 | 2 | 2 | 1 | 1 | 1 | 1 | 1 | 1 | 1 | 3 | 1 | 1 | 0 | 1 | 1 | 1 |
|  |  |  |  |  |  |  |  |  |  |  |  |  |  |  |  |  |  |  |  |  |  |  |  |
| Pantothenic acid (B5) | 2.1.2.11 | 2 | 1 | 1 | 1 | 4 | 1 | 3 | 2 | 1 | 2 | 1 | 2 | 0 | 2 | 3 | 3 | 1 | 2 | 1 | 2 | 1 | 3 |
|  | 2.7.1.33 | 2 | 3 | 4 | 4 | 8 | 2 | 6 | 6 | 3 | 6 | 2 | 2 | 3 | 2 | 2 | 6 | 2 | 3 | 2 | 3 | 3 | 3 |
|  | 6.3.2.1 | 5 | 4 | 4 | 4 | 10 | 3 | 4 | 8 | 4 | 5 | 3 | 2 | 3 | 2 | 3 | 10 | 3 | 4 | 5 | 4 | 5 | 3 |
|  |  |  |  |  |  |  |  |  |  |  |  |  |  |  |  |  |  |  |  |  |  |  |  |
| Vitamin B6 | 1.1.1.65 | 1 | 49 | 38 | 44 | 49 | 33 | 97 | 57 | 26 | 34 | 19 | 31 | 43 | 31 | 39 | 117 | 31 | 30 | 46 | 53 | 68 | 29 |
|  | 1.4.3.5 | 1 | 1 | 1 | 1 | 2 | 1 | 2 | 1 | 1 | 1 | 1 | 1 | 3 | 2 | 1 | 2 | 1 | 1 | 1 | 1 | 1 | 1 |
|  | 2.7.1.35 | 1 | 1 | 1 | 2 | 2 | 1 | 2 | 2 | 0 | 4 | 0 | 1 | 0 | 0 | 1 | 3 | 2 | 1 | 1 | 1 | 0 | 1 |
|  | 3.1.3.74 | 3 | 3 | 4 | 7 | 10 | 5 | 13 | 11 | 2 | 6 | 3 | 3 | 4 | 4 | 3 | 15 | 4 | 4 | 4 | 3 | 5 | 3 |
|  | 4.3.3.6 | 4 | 3 | 6 | 5 | 7 | 3 | 13 | 4 | 3 | 8 | 4 | 5 | 9 | 3 | 2 | 10 | 4 | 3 | 6 | 4 | 5 | 5 |
|  |  |  |  |  |  |  |  |  |  |  |  |  |  |  |  |  |  |  |  |  |  |  |  |
| Riboflavin (B2) | 1.1.1.193 | 2 | 8 | 8 | 9 | 21 | 6 | 14 | 20 | 6 | 15 | 6 | 8 | 12 | 5 | 8 | 27 | 7 | 7 | 7 | 10 | 12 | 13 |
|  | 2.5.1.9 | 2 | 8 | 8 | 8 | 21 | 6 | 16 | 13 | 6 | 12 | 6 | 7 | 9 | 9 | 5 | 20 | 6 | 4 | 5 | 9 | 9 | 12 |
|  | 2.5.1.78 | 1 | 1 | 1 | 1 | 4 | 1 | 4 | 1 | 1 | 2 | 3 | 1 | 1 | 2 | 2 | 3 | 1 | 1 | 1 | 1 | 2 | 1 |
|  | 2.7.1.26 | 1 | 7 | 8 | 5 | 10 | 3 | 14 | 15 | 5 | 9 | 2 | 5 | 4 | 3 | 6 | 13 | 4 | 7 | 4 | 5 | 8 | 4 |
|  | 2.7.1.50 | 1 | 1 | 1 | 1 | 2 | 1 | 2 | 1 | 0 | 1 | 1 | 1 | 1 | 2 | 1 | 7 | 1 | 1 | 1 | 1 | 1 | 1 |
|  | 2.7.7.2 | 8 | 16 | 14 | 14 | 33 | 10 | 54 | 25 | 11 | 20 | 6 | 13 | 13 | 8 | 10 | 26 | 12 | 11 | 12 | 14 | 14 | 20 |
|  | 3.1.3.104 | 1 | 22 | 17 | 22 | 44 | 13 | 34 | 27 | 13 | 30 | 14 | 16 | 18 | 19 | 23 | 57 | 17 | 18 | 21 | 18 | 21 | 23 |
|  | 3.5.4.25 | 3 | 3 | 2 | 4 | 8 | 3 | 4 | 8 | 3 | 6 | 3 | 3 | 2 | 3 | 3 | 8 | 2 | 2 | 4 | 4 | 4 | 4 |
|  | 3.5.4.26 | 2 | 8 | 8 | 9 | 21 | 6 | 14 | 20 | 6 | 15 | 6 | 8 | 12 | 5 | 8 | 27 | 7 | 7 | 7 | 10 | 12 | 13 |
|  | 4.1.99.12 | 3 | 3 | 2 | 4 | 8 | 3 | 4 | 8 | 3 | 6 | 3 | 3 | 2 | 3 | 3 | 8 | 2 | 2 | 4 | 4 | 4 | 4 |
|  |  |  |  |  |  |  |  |  |  |  |  |  |  |  |  |  |  |  |  |  |  |  |  |
| Folate (B9) | 1.5.1.3 | 2 | 3 | 3 | 3 | 5 | 1 | 3 | 4 | 0 | 2 | 1 | 2 | 3 | 1 | 1 | 3 | 2 | 2 | 2 | 3 | 2 | 4 |
|  | 2.7.6.3 | 2 | 1 | 1 | 2 | 2 | 2 | 2 | 2 | 1 | 2 | 1 | 1 | 1 | 1 | 2 | 3 | 1 | 1 | 1 | 2 | 2 | 1 |
|  | 3.4.19.9 | 3 | 4 | 5 | 4 | 16 | 3 | 4 | 5 | 4 | 6 | 2 | 2 | 2 | 5 | 4 | 9 | 3 | 2 | 4 | 4 | 5 | 4 |
|  | 6.3.2.12 | 1 | 4 | 4 | 4 | 10 | 3 | 1 | 7 | 3 | 5 | 2 | 1 | 2 | 1 | 2 | 9 | 3 | 4 | 4 | 2 | 4 | 3 |
|  | 6.3.2.17 | 3 | 4 | 4 | 4 | 10 | 3 | 1 | 7 | 3 | 5 | 2 | 1 | 2 | 1 | 2 | 9 | 3 | 4 | 4 | 2 | 4 | 3 |

Note: *Ath*, *Arabidopsis thaliana*; *Cca*, *Carya cathayensis*; *Cil*, *Carya illinoinensis*; *Jre*, *Juglans regia*; *Bna*, *Brassica napus*; *Tca*, *Theobroma cacao*; *Ghi*, *Gossypium hirsutum*; *Gma*, *Glycine max*; *Sin*, *Sesamum indium*; *Lus*, *Linum usitatissimum*; *Hvu*, *Hordeum vulgare*; *Osa*, *Oryza sativa*; *Oeu*, *Olea europaea*; *Stu*, *Solanum tuberosum*; *Sbi*, *Sorghum bicolor*; *Tae*, *Triticum aestivum*; *Rco*, *Ricinus communis*; *Egu*, *Elaeis guineensis*; *Zju*, *Ziziphus jujube*; *Adu*, *Arachis duranensis*; *Han*, *Helianthus annuus*; *Zma*, *Zea mays*.
