# Supplementary material for: Duphold: scalable, depth-based annotation and curation of high-confidence structural variant calls
Source: Gigascience. 2019 Apr 24;8(4):giz040. doi: 10.1093/gigascience/giz040 (PMC6479422; doi:10.1093/gigascience/giz040)
Supplement: Supplemental Files [file giz040_supplemental_files.zip › Additional file 2.docx]

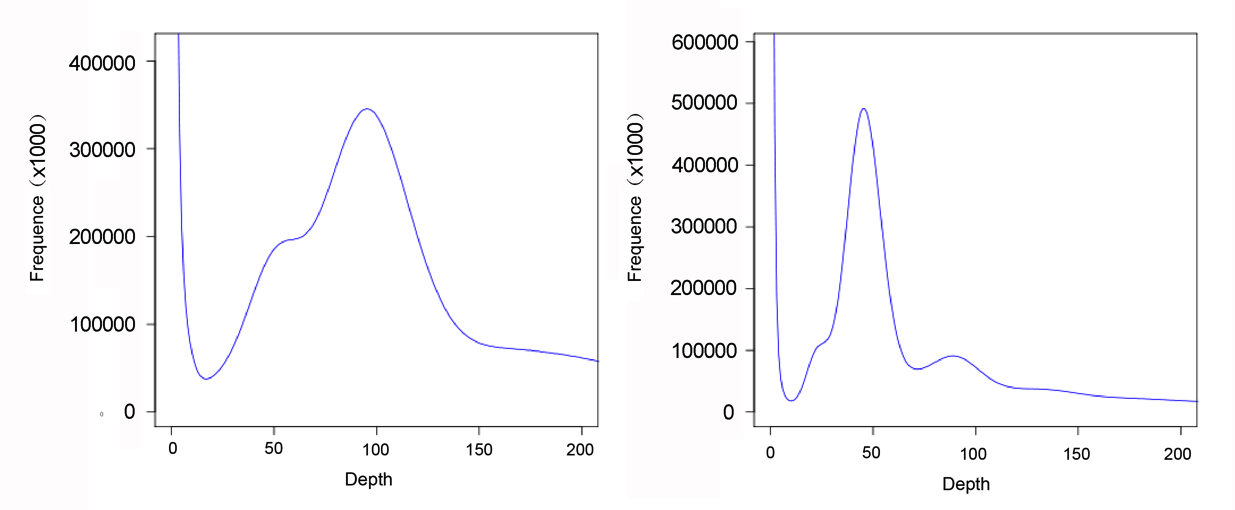


**Fig. S1 Genome size estimation by K-mer analysis of pecan and Chinese hickory.**


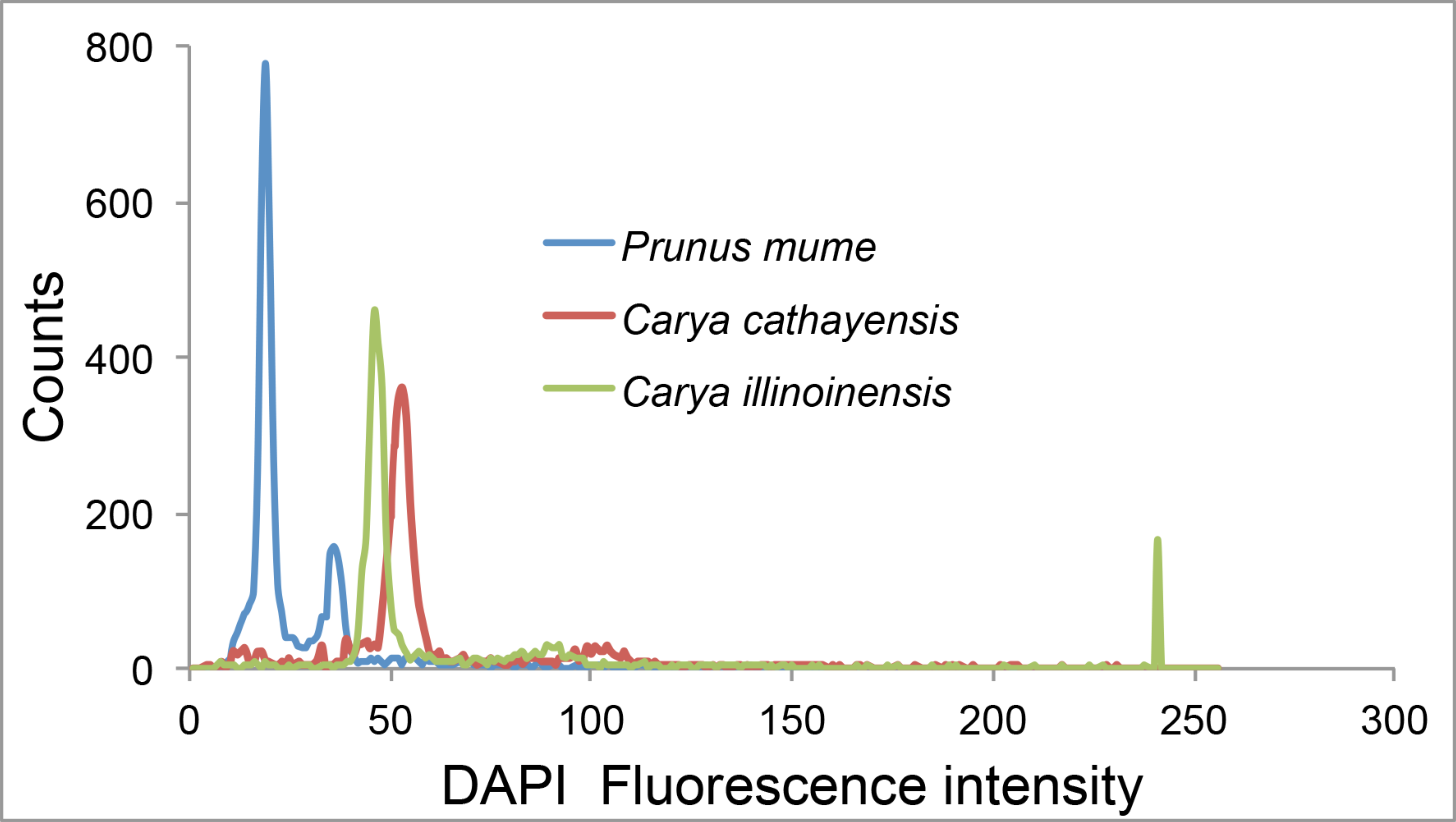


**Fig. S2 The DNA amount peak value of pecan and Chinese hickory.**


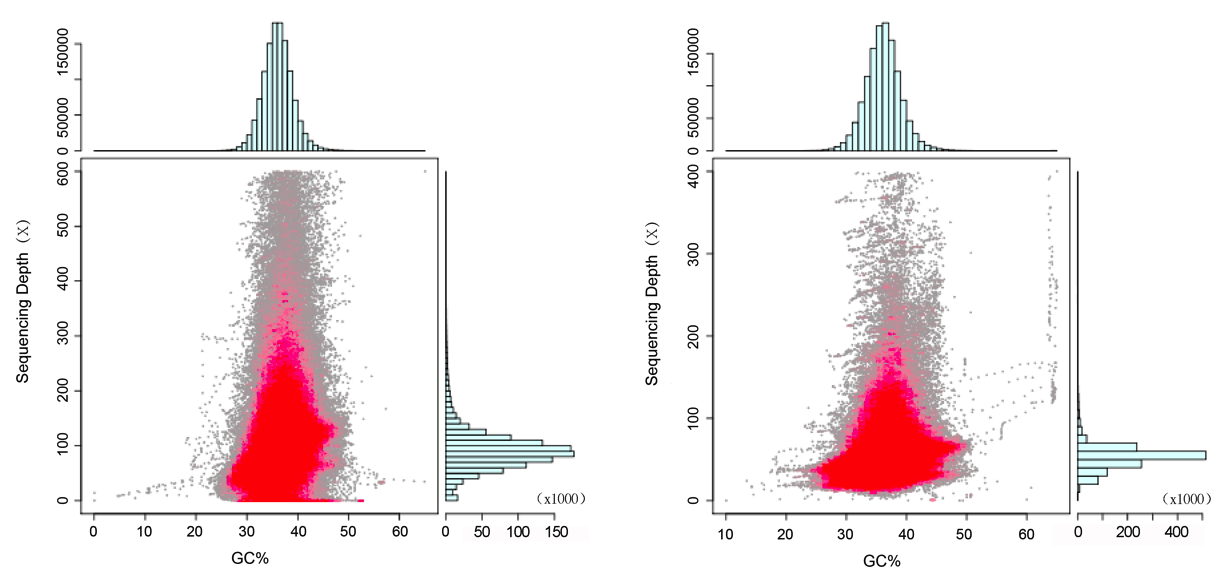


**Fig. S3 The distribution of GC content of pecan and Chinese hickory.**

**Fig. S4 Estimation of LTRs burst in pecan and Chinese hickory.**


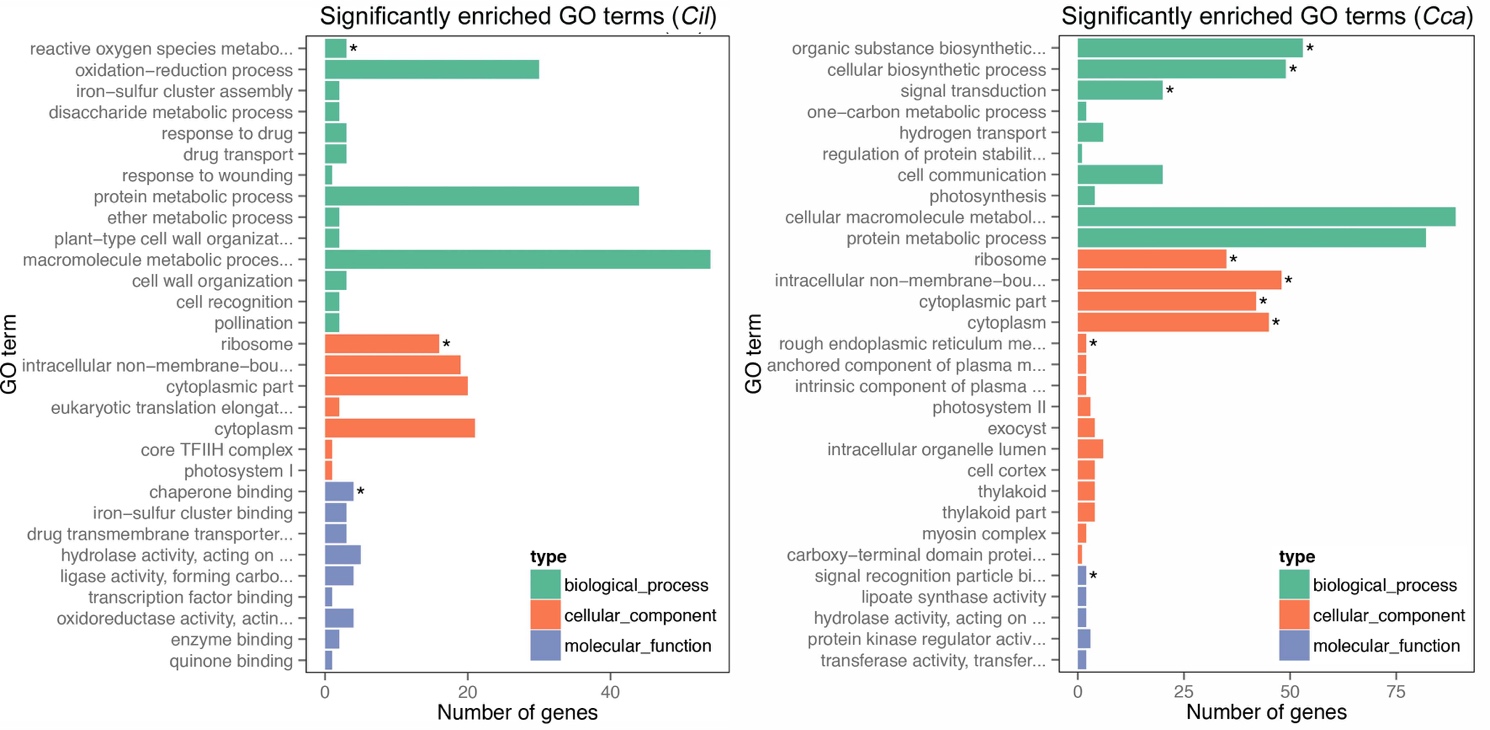


**Fig. S5 Significantly enriched GO terms of pecan- or Chinese hickory- specific genes.**

**Fig. S6 Genes family expansion and contraction analysis.**

**Fig. S7 Significantly enriched GO terms of genes in expanded families.**

**
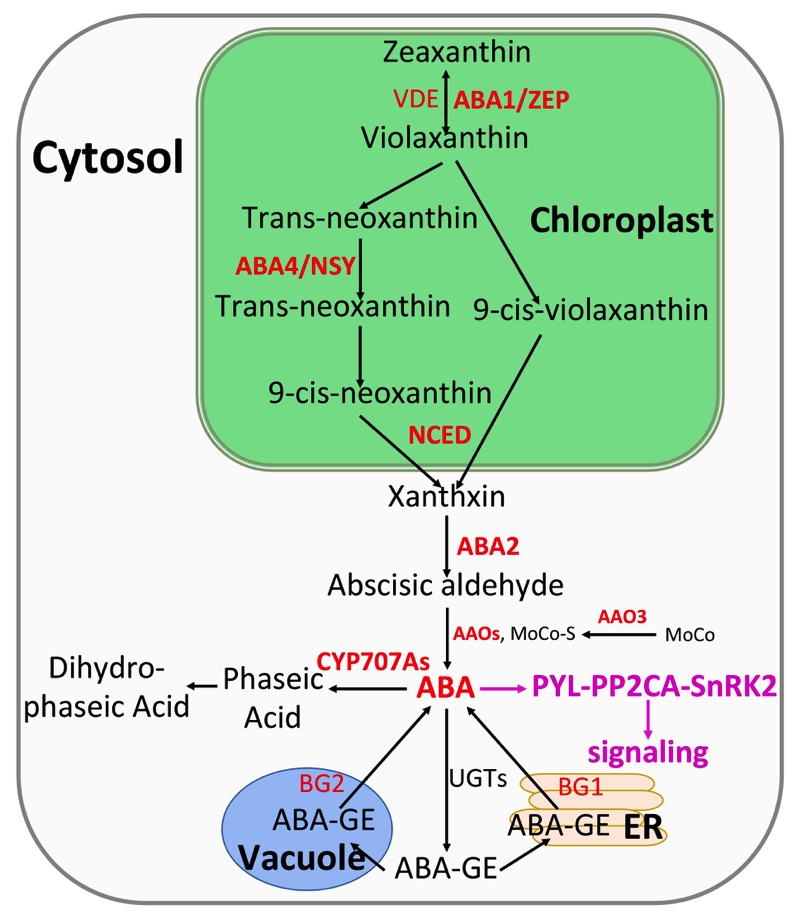
**

**Fig. S8 Schematics of ABA biosynthesis, catabolism and signaling pathway.**


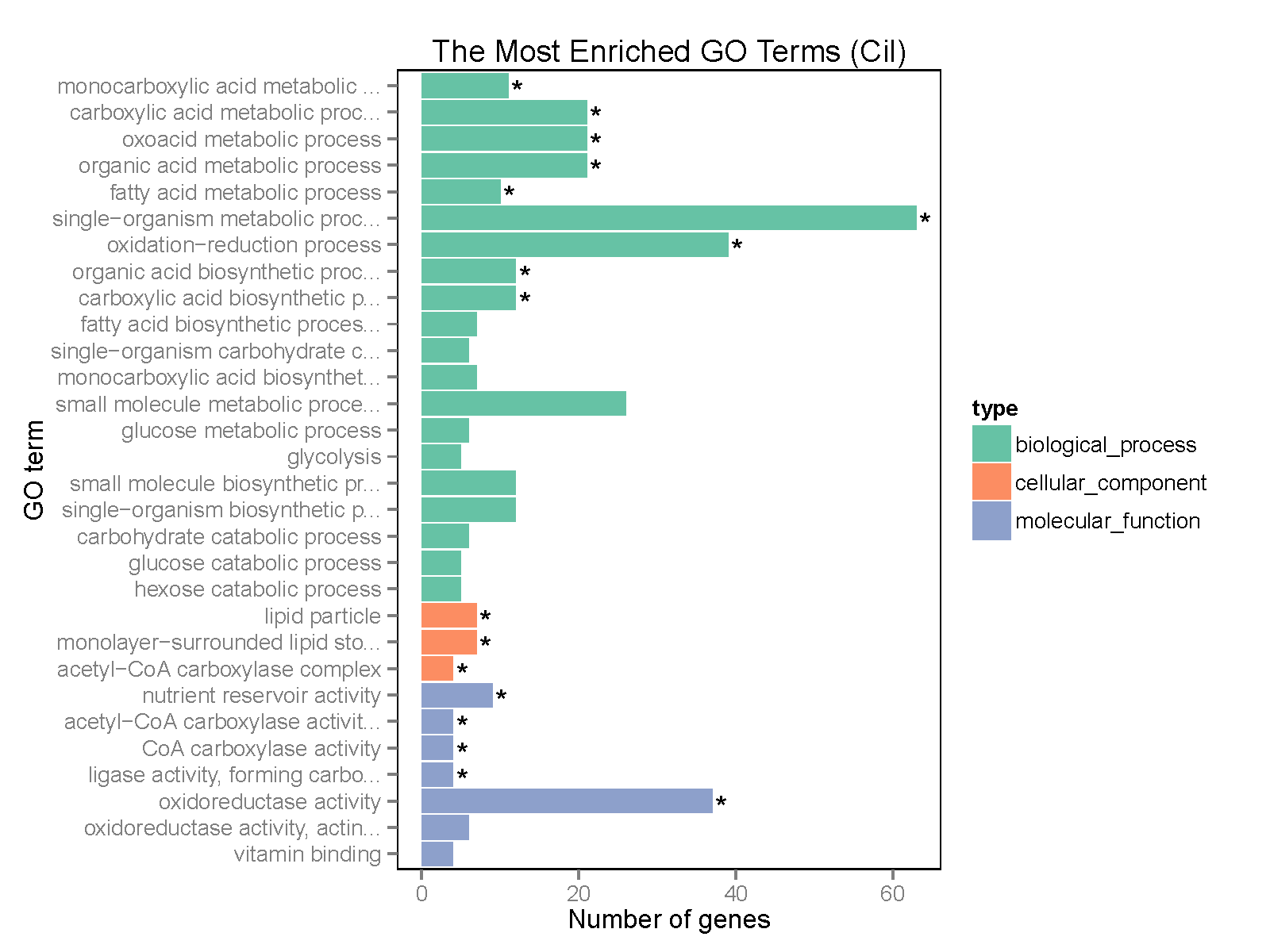

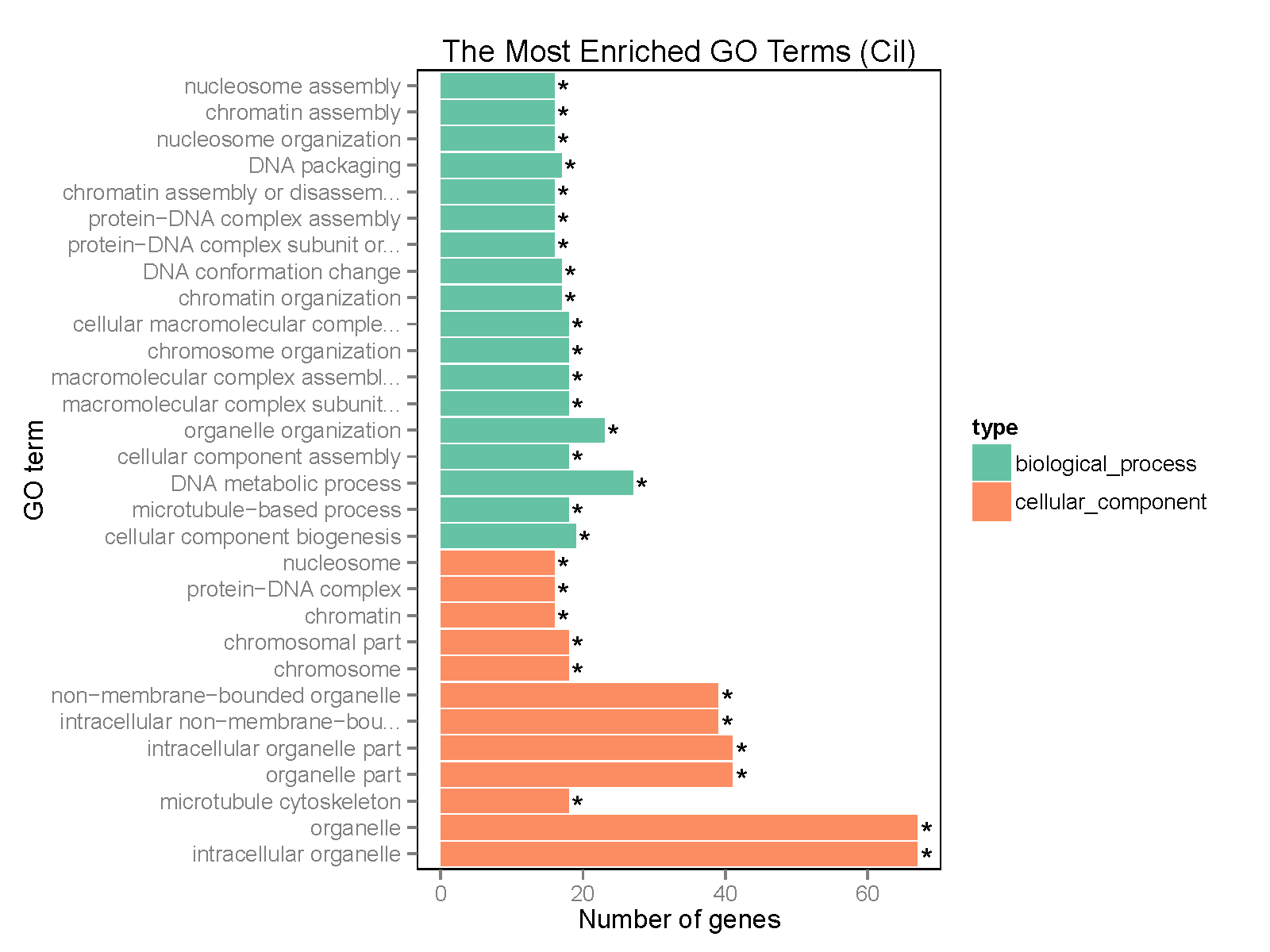


Fig. S9 Significantly enriched GO terms of differentially expressed genes (DEGs) of PEY2 *vs* PEY1 in pecan. Left: down-regulated; right: up-regulated.


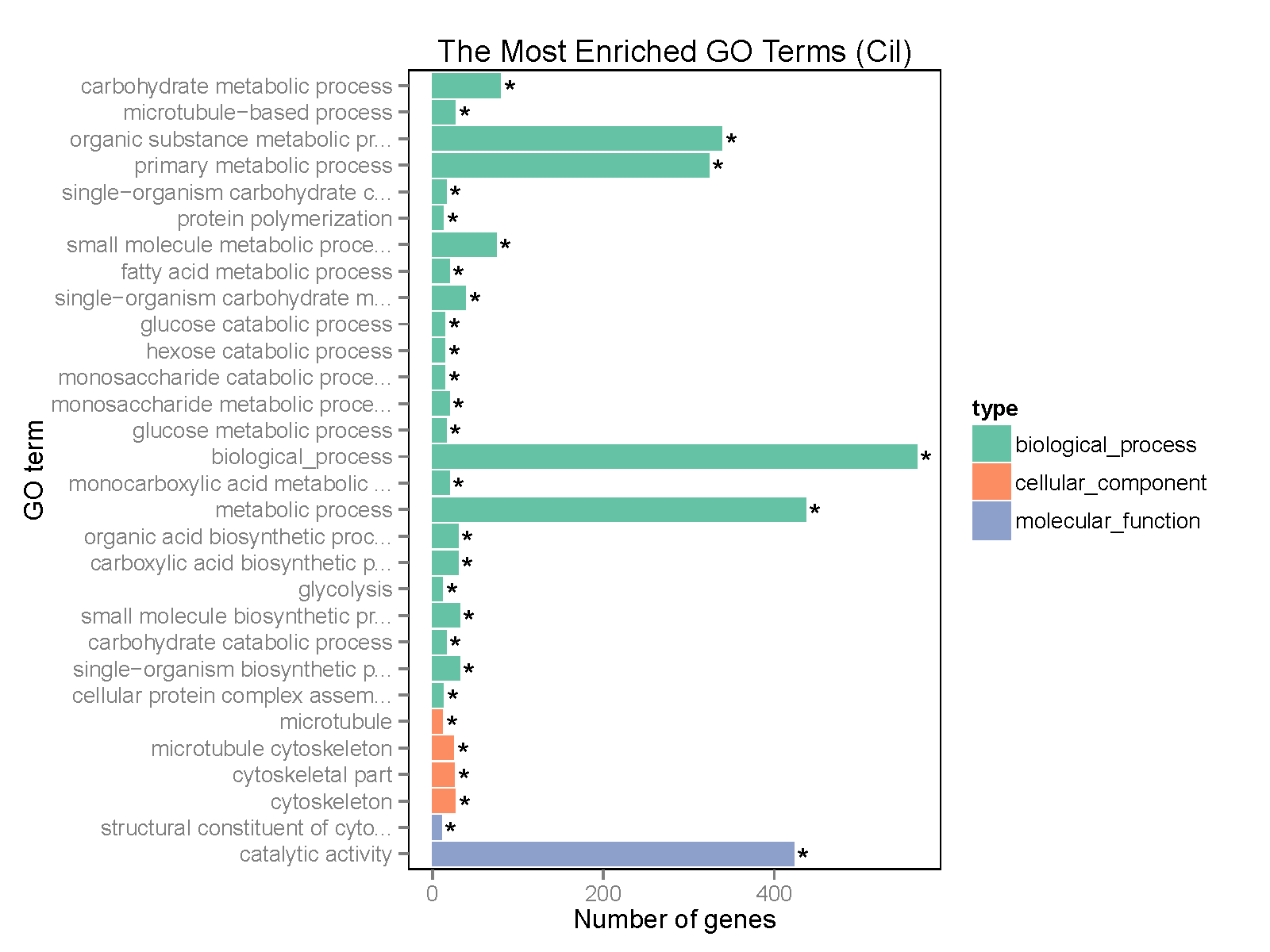

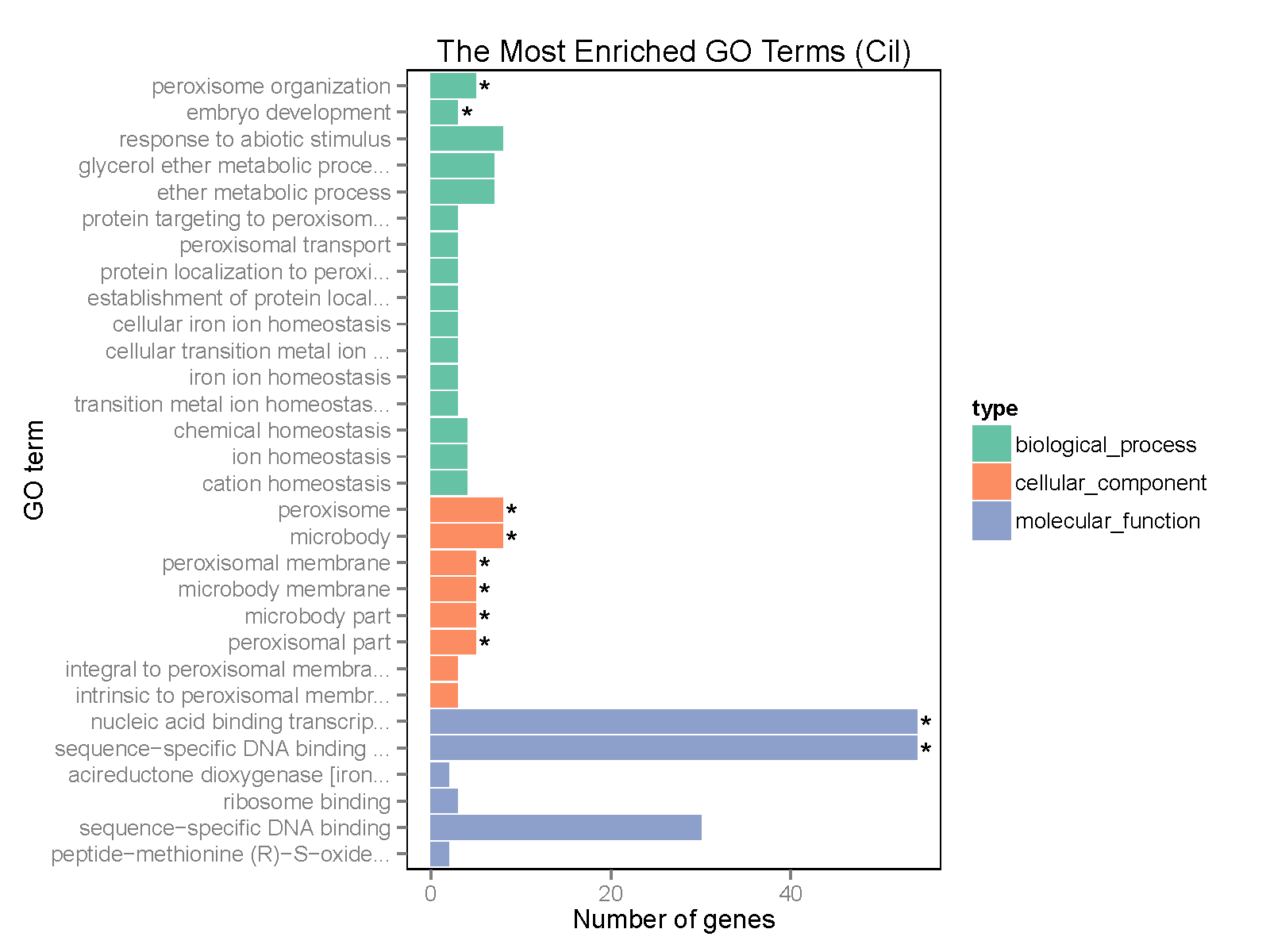


Fig. S10 Significantly enriched GO terms of differentially expressed genes (DEGs) of PEY3 *vs* PEY2 in pecan. Left: down-regulated; right: up-regulated.


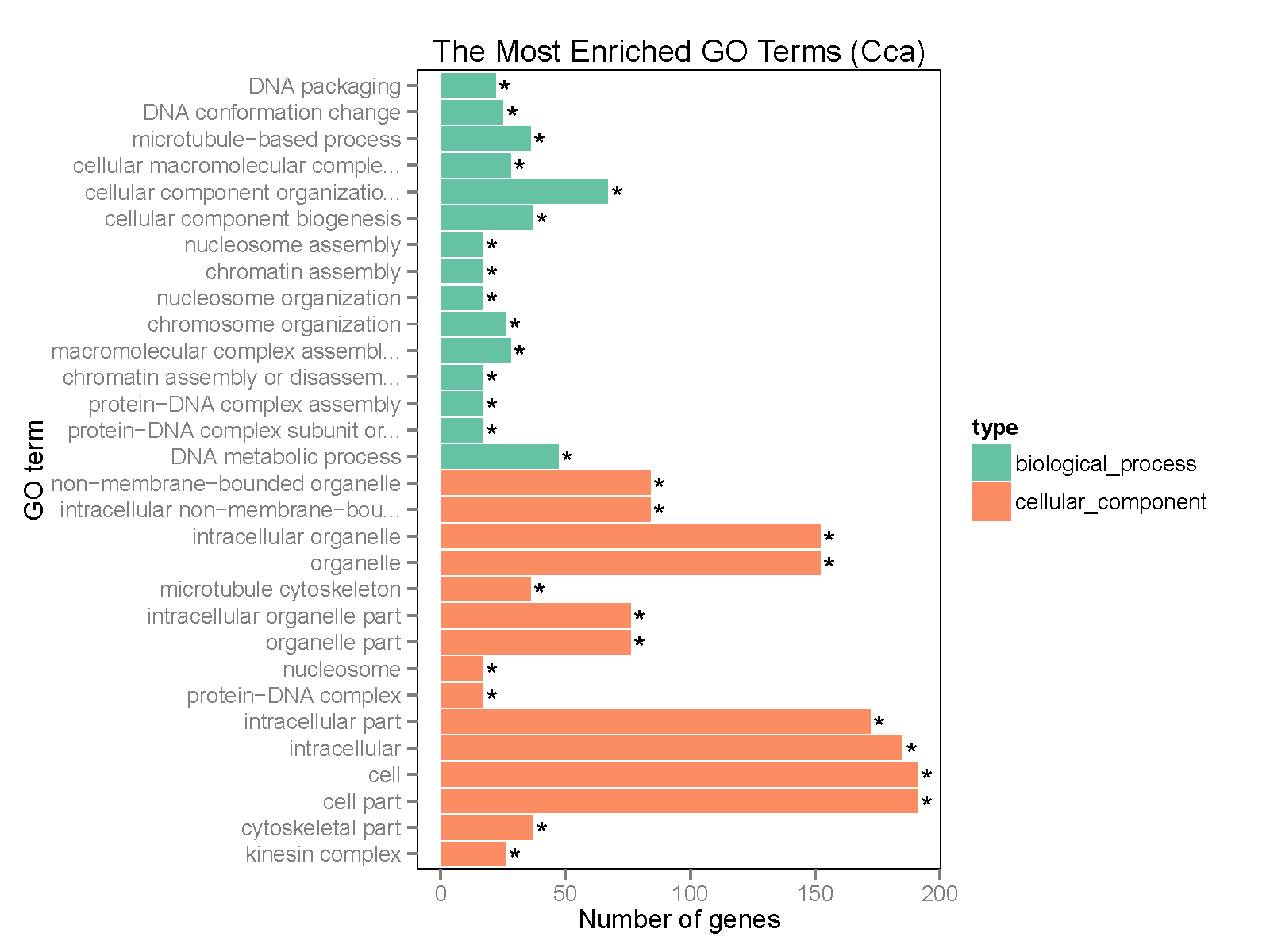


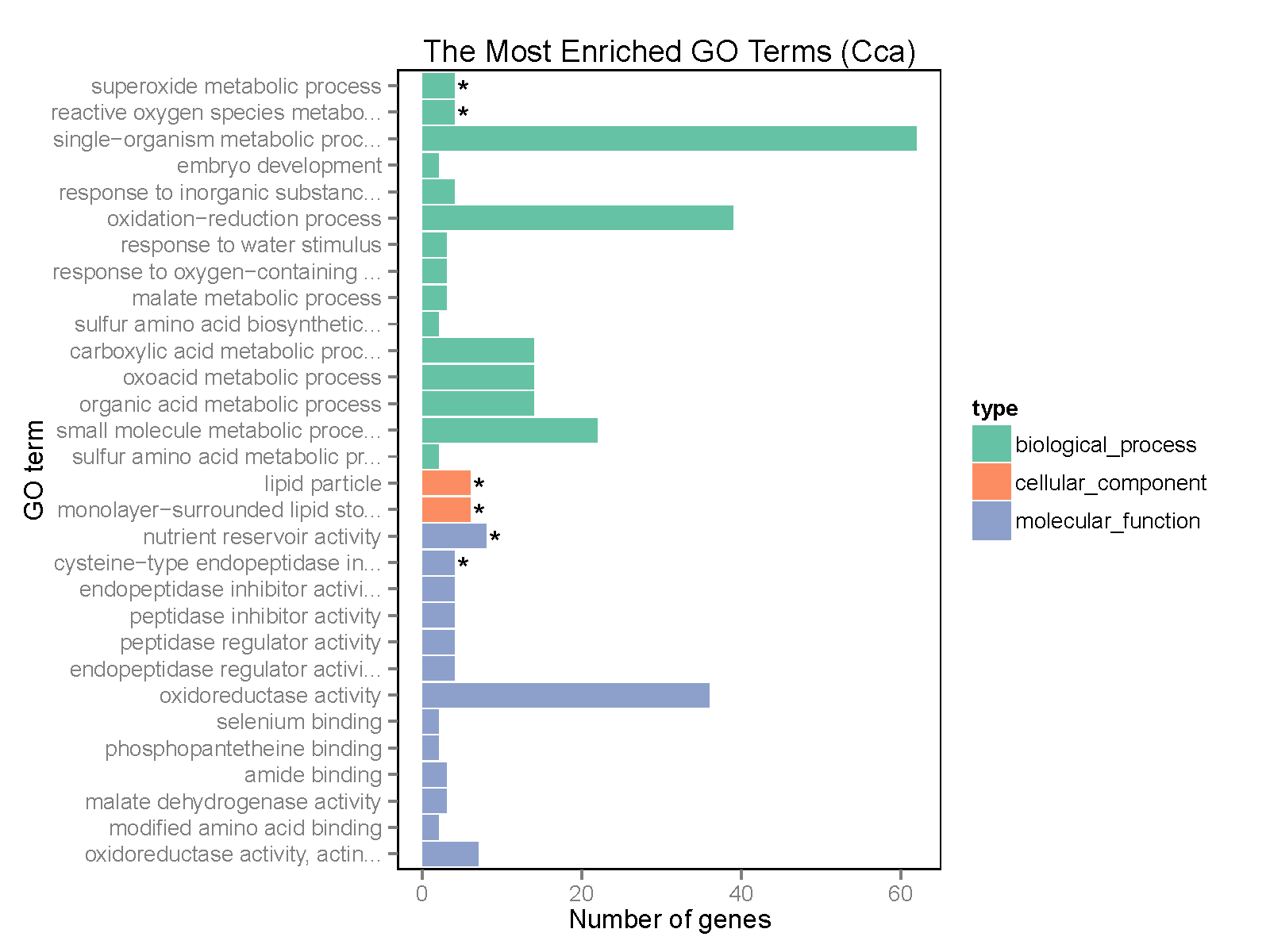


Fig. S11 Significantly enriched GO terms of differentially expressed genes (DEGs) of HEY2 *vs* HEY1 in Chinese hickory. Left: down-regulated; right: up-regulated.


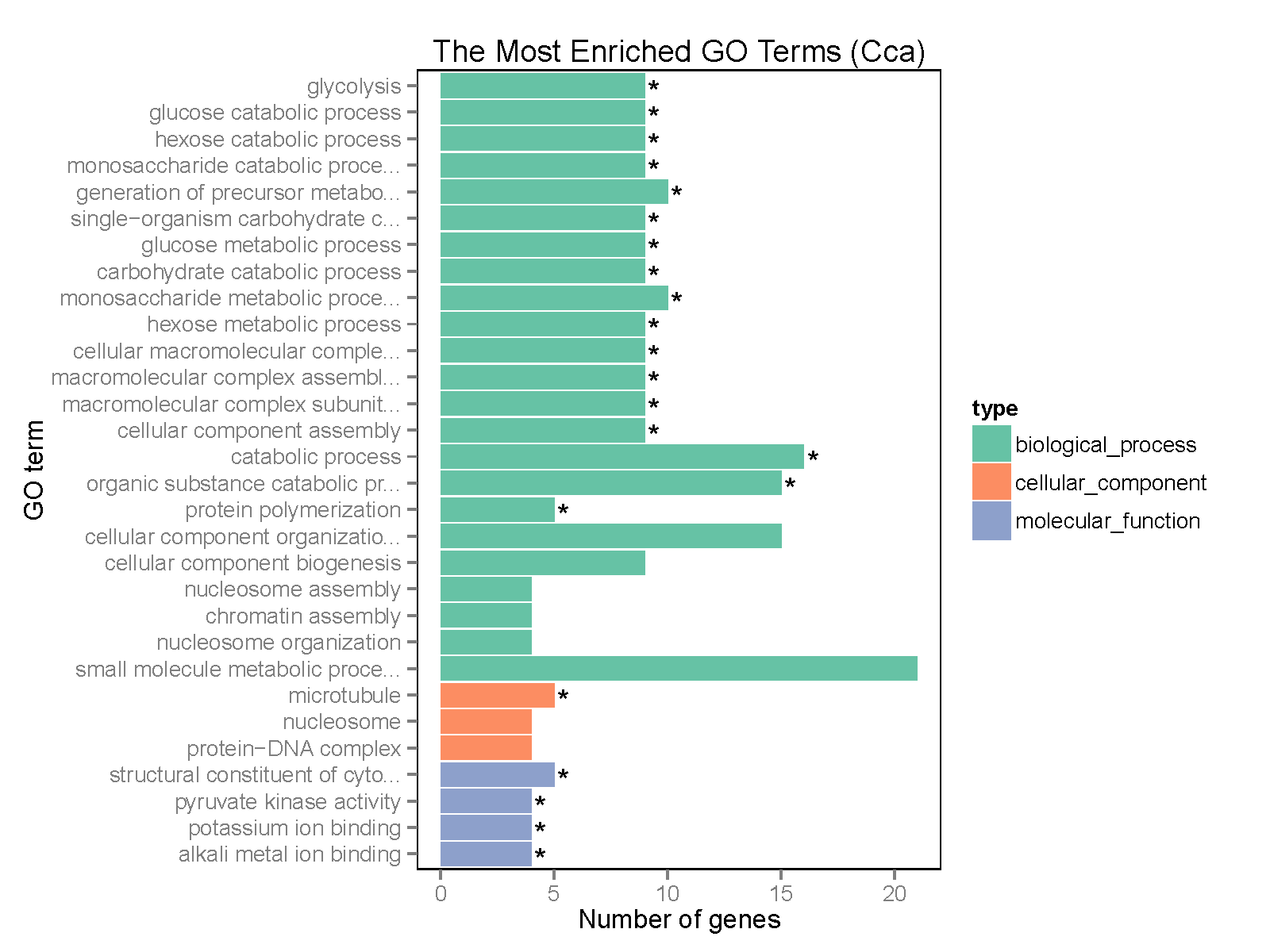

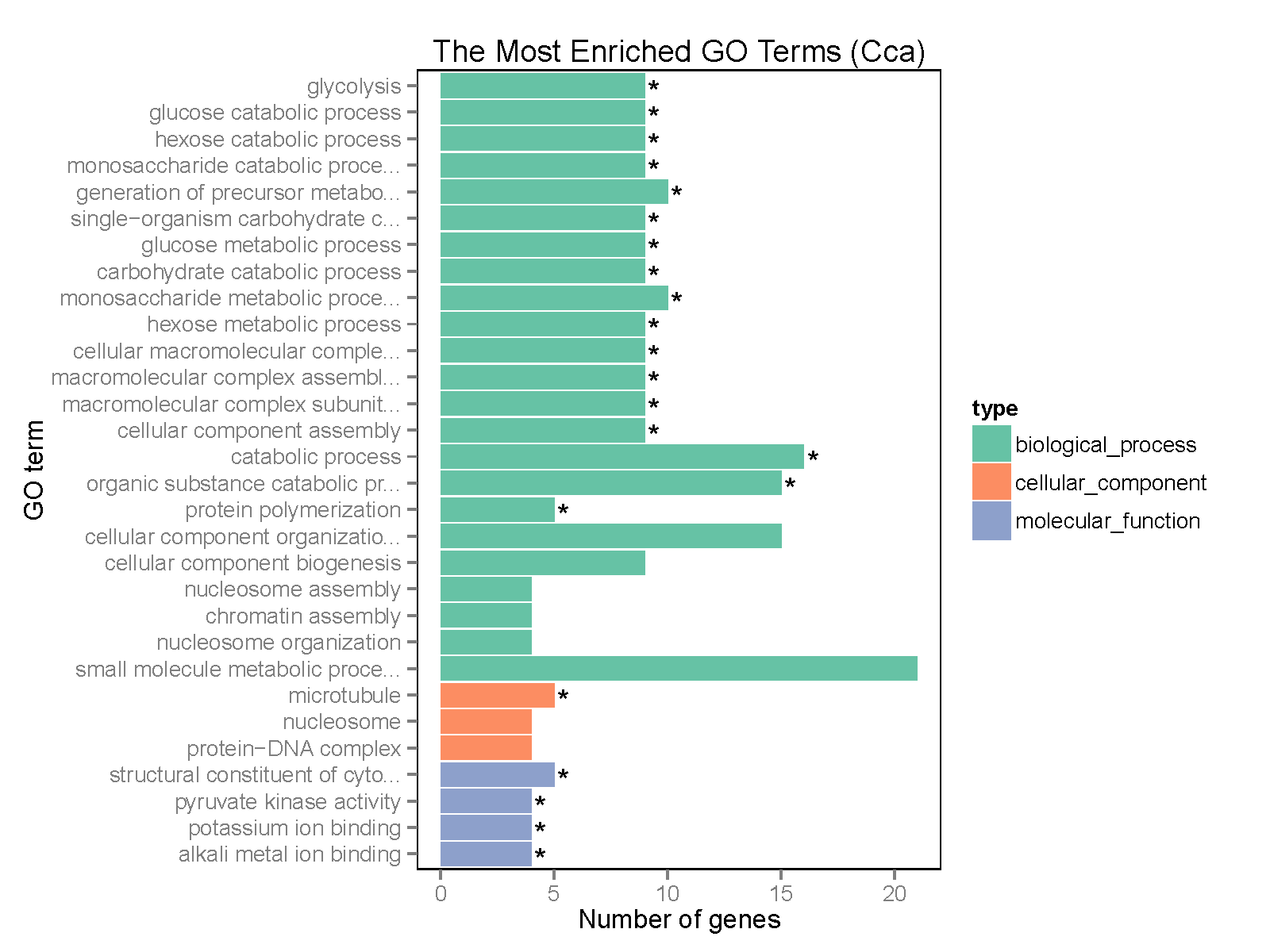

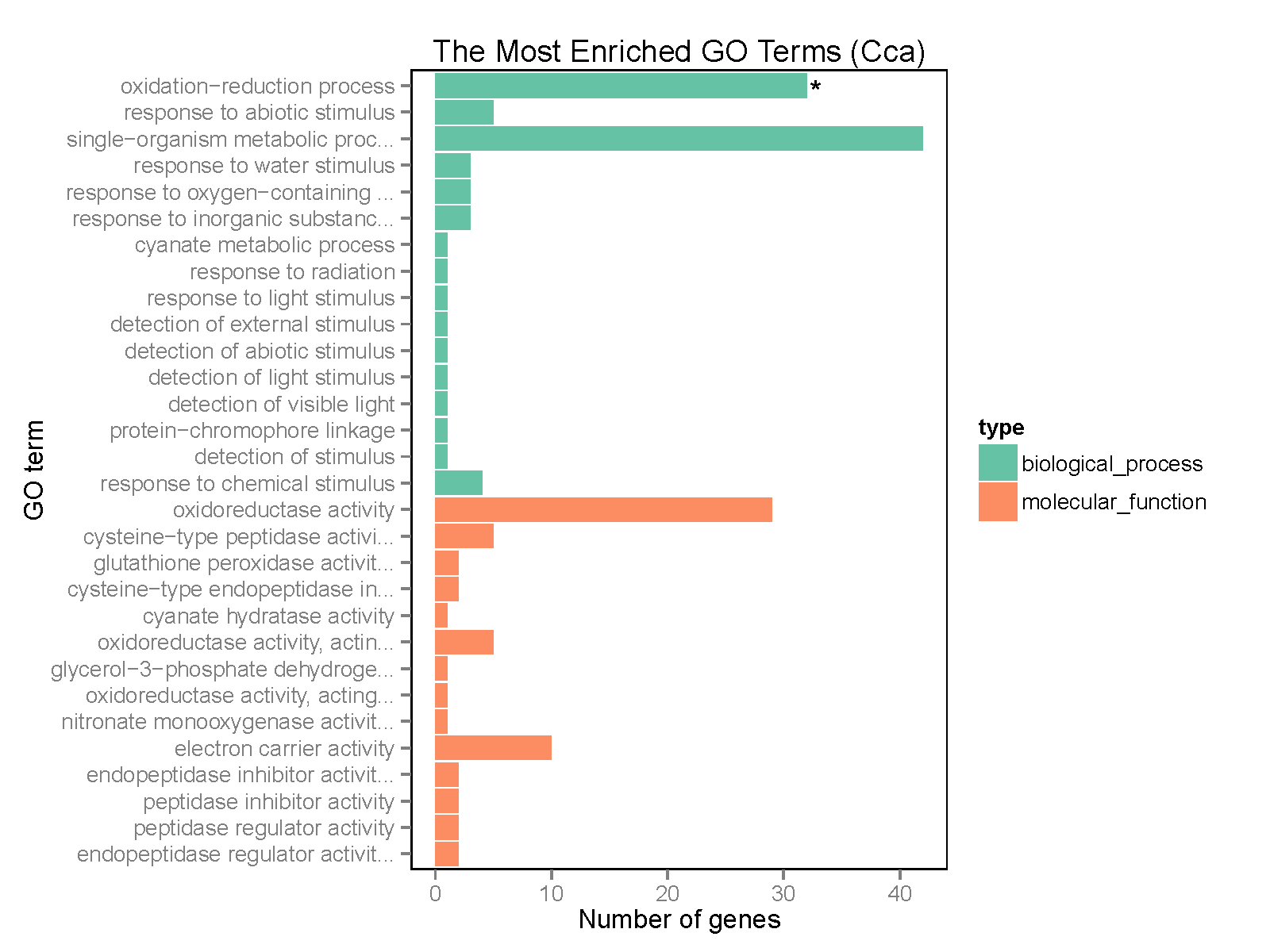


Fig. S12 Significantly enriched GO terms of differentially expressed genes (DEGs) of HEY3 *vs* HEY2 in Chinese hickory. Left: down-regulated; right: up-regulated.

**Fig. S13 Maxmum likelihood tree of chalcone synthase (CHS).**

**Fig. S14 Maxmum likelihood tree of leucoanthocyanidin reductase (LAR).**
