# Supplementary material for: Duphold: scalable, depth-based annotation and curation of high-confidence structural variant calls
Source: Gigascience. 2019 Apr 24;8(4):giz040. doi: 10.1093/gigascience/giz040 (PMC6479422; doi:10.1093/gigascience/giz040)
Supplement: Response_to_Reviewer_Comments_Original_Submission.pdf [file giz040_response_to_reviewer_comments_original_submission.pdf]

Author's Response To Reviewer Comments

Close

Sci-Crunch: SCR_016938

>>> > Reviewer 1
>>> 1. You are mentioning that SV callers such as Lumpy, Manta, Delly etc are not using the coverage information. However, for example in Lumpy you show you are using coverage information for the CNV prediction (Figure 1 A in the Lumpy paper). Furthermore, if I am not mistaken Manta and Delly also leverage coverage information. If not directly then at least for the assessment of the genotypes for each variant where they compare reads that support the reference allele and reads that support the alternative allele. I would suggest to be more precise and I think your point is that you are leveraging coverage information outside of the event. For example, when you take other GC similar regions into account.

We have updated the text to indicate that these methods do not **directly** use depth-of-coverage information within and around the event.

>>> 2. You showed using GiaB call set your performance across deletions. It would be interesting to see the distribution of the DHBFC and DHFFC across the deletions and why you chose the 0.7 as the threshold. Also as a minor comment, please inclue the definition of these terms also in the capture of the Table1.

To address this and other concerns, we have added a new Figure (now figure 1) that shows the distribution of each metric for DEL and DUP events and for randomly chosen
regions of a similar size distribution (with the assumption that these are 0/0 regions). This figure also shows where 0.7 falls on a ROC curve; it appears to be a good
choice to balance sensitivity and specificity. We have also added the definitions to the table caption.

>>> 3. Have you assessed the duphold performance on Lumpy directly without SVTyper? This might show more impact especially on the Precision and thus F1 scores.

We have not done this because the most realistic use-case for lumpy-calls is to have them genotyped by SVTyper. It would show more impact, but would not be
a good indication of the improvement one could achieve relative to commonly-used pipelines.

>>> 4. I would encourage you to make it clearer in the implementation section what are the novel parts of duphold and what has been covered in the mosdepth manuscript. Right now you are stating that the duphold algorithm is explained in the mosdepth manuscript, which makes it hard to assese the novelty of duphold.

We have updated the text to indicate the the coverage calculation is the same as in mosdepth; the novelty is the rapid annotation of an SV VCF with the information in the mosdepth-like coverage profile and the use of the mosdepth-like coverage array to rapidly compare to all regions of similar GC-content.

>>> 5. Are the 250bp windows to measure the GC content overlapping? If so, please state this. If not, why?

These are non-overlapping. As part of our evaluation for these revisions, we checked various window sizes. The sizeof the window did not affect the result, except at very low values. We posit that this may have more influence on samples that were prepared with a protocol using PCR, but, that all that's required is having a sufficient window-size to capturethe coverage changes associated with regional differences in GC-content.

>>> 6. Have you assessed the filtering of duphold on duplications? I know that GiaB is currently not including this type of event, but it would be interesting if duphold can also be used for this. One possibility would be to run Lumpy to detect DUP, subsequently run duphold to filter some of these DUP and maybe visualize some of the filtered DUP with SVPlaudit.

Lumpy called only a single duplication on HG002 and that call was not in the truth-set. We have updated the text to indicate how we converted some INS to DUP and how we simulated homozygous reference DUP and DEL calls so that we could create a ROC curve.

>>> > Reviewer 2
>>> 1. In line 56, author described that depth fold-change would be added to evaluated CNVs, does 'fold-change' equal 'copy number'? If so, duphold should be able to generate genotype information on deletions, and the authors could have benchmarked the genotyping efficiency of this tool; if not, how should user interprete the 'fold' change in terms of actual copy number / genotype prediction?

For a diploid, 2 * fold-change is copy-number. We want to avoid genotyping directly in `duphold` as it is an annotation tool. However, downstream users could use our new figure 1 to decide on reasonable cutoffs for genotyping.

>>> 2. Only deletions from HG002 is benchmarked in this manuscript, while there's not any information about how this tool performs on duplications. Authors should have:
>>> a. evaluated both deletions and duplications , to show performance of duphold on both SV types,

We have added Figure 1 to show the performance on Genome in a Bottle for both deletions and duplications.

>>> b. compare the duphold predicted copy number to the caller predictions and the truth set, and comment if any correction on copy number / predicted SVtype / were achieved by duphold

This is also shown implicitly in figure 1 by indicating the AUC for both heterozygous and hom-alt DUP and DEL calls.

>>> 3. since duphold examined CNV information, the performance is expected to be dependent on size of CNVs evaluated, while this is not discussed in the manuscript at all. Performance of duphold should have been evaluated at different size ranges.

We evaluated 0-1000, 1000-5000, 5000+ and 0+ to get:

0..500
event gt auc n
DUP 0/1 0.75 1246
DUP 1/1 0.74 1109
DEL 0/1 0.87 7407

DEL 1/1 0.94 6307

500..1000
event gt auc n
DUP 0/1 0.64 25
DUP 1/1 0.34 26
DEL 0/1 0.88 453
DEL 1/1 0.96 343

1000..5000
event gt auc n
DUP 0/1 1.00 6
DUP 1/1 0.00 3
DEL 0/1 0.97 773
DEL 1/1 1.00 634

5000+
event gt auc n
DUP 0/1 0.00 0
DUP 1/1 0.00 0
DEL 0/1 0.97 262
DEL 1/1 1.00 209

0+ (as shown in paper).
event gt auc n
DUP 0/1 0.74 1277
DUP 1/1 0.73 1138
DEL 0/1 0.89 8895
DEL 1/1 0.94 7493

So, duphold performs better for deletions > 1KB but the number of duplications
is too small to find a real trend (though we should expect improving accuracy with size).

We have added this important observation to the text.

>>> 4. It is mentioned in line 18-19 that 500Mb is required to collect depth information from chromosome 1, and length of whole genome measures at 12X of chr1 (3Gb vs. 250Mb), so does it requires 6Gb memory to process a sample? Or can all chromosomes be processed in parallel while at maximum 500Mb is required for each job?

We have updated the text to clarify this. The maximum memory use would be ~500MB because each chromosome is evaluated sequentially.The parallelism comes from bam decompression, not during the coverage calculation.

>>> 5. Run time is estimated at <15 CPU-minutes for a 25X whole genome, again, is this measured at whole genome or each chromosome in parallel ?

This is the CPU time, not the user-time so with a single thread, it will take around 15 minutes to process a 25X genome.

>>> 6. How do run time increase with sequence depth? Linearly?

Yes. Linearly. We have updated the text to note this.

>>> 7. In line 53, it's described that duphold compare median depth of predicted CNV to the depth of 5Kb flanking regions on either side, so which (left / right) side were examined ? how's the decision made? And how would this tool perform on CNVs where breakpoints were off (so that the flanking regions would show similar depth to predicted event thus false negative would have been generated) ?

duphold compares the median depth inside to the median of all bases in the left and right flanks. If the breakpoints are too far off, this will not work.
However, using the median (instead of mean) allows some deviance in the reported breakpoints without affecting the estimate.

The 5kb flanking region was chosen to have enough bases to get a good estimate without extending so far as to increase the probability of hitting another
event. In response this this review, and users with genomes that have less complete assemblies (more regions with sparse coverage), we evaluated 500, 1000, and 5000 base windows
using the setup for the new figure 1. We found that the AUC (from the ROC plot) for DELs is relatively unaffected by the window size. For DUPs, 5000 actually had lower AUC for
5000 than for 1000 or 500. We therefore dropped the default to 1000 bases and we have exposed this as a parameter that can be adjusted via an environment variable.

Close
