# Supplementary material for: Duphold: scalable, depth-based annotation and curation of high-confidence structural variant calls
Source: Gigascience. 2019 Apr 24;8(4):giz040. doi: 10.1093/gigascience/giz040 (PMC6479422; doi:10.1093/gigascience/giz040)
Supplement: Response_to_Reviewer_Comments_Revision_1.pdf [file giz040_response_to_reviewer_comments_revision_1.pdf]

Author's Response To Reviewer Comments

Close

> one question regarding the new results: is there an explanation why AUC of homozygous duplication (0.73) lower than it of heterozygous dup (0.74)?

This is likely due to th method we used to "create" duplications from GiaB insertion calls. when we create a DUP, we check that the purported INS maps next to the reported position. So, single-copy calls are likely to be very good and a bit more strict. That is, we know that if we called it a DUP, there is at least 1 copy of homology that can be found with illumina reads. It is not guaranteed for what GiaB calls as homozygous (1/1) INS since it could be that only a single copy can be found with illumina reads and not the expected 2 copies.
It is also likely affected by the smaller numbers of homozygous DUP (INS) calls.

Close
