# Supplementary material for: Duphold: scalable, depth-based annotation and curation of high-confidence structural variant calls
Source: Gigascience. 2019 Apr 24;8(4):giz040. doi: 10.1093/gigascience/giz040 (PMC6479422; doi:10.1093/gigascience/giz040)
Supplement: Reviewer_1_Report_Original_Submission -- Fritz Sedlazeck — 12/20/2018 Reviewed [file giz040_reviewer_1_report_original_submission.pdf]

Reviewer Report

Title: duphold: scalable, depth-based annotation and curation of high-confidence structural variant calls

Version: Original Submission **Date:** 12/20/2018

Reviewer name: Fritz Sedlazeck

Reviewer Comments to Author:

The authors of the manuscript "duphold: scalalable, depth-based annotation and curation of high-confidence structural variant calls" describes a new method to leverage the coverage information across deletion calls to infer their reliability. This is done mainly by comparing the coverage level inside of the deletion vs. the coverage of the 5k flanking regions or regions with similar GC content. They highlight an impressive performance of duphold when scaling up to multiple thousands of SVs, which are likely needed for population scale SVs calling. The manuscript is well written and easy to follow. In the following I will list my questions and concerns:

1. You are mentioning that SV callers such as Lumpy, Manta, Delly etc are not using the coverage information. However, for example in Lumpy you show you are using coverage information for the CNV prediction (Figure 1 A in the Lumpy paper). Furthermore, if I am not mistaken Manta and Delly also leverage coverage information. If not directly then at least for the assessment of the genotypes for each variant where they compare reads that support the reference allele and reads that support the alternative allele. I would suggest to be more precise and I think your point is that you are leveraging coverage information outside of the event. For example, when you take other GC similar regions into account.
2. You showed using GiaB call set your performance across deletions. It would be interesting to see the distribution of the DHBFC and DHFFC across the deletions and why you chose the 0.7 as the threshold. Also as a minor comment, please inclue the definition of these terms also in the capture of the Table1.
3. Have you assessed the duphold performance on Lumpy directly without SVTyper? This might show more impact especially on the Precision and thus F1 scores.
4. I would encourage you to make it clearer in the implementation section what are the novel parts of duphold and what has been covered in the mosdepth manuscript. Right now you are stating that the duphold algorithm is explained in the mosdepth manuscript, which makes it hard to assesse the novelty of duphold.
5. Are the 250bp windows to measure the GC content overlapping? If so, please state this. If not, why?
6. Have you assessed the filtering of duphold on duplications? I know that GiaB is currently not including this type of event, but it would be interesting if duphold can also be used for this. One possibility would be to run Lumpy to detect DUP, subsequently run duphold to filter some of these DUP and maybe visualize some of the filtered DUP with SVPlaudit.

I hope this helps.
Fritz Sedlazeck

Level of Interest

Please indicate how interesting you found the manuscript: Choose an item.

Quality of Written English

Please indicate the quality of language in the manuscript: Choose an item.

Declaration of Competing Interests

Please complete a declaration of competing interests, considering the following questions:

- Have you in the past five years received reimbursements, fees, funding, or salary from an organisation that may in any way gain or lose financially from the publication of this manuscript, either now or in the future?
- Do you hold any stocks or shares in an organisation that may in any way gain or lose financially from the publication of this manuscript, either now or in the future?
- Do you hold or are you currently applying for any patents relating to the content of the manuscript?
- Have you received reimbursements, fees, funding, or salary from an organization that holds or has applied for patents relating to the content of the manuscript?
- Do you have any other financial competing interests?
- Do you have any non-financial competing interests in relation to this paper?

If you can answer no to all of the above, write 'I declare that I have no competing interests' below. If your reply is yes to any, please give details below.

I declare that I have no competing interests

I agree to the open peer review policy of the journal. I understand that my name will be included on my report to the authors and, if the manuscript is accepted for publication, my named report including any attachments I upload will be posted on the website along with the authors' responses. I agree for my report to be made available under an Open Access Creative Commons CC-BY license (http://creativecommons.org/licenses/by/4.0/). I understand that any comments which I do not wish to be included in my named report can be included as confidential comments to the editors, which will not be published.

I agree to the open peer review policy of the journal

To further support our reviewers, we have joined with Publons, where you can gain additional credit to further highlight your hard work (see: https://publons.com/journal/530/gigascience). On publication of this paper, your review will be automatically added to Publons, you can then choose whether or not to claim your Publons credit. I understand this statement.

Yes Choose an item.
