# Supplementary material for: Duphold: scalable, depth-based annotation and curation of high-confidence structural variant calls
Source: Gigascience. 2019 Apr 24;8(4):giz040. doi: 10.1093/gigascience/giz040 (PMC6479422; doi:10.1093/gigascience/giz040)
Supplement: Reviewer_2_Report_Original_Submission -- Xuefang Zhao — 1/13/2019 Reviewed [file giz040_reviewer_2_report_original_submission.pdf]

Reviewer Report

Title: duphold: scalable, depth-based annotation and curation of high-confidence structural variant calls

Version: Original Submission **Date:** 1/13/2019

Reviewer name: Xuefang Zhao

Reviewer Comments to Author:

This manuscript represent a new tool named ' duphold' which evaluate copy number variation (CNV) calls with read depth information. This tool can discriminate true positive versus false positive CNVs, thus reducing the false discovery rate of CNV callsets. The feature of this tool, as shown in figure1, that run time do not linearly increase with number of variants, makes it feasible for large scale genomic studies where CNVs would need to be tested and genotyped across thousands of samples. Overall this is a useful tool that has the potential to be applied in large genomic studies, while more details of the method and benchmark should have been provided.
comments:
1. In line 56, author described that depth fold-change would be added to evaluated CNVs, does 'fold-change' equal 'copy number'? If so, duphold should be able to generate genotype information on deletions, and the authors could have benchmarked the genotyping efficiency of this tool; if not, how should user interprete the 'fold' change in terms of actual copy number / genotype prediction?
2. Only deletions from HG002 is benchmarked in this manuscript, while there's not any information about how this tool performs on duplications. Authors should have:
a. evaluated both deletions and duplications , to show performance of duphold on both SV types,
b. compare the duphold predicted copy number to the caller predictions and the truth set, and comment if any correction on copy number / predicted SVtype / were achieved by duphold
3. since duphold examined CNV information, the performance is expected to be dependent on size of CNVs evaluated, while this is not discussed in the manuscript at all. Performance of duphold should have been evaluated at different size ranges.
4. It is mentioned in line 18-19 that 500Mb is required to collect depth information from chromosome 1, and length of whole genome measures at 12X of chr1 (3Gb vs. 250Mb), so does it requires 6Gb memory to process a sample? Or can all chromosomes be processed in parallel while at maximum 500Mb is required for each job?
5. Run time is estimated at <15 CPU-minutes for a 25X whole genome, again, is this measured at whole genome or each chromosome in parallel ?
6. How do run time increase with sequence depth? Linearly?
7. In line 53, it's described that duphold compare median depth of predicted CNV to the depth of 5Kb flanking regions on either side, so which (left / right) side were examined ? how's the decision made? And how would this tool perform on CNVs where breakpoints were off (so that the flanking regions would show similar depth to predicted event thus false negative would have been generated) ?

Level of Interest

Please indicate how interesting you found the manuscript: Choose an item.

Quality of Written English

Please indicate the quality of language in the manuscript: Choose an item.

Declaration of Competing Interests

Please complete a declaration of competing interests, considering the following questions:

- Have you in the past five years received reimbursements, fees, funding, or salary from an organisation that may in any way gain or lose financially from the publication of this manuscript, either now or in the future?
- Do you hold any stocks or shares in an organisation that may in any way gain or lose financially from the publication of this manuscript, either now or in the future?
- Do you hold or are you currently applying for any patents relating to the content of the manuscript?
- Have you received reimbursements, fees, funding, or salary from an organization that holds or has applied for patents relating to the content of the manuscript?
- Do you have any other financial competing interests?
- Do you have any non-financial competing interests in relation to this paper?

If you can answer no to all of the above, write 'I declare that I have no competing interests' below. If your reply is yes to any, please give details below.

I declare that I have no competing interests

I agree to the open peer review policy of the journal. I understand that my name will be included on my report to the authors and, if the manuscript is accepted for publication, my named report including any attachments I upload will be posted on the website along with the authors' responses. I agree for my report to be made available under an Open Access Creative Commons CC-BY license (http://creativecommons.org/licenses/by/4.0/). I understand that any comments which I do not wish to be included in my named report can be included as confidential comments to the editors, which will not be published.

I agree to the open peer review policy of the journal

To further support our reviewers, we have joined with Publons, where you can gain additional credit to further highlight your hard work (see: https://publons.com/journal/530/gigascience). On publication of this paper, your review will be automatically added to Publons, you can then choose whether or not to claim your Publons credit. I understand this statement.

Yes Choose an item.
